# Improving machine learning reproducibility in genetic association studies with proportional instance cross validation (PICV)

**DOI:** 10.1186/s13040-018-0167-7

**Published:** 2018-04-19

**Authors:** Elizabeth R. Piette, Jason H. Moore

**Affiliations:** 1 Graduate Group in Genomics and Computational Biology, Perelman School of Medicine, University of Pennsylvania, Philadelphia, PA USA; 2 Institute for Biomedical Informatics, Perelman School of Medicine, University of Pennsylvania, Philadelphia, PA USA

**Keywords:** Cross validation, Machine learning, Epistasis, GWAS

## Abstract

**Background:**

Machine learning methods and conventions are increasingly employed for the analysis of large, complex biomedical data sets, including genome-wide association studies (GWAS). Reproducibility of machine learning analyses of GWAS can be hampered by biological and statistical factors, particularly so for the investigation of non-additive genetic interactions. Application of traditional cross validation to a GWAS data set may result in poor consistency between the training and testing data set splits due to an imbalance of the interaction genotypes relative to the data as a whole. We propose a new cross validation method, proportional instance cross validation (PICV), that preserves the original distribution of an independent variable when splitting the data set into training and testing partitions.

**Results:**

We apply PICV to simulated GWAS data with epistatic interactions of varying minor allele frequencies and prevalences and compare performance to that of a traditional cross validation procedure in which individuals are randomly allocated to training and testing partitions. Sensitivity and positive predictive value are significantly improved across all tested scenarios for PICV compared to traditional cross validation. We also apply PICV to GWAS data from a study of primary open-angle glaucoma to investigate a previously-reported interaction, which fails to significantly replicate; PICV however improves the consistency of testing and training results.

**Conclusions:**

Application of traditional machine learning procedures to biomedical data may require modifications to better suit intrinsic characteristics of the data, such as the potential for highly imbalanced genotype distributions in the case of epistasis detection. The reproducibility of genetic interaction findings can be improved by considering this variable imbalance in cross validation implementation, such as with PICV. This approach may be extended to problems in other domains in which imbalanced variable distributions are a concern.

**Electronic supplementary material:**

The online version of this article (10.1186/s13040-018-0167-7) contains supplementary material, which is available to authorized users.

## Background

Genome-wide association studies (GWAS) have been frequently critiqued for failing to explain the “missing heritability” of complex disease in terms of single-locus main effects [1, 2]. In addition to interrogating the contributions of rare variants, non-coding regions, structural variation, etc., a logical reactionary paradigm to embrace involves revisiting heritability estimates to consider the effect of interactions and developing approaches that acknowledge that loci do not exist in isolation but rather act in complex networks of interacting partners in the dynamic, three-dimensional genome and in tissue-specific and environmental context [3–6]. Utilizing pre-existing GWAS data to test a curated set of potentially biologically-relevant interactions, such as those identified as being plausible via expert knowledge, integrating data from gene set enrichment analyses, chromatin capture experiments, co-expression data sets, etc. provides a way to overcome the multiple testing burden of naively testing every possible interaction and motivates future bench science experimentation [7, 8]. Accordingly, machine learning methods are appealing for the analysis of this big, complex data, and have been applied to diverse problems and data types across the biological sciences [9, 10]. However, machine learning should not be viewed as a panacea that can be readily applied to all genomics problems. Beyond concerns regarding model choice and interpretability, there are numerous reasons why valid biological interactions may fail to appear statistically significant and vice versa [11–13]. Therefore, typical machine learning tools, techniques, and standards from other fields may need tweaking to be appropriate for use in genomics considering the unique biases in generating genomic data sets, the structure of the genome, the validity of model assumptions, etc.

Improving the reproducibility of machine learning analyses of genomic data will require methodological and analytic advances from not only both the computational and wet laboratory sides, but also their consideration in conjunction with each other as a greater whole. Sharing data publicly for secondary analyses, writing open-source code in executable notebook format, and using container and cloud services all contribute to a culture of reproducibility that enhances the capacity for integrative and innovative computational analyses [14–17]. Likewise, thoughtfully interrogating methodological, environmental, and other determinants of inconsistencies in bench experimentation results lends robustness to findings, and this greater understanding of sources of variation can in itself lead to worthwhile new hypotheses [18]. Ideally, technological supports such as mobile applications for data collection will increasingly allow for recording more complete and consistent data in a format that can be seamlessly analyzed with software tools developed or modified to consider the unique intricacies of the data at hand [19].

Epistasis, or the non-additive interaction between genotypes to produce phenotype, is difficult to detect statistically but is of biological interest in light of a multifactorial view of disease [20–22]. This study is motivated by poor cross-validation performance observed for epistasis data sets with an interaction between two single nucleotide polymorphisms (SNPs). A given SNP may be represented as a categorical variable with possible values of 0, 1, or 2 corresponding to doses of the minor allele, and by extension the interaction of two SNPs may be represented in terms of 9 categories reflecting the identities of SNP1 and SNP2. Internal cross validation is a widely-used standard for evaluating the performance of a machine learning analysis in which the data is split into two mutually exclusive partitions, a model is fit using the ‘training’ set, and its performance is evaluated on predicting the classes of the held out set of observations (the ‘testing’ or ‘validation’ set; not to be confused with an external independent replication data set which may also be referred to as ‘testing’ or ‘validation’) [23]. Typically the overall data set is split such that the resultant training and testing partitions are random, independent draws from the same probability distribution, although there are also methods that consider the data structure, generally in terms of maintaining outcome class proportions between the training and testing data sets [24–26]. In this study, we propose a new cross validation method, proportional instance cross validation (PICV), that preserves the relative distribution of an independent variable (in our example application, SNP-SNP interaction genotypes) when dividing the overall data set into train and test partitions. We demonstrate significantly improved sensitivity and positive predictive value across all tested scenarios with application of PICV relative to a traditional cross validation implementation. We additionally apply PICV to primary open-angle glaucoma GWAS data to investigate an interaction previously reported to be significant in two independent data sets. Although this interaction is not observed to be significant in our analysis, PICV produced more consistent estimates than a traditional cross validation implementation. This approach is not only appropriate for epistasis data but may be readily applied to comparable imbalanced variable problems.

## Methods

### Data set generation

All data sets were generated using GAMETES, a tool that produces epistatic models between SNPs and creates data sets based off these models [27]. Penetrance functions were generated for SNP-SNP interaction scenarios for all 15 combinations of minor allele frequencies (MAFs) of {0.5, 0.4, 0.3, 0.2, and 0.1}, with SNP heritability kept constant at 0.005 and population prevalences of 0.5, 0.1, and 0.02 (Table 1, Additional file 1: Tables S1 and S2). Although a prevalence of 0.5 may seem high for a given disease, numerous risk factors for chronic and complex diseases in the United States population that may be phenotypes of interest are as or more prevalent, including being overweight or obese, lack of physical activity, excessive sodium consumption, lack of fruit and vegetable consumption, etc. [28]. The simulated data with prevalence of 0.1 is intended to reflect the US prevalence of common complex diseases such as diabetes or cardiovascular disease [29]. The simulated data sets of 0.02 prevalence approximately reflect the US prevalence of primary open-angle glaucoma, which is investigated in the real data application [30]. Balanced case-control ratio data sets of size 2000 and 10,000 were generated for the 0.5 prevalence scenario and of size 10,000 for the 0.1 and 0.02 prevalence scenarios.

**Table 1:** Data set simulation parameters, prevalence = 0.5

	Scenario 1			Scenario 2			Scenario 3		
SNP1 MAF:	0.1			0.2			0.2		
SNP2 MAF:	0.1			0.1			0.2		
Penetrance:	0.493	0.531	0.522	0.507	0.480	0.556	0.514	0.481	0.425
	0.526	0.387	0.410	0.471	0.590	0.249	0.467	0.544	0.674
	0.611	0.008	0.358	0.485	0.532	0.482	0.539	0.447	0.304
	Scenario 4			Scenario 5			Scenario 6		
SNP1 MAF:	0.3			0.3			0.3		
SNP2 MAF:	0.1			0.2			0.3		
Penetrance:	0.513	0.494	0.456	0.488	0.525	0.450	0.481	0.533	0.446
	0.438	0.530	0.696	0.527	0.455	0.562	0.525	0.468	0.513
	0.520	0.475	0.506	0.478	0.458	0.814	0.483	0.470	0.734
	Scenario 7			Scenario 8			Scenario 9		
SNP1 MAF:	0.4			0.4			0.4		
SNP2 MAF:	0.1			0.2			0.3		
Penetrance:	0.484	0.501	0.535	0.490	0.523	0.455	0.502	0.523	0.425
	0.570	0.494	0.359	0.512	0.468	0.568	0.499	0.472	0.588
	0.545	0.551	0.245	0.565	0.395	0.668	0.495	0.503	0.501
	Scenario 10			Scenario 11			Scenario 12		
SNP1 MAF:	0.4			0.5			0.5		
SNP2 MAF:	0.4			0.1			0.2		
Penetrance:	0.476	0.535	0.449	0.306	0.333	0.341	0.476	0.521	0.482
	0.506	0.473	0.568	0.428	0.314	0.256	0.521	0.472	0.536
	0.536	0.503	0.410	0.322	0.198	0.595	0.715	0.392	0.502
	Scenario 13			Scenario 14			Scenario 15		
SNP1 MAF:	0.5			0.5			0.5		
SNP2 MAF:	0.3			0.4			0.5		
Penetrance:	0.500	0.520	0.459	0.422	0.515	0.547	0.440	0.560	0.440
	0.477	0.480	0.563	0.548	0.491	0.470	0.522	0.484	0.509
	0.608	0.482	0.429	0.531	0.492	0.485	0.515	0.472	0.542

Minor allele frequencies and penetrance tables used to generate balanced case-control ratio data sets of size 2000 and 10,000. Heritability = 0.005 and prevalence = 0.5 constant across all simulations.

### Implementation and evaluation of traditional cross validation

For each of the 15 scenarios for each investigated prevalence and sample size combination, we perform 1000 replicates of a standard cross validation procedure in which two-thirds of observations are randomly allocated to be used for training and the remaining third is used for testing. The training data is then used to fit the following logistic regression models with and without the SNP-SNP interaction:

1 $$ P(case)=\frac{1}{1+{e}^{-\left({\beta}_0+{\beta}_1 SNP1+{\beta}_2 SNP2\right)}} $$

2 $$ P(case)=\frac{1}{1+{e}^{-\left({\beta}_0+{\beta}_1 SNP1+{\beta}_2 SNP2+{\beta}_3 SNP1\ast SNP2\right)}} $$

Where P(case) is a binary indicator of case-control status, SNP1 and SNP2 are categorical variables in which 0 corresponds to the homozygous dominant genotype, 1 to the heterozygous, and 2 to the homozygous recessive, and SNP1*SNP2 corresponds to the Cartesian product of the two {00, 01, 02, 10, 11, 12, 20, 21, 22}.

These models fit to the training data are then used to predict case-control status for the held-out testing data, using a cutoff of 0.5 for case versus control prediction assignment from the fitted values. These predictions are then used to calculate the sensitivity, specificity, positive predictive value, and negative predictive value for the testing data.

### Implementation and evaluation of proportional instance cross validation (PICV)

**Fig. 1:**
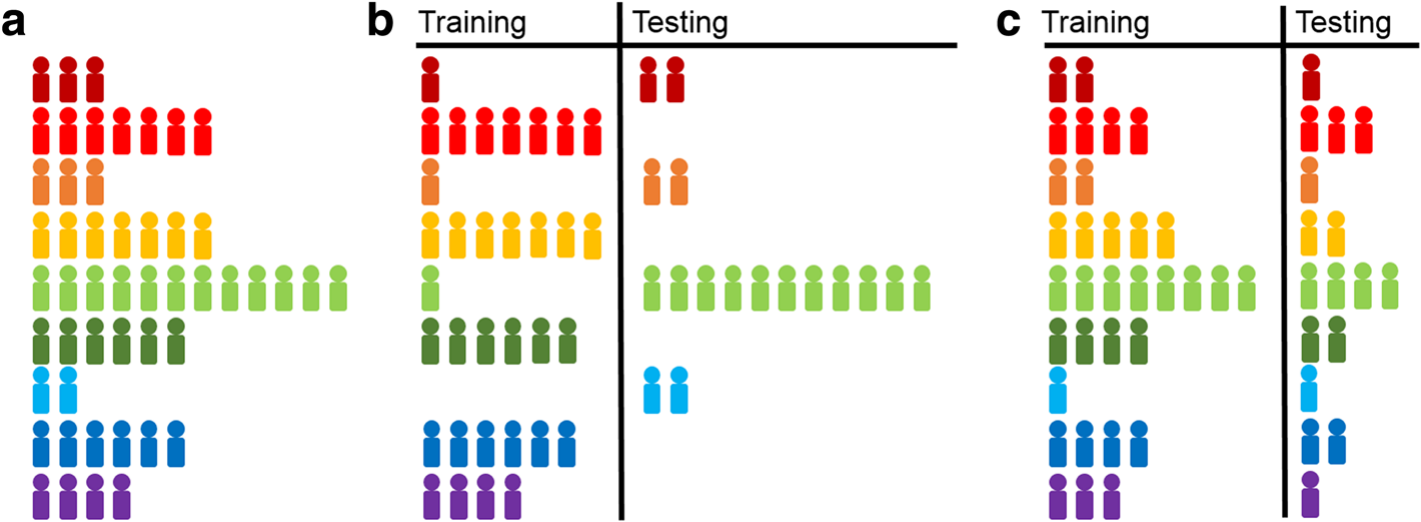
Comparing traditional cross validation and proportional instance cross validation (PICV). **a** The overall distribution of 9 SNP-SNP interaction genotypes (the 9 categories that result from the interaction of two SNPs in a hypothetical population of individuals. Note: only one possible allocation is depicted. **b** Traditional cross validation in which 2/3 of observations are randomly allocated to the training set and the remaining 1/3 are allocated to the testing set can result in draws with imbalanced genotype proportions. **c** PICV randomly allocates 2/3 of observations of each genotype to the training set and the remaining 1/3 to the testing set, ensuring that the relative proportions of genotypes are maintained

For the proportional instance cross validation procedure, rather than randomly allocating each observation to be included in the training or testing set, observations are allocated in a genotype-specific fashion (Fig. 1). Two-thirds of the observations of each SNP-SNP genotype are randomly allocated to be used for training and the remaining third is used for testing. Therefore the same total proportion of individuals used for training versus testing is maintained as in the traditional cross validation procedure, and additionally, the relative proportions of each genotype are preserved between the overall data set and the training and testing partitions. Model fitting with the training data, testing data predictions, and performance measure calculations are conducted as for the traditional cross validation.

### Comparison of traditional cross validation and proportional instance cross validation (PICV)

For both traditional cross validation and PICV, we calculate the absolute value of the difference between training and testing for each of four performance measures (sensitivity, specificity, positive predictive value, and negative predictive value) over 1000 trials for each of the 15 scenarios. We calculate *p*-values for the two-sample Kolmogorov-Smirnov test with the null hypothesis that there is no difference between the traditional cross validation implementation and PICV distributions of the difference between training and testing for each performance measure, with the one-sided alternative that the PICV distribution is smaller, with a significance threshold of α = 0.05.

## Results

**Fig. 2:**
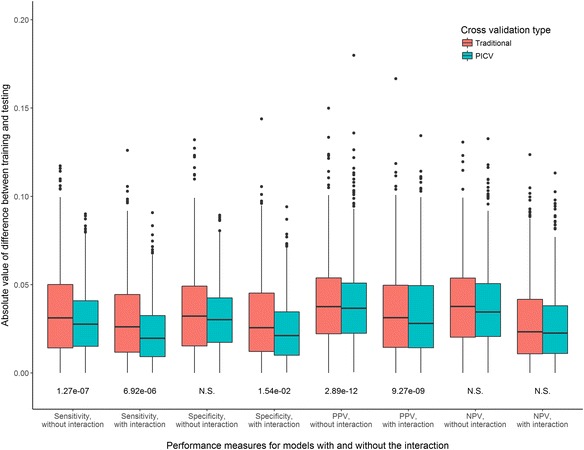
Consistency of training and testing performance measures for models with and without the interaction term, comparing a traditional cross validation procedure to PICV. Experimental scenario in which both SNPs have a MAF of 0.5, *n* = 2000. PPV: positive predictive value, NPV: negative predictive value, N.S.: not significant

Implementing PICV for our simulated epistasis examples (that is, performing cross validation data set splitting such that observations are allocated to maintain the same relative proportions of each SNP-SNP genotype in the training and testing sets as in the data set overall) significantly improved the consistency between training and testing sensitivities and positive predictive values. Consistency between the training and testing data set performance measures is of interest as the PICV method addresses the discordance between training and testing partitions that can occur in traditional cross validation. Fig. 2 illustrates comparisons of training/testing consistencies for PICV versus a traditional cross validation procedure in which observations are allocated to the training and testing sets without regard to genotype (see Additional file 1: Figures S1-S60 for all minor allele frequency, prevalence, and cohort size combinations). *P*-values listed are for the two-sample Kolmogorov-Smirnov test of the distributions of the absolute values of the differences between the training and testing performance measure (e.g. sensitivity) over 1000 trials per scenario for these two cross validation approaches, with a one-sided alternative hypothesis that the split-by-genotype distribution is smaller. Table 2 summarizes these performance measures across all 15 SNP-SNP genotype MAF combination scenarios for the 0.5 prevalence simulations of size 2000 (see Additional file 1: Table S3 for prevalence = 0.5 and *n* = 10,000, Additional file 1: Table S4 for prevalence = 0.1, Additional file 1: Table S5 for prevalence = 0.02). Sensitivity and positive predictive value were significantly more consistent between test and train for PICV than for traditional cross validation across all 15 scenarios tested for both *n* = 2000 and *n* = 10,000. Although the specificity and negative predictive value comparisons mostly did not meet statistical significance, smaller medians and maximum values for the differences in these performance measures between training and testing were observed for the PICV approach for the majority of scenarios (Table 3). These results demonstrate that PICV is comparable to traditional cross validation in terms of specificity and negative predictive value while providing advantages in sensitivity and positive predictive value.

**Table 2:** Summary of performance measures across minor allele frequency combinations, *n* = 2000

Measure, Model Scenario	Sensitivity, without interaction	Sensitivity, with interaction	Specificity, without interaction	Specificity, with interaction	PPV, without interaction	PPV, with interaction	NPV, without interaction	NPV, with interaction
SNP1 MAF: 0.1	3.06e-17	9.89e-08	N.S.	N.S.	1.90e-18	7.67e-08	N.S.	N.S.
SNP2 MAF: 0.1								
SNP1 MAF: 0.2	7.04e-20	4.54e-05	3.88e-02	N.S.	3.68e-11	5.56e-06	4.35e-02	1.89e-02
SNP2 MAF: 0.1								
SNP1 MAF: 0.2	1.69e-10	1.69e-10	N.S.	6.87e-03	4.06e-09	4.06e-09	N.S.	N.S.
SNP2 MAF: 0.2								
SNP1 MAF: 0.3	1.59e-08	2.47e-05	4.35e-02	N.S.	9.27e-09	2.47e-05	3.46e-02	N.S.
SNP2 MAF: 0.1								
SNP1 MAF: 0.3	6.14e-04	5.02e-11	N.S.	N.S.	3.07e-16	1.22e-14	N.S.	N.S.
SNP2 MAF: 0.2								
SNP1 MAF: 0.3	5.16e-04	4.33e-04	N.S.	N.S.	1.75e-04	1.75e-04	N.S.	N.S.
SNP2 MAF: 0.3								
SNP1 MAF: 0.4	9.94e-05	7.67e-08	N.S.	N.S.	3.52e-08	5.53e-10	N.S.	N.S.
SNP2 MAF: 0.1								
SNP1 MAF: 0.4	6.65e-17	1.45e-04	N.S.	N.S.	5.36e-09	2.42e-02	N.S.	N.S.
SNP2 MAF: 0.2								
SNP1 MAF: 0.4	2.71e-08	4.54e-05	N.S.	N.S.	8.97e-07	4.46e-06	N.S.	N.S.
SNP2 MAF: 0.3								
SNP1 MAF: 0.4	1.63e-05	1.41e-03	N.S.	N.S.	2.66e-03	8.62e-04	N.S.	N.S.
SNP2 MAF: 0.4								
SNP1 MAF: 0.5	8.97e-07	7.06e-09	N.S.	N.S.	2.27e-06	1.27e-07	4.85e-03	N.S.
SNP2 MAF: 0.1								
SNP1 MAF: 0.5	9.42e-18	6.75e-05	1.28e-02	N.S.	4.00e-12	8.60e-06	N.S.	N.S.
SNP2 MAF: 0.2								
SNP1 MAF: 0.5	4.38e-07	2.47e-05	N.S.	N.S.	7.67e-08	4.12e-10	N.S.	1.46e-02
SNP2 MAF: 0.3								
SNP1 MAF: 0.5	2.69e-07	5.54e-05	N.S.	N.S.	7.06e-09	8.62e-04	N.S.	N.S.
SNP2 MAF: 0.4								
SNP1 MAF: 0.5	1.27e-07	6.92e-06	N.S.	1.54e-02	2.89e-12	9.27e-09	N.S.	N.S.
SNP2 MAF: 0.5

**Table 3:** Number of scenarios for which PICV yielded smaller median, maximum differences between training and testing

Measure, Model	PICV median less than traditional CV median (out of 15)			PICV maximum less than traditional CV maximum (out of 15)		
	Prevalence			Prevalence		
	0.02	0.1	0.5	0.02	0.1	0.5
Specificity, without interaction	15	15	12	15	15	15
Specificity, with interaction	15	15	15	15	15	15
NPV, without interaction	14	9	9	11	8	8
NPV, with interaction	8	9	10	8	9	9

### Primary open-angle glaucoma interaction analysis

Prior interaction analyses of primary open-angle glaucoma identified several pairs of replicating interactions using the eMERGE and NEIGHBOR data [31]. We attempted to replicate the most significant interaction (between ALX4 and RBFOX1) in the GLAUGEN data set (dbGaP Study Accession: phs000308.v1.p1, available at https://www.ncbi.nlm.nih.gov/gap), which is harmonized with NEIGHBOR. The GLAUGEN model is adjusted for age, sex, site, and the first 6 principal components to reflect the eMERGE and NEIGHBOR models (the eMERGE and NEIGHBOR models additionally adjusted for platform, but all GLAUGEN samples were assessed on the same platform). Our analysis did not find a significant interaction between the two variants (Table 4). However, application of PICV to this data did yield training and testing *p*-values (0.376 and 0.323, respectively) more consistent with the overall LRT *p*-value (0.327) than a traditional cross validation procedure (0.442 and 0.470, respectively).

**Table 4:** Interaction analysis summary

Data set	ALX4 variant	RBFOX1 variant	LRT *p*-value
eMERGE	rs10838251	rs653127	7.29E-06
NEIGHBOR	rs7126447	rs11077011	1.62E-06
GLAUGEN	rs7126447	rs11077011	0.327

## Discussion

Implementing a cross validation splitting procedure that maintains the relative proportions of each SNP-SNP genotype when dividing the overall data set significantly improved the sensitivity and positive predictive value consistencies between the training and testing partitions in each of the experimental scenarios tested. Although specificity and negative predictive value improvement did not meet statistical significance in most cases, application of the PICV approach did yield smaller median and maximum absolute differences between training and testing in the majority of scenarios. The interaction analysis did not replicate the prior finding between ALX4 and RBFOX1, however PICV still produced more consistent estimates than a traditional cross validation procedure for this data. Verma et al. note that RBFOX1 has been previously shown to be associated with myopia, and that eMERGE primary open-angle glaucoma cases had not been screened for myopia; GLAUGEN excluded individuals with more than 8 diopters of myopia. This inconsistent finding highlights the importance of considering epidemiological confounders and co-morbidities of complex phenotypes in genetic analyses.

Class imbalance is a well-recognized issue in machine learning analyses, particularly for the analysis of high-dimensional data sets as in genomics and other biomedical applications [32]. If the main objective of a machine learning analysis is maximizing accuracy, and the minority class is very small, simply predicting the majority class for each observation may yield high overall accuracy, as in the spam filtering problem [33]. Clearly, adoption of a balanced accuracy measure or a cost-sensitivity analysis that weighs the relative importance of avoiding false positives versus false negatives is critical for such problems, and numerous methods have been developed to address this issue including novel fitness functions, sampling-based approaches, and ensemble methods, including for epistasis modeling [34–37]. The present study, though thematically similar to the class imbalance problem, instead addresses imbalance in observations of classes of an independent variable, e.g. the SNP-SNP interaction genotype. This is also adjacent to the covariate and data set shift problems, in which the training and testing distributions differ (for example due to model training using clean data from consistent laboratory conditions to produce models that then fail to hold for experimentally gathered data with unanticipated environmental differences), but for internal cross validation [38–40]. Solutions to problems of both of these genres include re-weighting and –sampling techniques, whereas the present study circumvents the need for either via splitting the data to ensure balanced proportions by genotype between training and testing sets. The example application of imbalanced SNP-SNP genotypes considers a categorical variable, but the underlying idea of preserving the distribution of instances between training and testing with regard to an independent variable could be extended to continuous variables or combinations of variables via binning, propensity scores, etc.

## Conclusions

Although the contribution of epistatic interactions may help explain the “missing heritability” of complex disease, statistical detection of epistasis remains challenging and can require adjustment of general machine learning protocols. With decreasing minor allele frequencies, the number of observations for rare SNP-SNP interaction genotypes becomes quite small in a GWAS of typical size, and a standard cross validation procedure may result in training/testing data set splits that poorly represent the data as a whole. This diminishes the ability to identify interactions of potential interest for experimental follow-up, and underscores the need to perform interaction analyses in an interaction-specific framework. A potentially overlooked element of performing reproducible analyses includes the imperative to develop and modify methods considering how intrinsic characteristics of the data and its structure may contribute to statistical failure to replicate despite biological (or other scientific) validity. Genomics and the biomedical sciences in general benefit from their increasingly multidisciplinary nature by incorporating methodology and theory from adjacent computational fields, but thoughtful contextualization of the data in view of the underlying biology is necessary to reap the potential benefits of applied machine learning methods and to successfully reproduce them.

## Additional file

Additional file 1: Supplemental tables and figures of data set simulation parameters and performance measures for all minor allele frequency, prevalence, and sample size combinations. (DOCX 3350 kb)
