# Supplementary material for: Improving machine learning reproducibility in genetic association studies with proportional instance cross validation (PICV)
Source: BioData Min. 2018 Apr 19;11:6. doi: 10.1186/s13040-018-0167-7 (PMC5907739; doi:10.1186/s13040-018-0167-7)
Supplement: Supplementary file 1 — Supplemental tables and figures of data set simulation parameters and performance measures for all minor allele frequency, prevalence, and sample size combinations. (DOCX 3350 kb) [file 13040_2018_167_MOESM1_ESM.docx]

**Additional file 1**

**Table S1.** Data set simulation parameters. Minor allele frequencies and penetrance tables used to generate balanced case-control ratio data sets of size 10,000. Heritability = 0.005 and prevalence = 0.1 constant across all simulations.

|  | **Scenario 1** | | | **Scenario 2** | | | **Scenario 3** | | |
| --- | --- | --- | --- | --- | --- | --- | --- | --- | --- |
| **SNP1 MAF:** | 0.1 | | | 0.2 | | | 0.2 | | |
| **SNP2 MAF:** | 0.1 | | | 0.1 | | | 0.2 | | |
| **Penetrance:** | 0.205 | 0.182 | 0.102 | 0.208 | 0.190 | 0.148 | 0.189 | 0.213 | 0.273 |
|  | 0.176 | 0.282 | 0.640 | 0.164 | 0.244 | 0.433 | 0.225 | 0.166 | 0.066 |
|  | 0.219 | 0.110 | 0.248 | 0.202 | 0.198 | 0.192 | 0.173 | 0.266 | 0.094 |
|  | **Scenario 4** | | | **Scenario 5** | | | **Scenario 6** | | |
| **SNP1 MAF:** | 0.3 | | | 0.3 | | | 0.3 | | |
| **SNP2 MAF:** | 0.1 | | | 0.2 | | | 0.3 | | |
| **Penetrance:** | 0.211 | 0.194 | 0.166 | 0.201 | 0.203 | 0.182 | 0.220 | 0.172 | 0.223 |
|  | 0.153 | 0.227 | 0.334 | 0.203 | 0.201 | 0.181 | 0.191 | 0.220 | 0.157 |
|  | 0.151 | 0.186 | 0.532 | 0.166 | 0.146 | 0.639 | 0.134 | 0.260 | 0.276 |
|  | **Scenario 7** | | | **Scenario 8** | | | **Scenario 9** | | |
| **SNP1 MAF:** | 0.4 | | | 0.4 | | | 0.4 | | |
| **SNP2 MAF:** | 0.1 | | | 0.2 | | | 0.3 | | |
| **Penetrance:** | 0.188 | 0.199 | 0.228 | 0.221 | 0.191 | 0.180 | 0.202 | 0.218 | 0.143 |
|  | 0.253 | 0.203 | 0.072 | 0.157 | 0.212 | 0.260 | 0.204 | 0.175 | 0.267 |
|  | 0.180 | 0.212 | 0.208 | 0.206 | 0.248 | 0.040 | 0.172 | 0.221 | 0.198 |
|  | **Scenario 10** | | | **Scenario 11** | | | **Scenario 12** | | |
| **SNP1 MAF:** | 0.4 | | | 0.5 | | | 0.5 | | |
| **SNP2 MAF:** | 0.4 | | | 0.1 | | | 0.2 | | |
| **Penetrance:** | 0.159 | 0.228 | 0.209 | 0.205 | 0.207 | 0.180 | 0.190 | 0.192 | 0.225 |
|  | 0.211 | 0.186 | 0.217 | 0.170 | 0.177 | 0.277 | 0.216 | 0.224 | 0.136 |
|  | 0.259 | 0.179 | 0.130 | 0.296 | 0.026 | 0.452 | 0.229 | 0.130 | 0.310 |
|  | **Scenario 13** | | | **Scenario 14** | | | **Scenario 15** | | |
| **SNP1 MAF:** | 0.5 | | | 0.5 | | | 0.5 | | |
| **SNP2 MAF:** | 0.3 | | | 0.4 | | | 0.5 | | |
| **Penetrance:** | 0.185 | 0.188 | 0.239 | 0.154 | 0.204 | 0.238 | 0.224 | 0.279 | 0.219 |
|  | 0.233 | 0.207 | 0.152 | 0.246 | 0.195 | 0.164 | 0.220 | 0.187 | 0.205 |
|  | 0.124 | 0.234 | 0.208 | 0.167 | 0.205 | 0.222 | 0.236 | 0.247 | 0.170 |

**Table S2.** Data set simulation parameters. Minor allele frequencies and penetrance tables used to generate balanced case-control ratio data sets of size 10,000. Heritability = 0.005 and prevalence = 0.02 constant across all simulations.

|  | **Scenario 1** | | | **Scenario 2** | | | **Scenario 3** | | |
| --- | --- | --- | --- | --- | --- | --- | --- | --- | --- |
| **SNP1 MAF:** | 0.1 | | | 0.2 | | | 0.2 | | |
| **SNP2 MAF:** | 0.1 | | | 0.1 | | | 0.2 | | |
| **Penetrance:** | 0.022 | 0.013 | 0.008 | 0.023 | 0.016 | 0.002 | 0.016 | 0.027 | 0.019 |
|  | 0.013 | 0.052 | 0.035 | 0.007 | 0.036 | 0.010 | 0.028 | 0.006 | 0.006 |
|  | 0.016 | 0.008 | 0.709 | 0.022 | 0.016 | 0.029 | 0.012 | 0.016 | 0.183 |
|  | **Scenario 4** | | | **Scenario 5** | | | **Scenario 6** | | |
| **SNP1 MAF:** | 0.3 | | | 0.3 | | | 0.3 | | |
| **SNP2 MAF:** | 0.1 | | | 0.2 | | | 0.3 | | |
| **Penetrance:** | 0.024 | 0.017 | 0.009 | 0.017 | 0.027 | 0.002 | 0.017 | 0.027 | 0.003 |
|  | 0.001 | 0.031 | 0.069 | 0.025 | 0.007 | 0.056 | 0.027 | 0.008 | 0.039 |
|  | 0.022 | 0.021 | 0.005 | 0.023 | 0.017 | 0.016 | 0.003 | 0.039 | 0.023 |
|  | **Scenario 7** | | | **Scenario 8** | | | **Scenario 9** | | |
| **SNP1 MAF:** | 0.4 | | | 0.4 | | | 0.4 | | |
| **SNP2 MAF:** | 0.1 | | | 0.2 | | | 0.3 | | |
| **Penetrance:** | 0.018 | 0.024 | 0.011 | 0.019 | 0.025 | 0.006 | 0.031 | 0.012 | 0.009 |
|  | 0.028 | 0.009 | 0.058 | 0.025 | 0.008 | 0.047 | 0.010 | 0.028 | 0.023 |
|  | 0.026 | 0.010 | 0.034 | 0.003 | 0.031 | 0.023 | 0.010 | 0.012 | 0.067 |
|  | **Scenario 10** | | | **Scenario 11** | | | **Scenario 12** | | |
| **SNP1 MAF:** | 0.4 | | | 0.5 | | | 0.5 | | |
| **SNP2 MAF:** | 0.4 | | | 0.1 | | | 0.2 | | |
| **Penetrance:** | 0.005 | 0.028 | 0.029 | 0.022 | 0.023 | 0.012 | 0.025 | 0.024 | 0.008 |
|  | 0.027 | 0.019 | 0.007 | 0.012 | 0.006 | 0.056 | 0.010 | 0.013 | 0.045 |
|  | 0.031 | 0.005 | 0.039 | 0.023 | 0.014 | 0.030 | 0.025 | 0.020 | 0.017 |
|  | **Scenario 13** | | | **Scenario 14** | | | **Scenario 15** | | |
| **SNP1 MAF:** | 0.5 | | | 0.5 | | | 0.5 | | |
| **SNP2 MAF:** | 0.3 | | | 0.4 | | | 0.5 | | |
| **Penetrance:** | 0.006 | 0.023 | 0.029 | 0.041 | 0.012 | 0.014 | 0.045 | 0.004 | 0.026 |
|  | 0.036 | 0.019 | 0.006 | 0.010 | 0.026 | 0.019 | 0.013 | 0.026 | 0.016 |
|  | 0.022 | 0.010 | 0.040 | 0.004 | 0.020 | 0.036 | 0.009 | 0.025 | 0.021 |

**Table S3.** Summary of performance measures across minor allele frequency combinations, prevalence = 0.5, n = 10000.

| Measure,  Model  Scenario | Sens, without int | Sens, with int | Spec, without int | Spec, with int | PPV, without int | PPV,  with int | NPV, without int | NPV,  with  int |
| --- | --- | --- | --- | --- | --- | --- | --- | --- |
| SNP1 MAF: 0.1  SNP2 MAF: 0.1 | 8.60e-06 | 1.75e-04 | N.S. | N.S. | 4.12e-10 | 4.06e-09 | N.S. | N.S. |
| SNP1 MAF: 0.2  SNP2 MAF: 0.1 | 6.92e-06 | 2.47e-05 | N.S. | 2.68e-02 | 3.03e-20 | 9.55e-22 | 4.35e-02 | N.S. |
| SNP1 MAF: 0.2  SNP2 MAF: 0.2 | 9.27e-09 | 3.03e-05 | N.S. | N.S. | 5.01e-14 | 5.92e-15 | 2.73e-02 | N.S. |
| SNP1 MAF: 0.3  SNP2 MAF: 0.1 | 2.27e-06 | 1.20e-03 | N.S. | N.S. | 1.74e-14 | 2.81e-13 | N.S. | N.S. |
| SNP1 MAF: 0.3  SNP2 MAF: 0.2 | 6.65e-17 | 4.12e-10 | N.S. | N.S. | 1.48e-21 | 1.07e-19 | N.S. | 1.46e-02 |
| SNP1 MAF: 0.3  SNP2 MAF: 0.3 | 5.56e-06 | 2.10e-07 | N.S. | N.S. | 1.75e-09 | 3.07e-16 | N.S. | N.S. |
| SNP1 MAF: 0.4  SNP2 MAF: 0.1 | 8.97e-07 | 4.54e-05 | N.S. | N.S. | 7.73e-13 | 1.00e-13 | N.S. | N.S. |
| SNP1 MAF: 0.4  SNP2 MAF: 0.2 | 2.66e-03 | 5.54e-05 | N.S. | N.S. | 2.86e-15 | 2.07e-17 | N.S. | N.S. |
| SNP1 MAF: 0.4  SNP2 MAF: 0.3 | 4.38e-07 | 4.06e-09 | N.S. | N.S. | 1.44e-11 | 1.75e-09 | N.S. | N.S. |
| SNP1 MAF: 0.4  SNP2 MAF: 0.4 | 3.63e-04 | 2.11e-04 | N.S. | N.S. | 1.97e-11 | 1.63e-05 | N.S. | N.S. |
| SNP1 MAF: 0.5  SNP2 MAF: 0.1 | 5.57e-07 | 7.08e-07 | N.S. | N.S. | 1.43e-16 | 6.65e-17 | N.S. | 7.45e-03 |
| SNP1 MAF: 0.5  SNP2 MAF: 0.2 | 4.06e-09 | 5.93e-08 | N.S. | N.S. | 3.95e-22 | 1.62e-19 | N.S. | N.S. |
| SNP1 MAF: 0.5  SNP2 MAF: 0.3 | 1.27e-07 | 2.32e-09 | N.S. | N.S. | 4.00e-12 | 1.69e-10 | N.S. | N.S. |
| SNP1 MAF: 0.5  SNP2 MAF: 0.4 | 8.97e-07 | 1.13e-06 | N.S. | N.S. | 1.43e-16 | 1.37e-15 | 3.08e-02 | N.S. |
| SNP1 MAF: 0.5  SNP2 MAF: 0.5 | 4.06e-09 | 3.71e-05 | N.S. | N.S. | 5.53e-13 | 2.89e-12 | N.S. | N.S. |

**Table S4.** Summary of performance measures across minor allele frequency combinations, prevalence = 0.1, n = 10000.

| Measure,  Model  Scenario | Sens, without int | Sens, with int | Spec, without int | Spec, with int | PPV, without int | PPV,  with int | NPV, without int | NPV,  with  int |
| --- | --- | --- | --- | --- | --- | --- | --- | --- |
| SNP1 MAF: 0.1  SNP2 MAF: 0.1 | 6.75e-05 | 2.10e-07 | N.S. | N.S. | 1.74e-14 | 1.97e-11 | N.S. | N.S. |
| SNP1 MAF: 0.2  SNP2 MAF: 0.1 | 2.08e-08 | 1.22e-08 | 4.86e-02 | N.S. | 1.97e-11 | 6.50e-16 | N.S. | N.S. |
| SNP1 MAF: 0.2  SNP2 MAF: 0.2 | 1.07e-05 | 1.32e-05 | N.S. | N.S. | 1.97e-11 | 3.07e-16 | N.S. | N.S. |
| SNP1 MAF: 0.3  SNP2 MAF: 0.1 | 3.07e-09 | 1.74e-14 | N.S. | N.S. | 3.07e-16 | 1.40e-17 | N.S. | N.S. |
| SNP1 MAF: 0.3  SNP2 MAF: 0.2 | 5.57e-07 | 5.53e-10 | N.S. | N.S. | 4.47e-16 | 1.22e-14 | N.S. | 4.35e-02 |
| SNP1 MAF: 0.3  SNP2 MAF: 0.3 | 1.07e-05 | 8.20e-05 | 5.21e-03 | N.S. | 6.15e-22 | 9.25e-11 | N.S. | N.S. |
| SNP1 MAF: 0.4  SNP2 MAF: 0.1 | 2.71e-08 | 2.47e-05 | N.S. | N.S. | 1.22e-14 | 1.37e-15 | N.S. | N.S. |
| SNP1 MAF: 0.4  SNP2 MAF: 0.2 | 8.60e-06 | 1.07e-05 | N.S. | N.S. | 5.92e-15 | 5.53e-13 | N.S. | 3.88e-02 |
| SNP1 MAF: 0.4  SNP2 MAF: 0.3 | 9.87e-10 | 9.87e-10 | N.S. | N.S. | 5.53e-13 | 6.33e-18 | N.S. | N.S. |
| SNP1 MAF: 0.4  SNP2 MAF: 0.4 | 3.71e-05 | 5.53e-10 | N.S. | N.S. | 5.02e-11 | 9.42e-18 | N.S. | N.S. |
| SNP1 MAF: 0.5  SNP2 MAF: 0.1 | 9.25e-11 | 5.53e-10 | N.S. | N.S. | 7.73e-13 | 4.52e-17 | N.S. | N.S. |
| SNP1 MAF: 0.5  SNP2 MAF: 0.2 | 5.54e-05 | 2.27e-06 | N.S. | N.S. | 1.22e-14 | 3.03e-20 | 1.46e-02 | N.S. |
| SNP1 MAF: 0.5  SNP2 MAF: 0.3 | 1.32e-05 | 3.07e-10 | N.S. | N.S. | 2.27e-06 | 1.22e-08 | N.S. | N.S. |
| SNP1 MAF: 0.5  SNP2 MAF: 0.4 | 6.75e-05 | 5.57e-07 | N.S. | N.S. | 9.25e-11 | 2.08e-08 | N.S. | N.S. |
| SNP1 MAF: 0.5  SNP2 MAF: 0.5 | 1.22e-14 | 5.53e-12 | N.S. | N.S. | 6.50e-16 | 7.10e-14 | N.S. | N.S. |

**Table S5.** Summary of performance measures across minor allele frequency combinations, prevalence = 0.02, n = 10000.

| Measure,  Model  Scenario | Sens, without int | Sens, with int | Spec, without int | Spec, with int | PPV, without int | PPV,  with int | NPV, without int | NPV,  with  int |
| --- | --- | --- | --- | --- | --- | --- | --- | --- |
| SNP1 MAF: 0.1  SNP2 MAF: 0.1 | 2.28e-10 | 9.25e-11 | 4.20e-03 | 1.12e-02 | 1.40e-17 | 1.98e-20 | 1.46e-02 | 1.46e-02 |
| SNP1 MAF: 0.2  SNP2 MAF: 0.1 | 1.80e-06 | 1.75e-09 | 1.03e-02 | N.S. | 1.25e-10 | 4.21e-24 | N.S. | N.S. |
| SNP1 MAF: 0.2  SNP2 MAF: 0.2 | 7.10e-14 | 1.05e-11 | N.S. | 1.70e-02 | 9.27e-09 | 3.72e-19 | N.S. | N.S. |
| SNP1 MAF: 0.3  SNP2 MAF: 0.1 | 3.07e-09 | 9.27e-09 | N.S. | 5.58e-03 | 9.25e-11 | 1.98e-20 | N.S. | 4.35e-02 |
| SNP1 MAF: 0.3  SNP2 MAF: 0.2 | 1.13e-06 | 3.07e-09 | 4.09e-03 | N.S. | 2.32e-09 | 1.66e-24 | N.S. | N.S. |
| SNP1 MAF: 0.3  SNP2 MAF: 0.3 | 1.32e-09 | 5.53e-12 | 3.16e-02 | N.S. | 1.25e-10 | 2.10e-16 | N.S. | N.S. |
| SNP1 MAF: 0.4  SNP2 MAF: 0.1 | 1.08e-12 | 4.12e-10 | N.S. | 3.73e-02 | 3.52e-08 | 3.19e-27 | N.S. | N.S. |
| SNP1 MAF: 0.4  SNP2 MAF: 0.2 | 4.12e-10 | 7.40e-10 | 1.80e-02 | N.S. | 8.49e-15 | 1.98e-15 | N.S. | N.S. |
| SNP1 MAF: 0.4  SNP2 MAF: 0.3 | 7.73e-13 | 1.43e-16 | 1.07e-02 | 4.93e-03 | 7.67e-08 | 3.95e-22 | N.S. | N.S. |
| SNP1 MAF: 0.4  SNP2 MAF: 0.4 | 5.46e-21 | 1.08e-12 | N.S. | N.S. | 2.89e-12 | 1.22e-14 | N.S. | N.S. |
| SNP1 MAF: 0.5  SNP2 MAF: 0.1 | 5.36e-09 | 6.14e-04 | N.S. | N.S. | 6.82e-11 | 1.97e-11 | N.S. | N.S. |
| SNP1 MAF: 0.5  SNP2 MAF: 0.2 | 4.38e-07 | 4.06e-09 | N.S. | N.S. | 2.69e-07 | 4.52e-17 | N.S. | N.S. |
| SNP1 MAF: 0.5  SNP2 MAF: 0.3 | 7.06e-09 | 4.58e-08 | N.S. | N.S. | 7.73e-13 | 8.44e-19 | N.S. | N.S. |
| SNP1 MAF: 0.5  SNP2 MAF: 0.4 | 1.43e-06 | 5.36e-09 | N.S. | N.S. | 1.44e-11 | 5.46e-21 | 4.35e-02 | N.S. |
| SNP1 MAF: 0.5  SNP2 MAF: 0.5 | 5.02e-11 | 5.57e-07 | N.S. | 2.75e-05 | 3.44e-07 | 1.62e-19 | N.S. | N.S. |

**
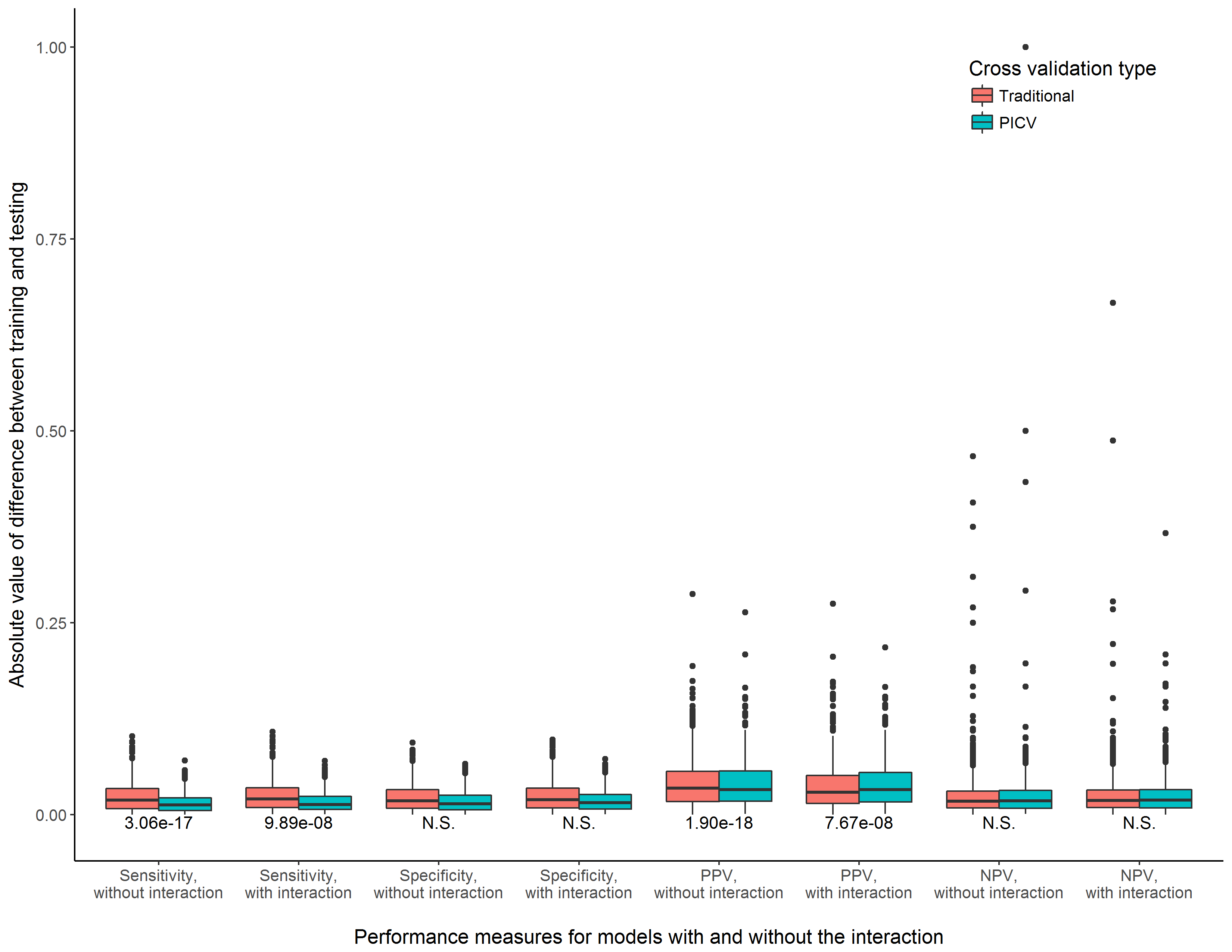
**

**Figure S1.** Consistency of training and testing performance measures for models with and without the interaction term, comparing a traditional cross validation procedure to PICV. Experimental scenario 1, prevalence = 0.5, n = 2000


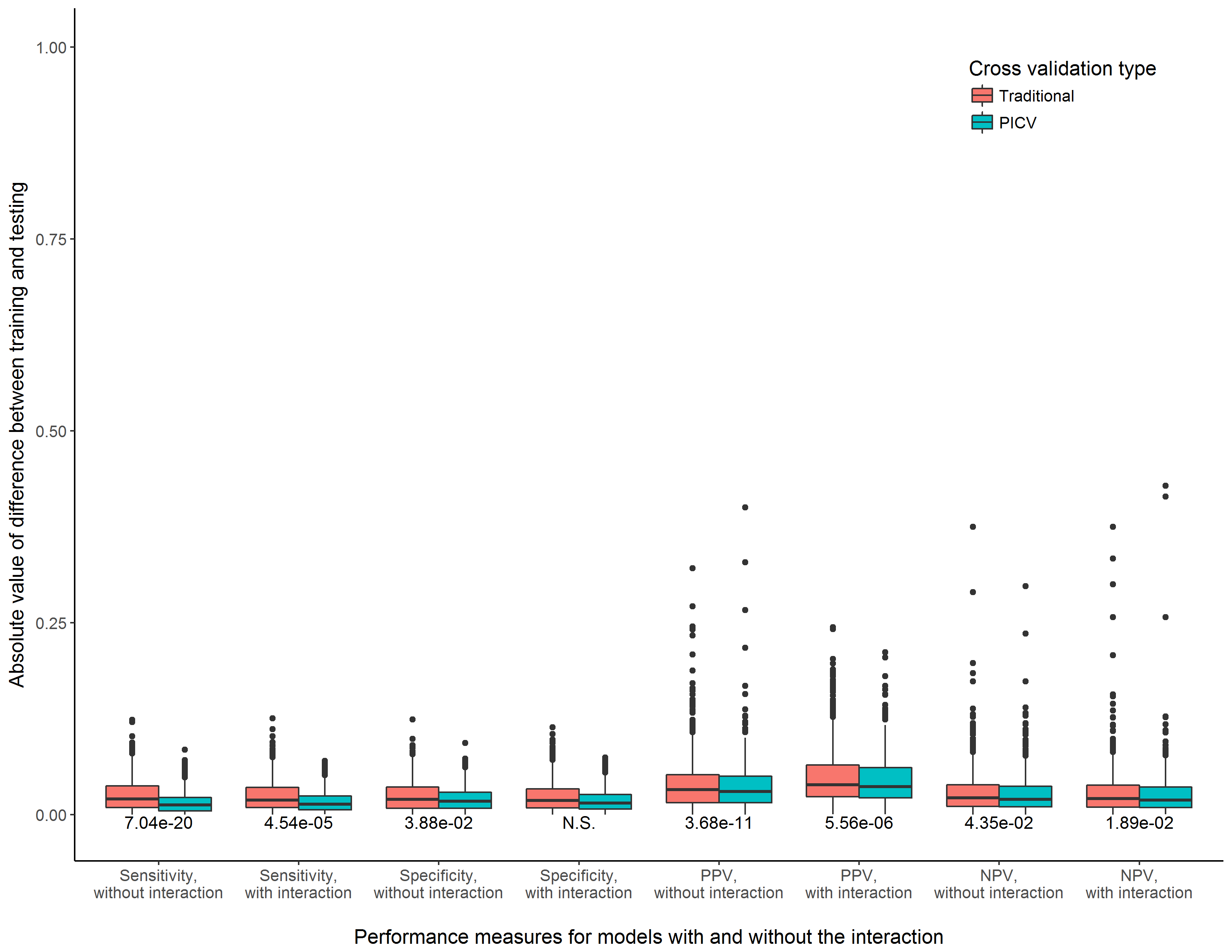


**Figure S2.** Consistency of training and testing performance measures for models with and without the interaction term, comparing a traditional cross validation procedure to PICV. Experimental scenario 2, prevalence = 0.5, n = 2000


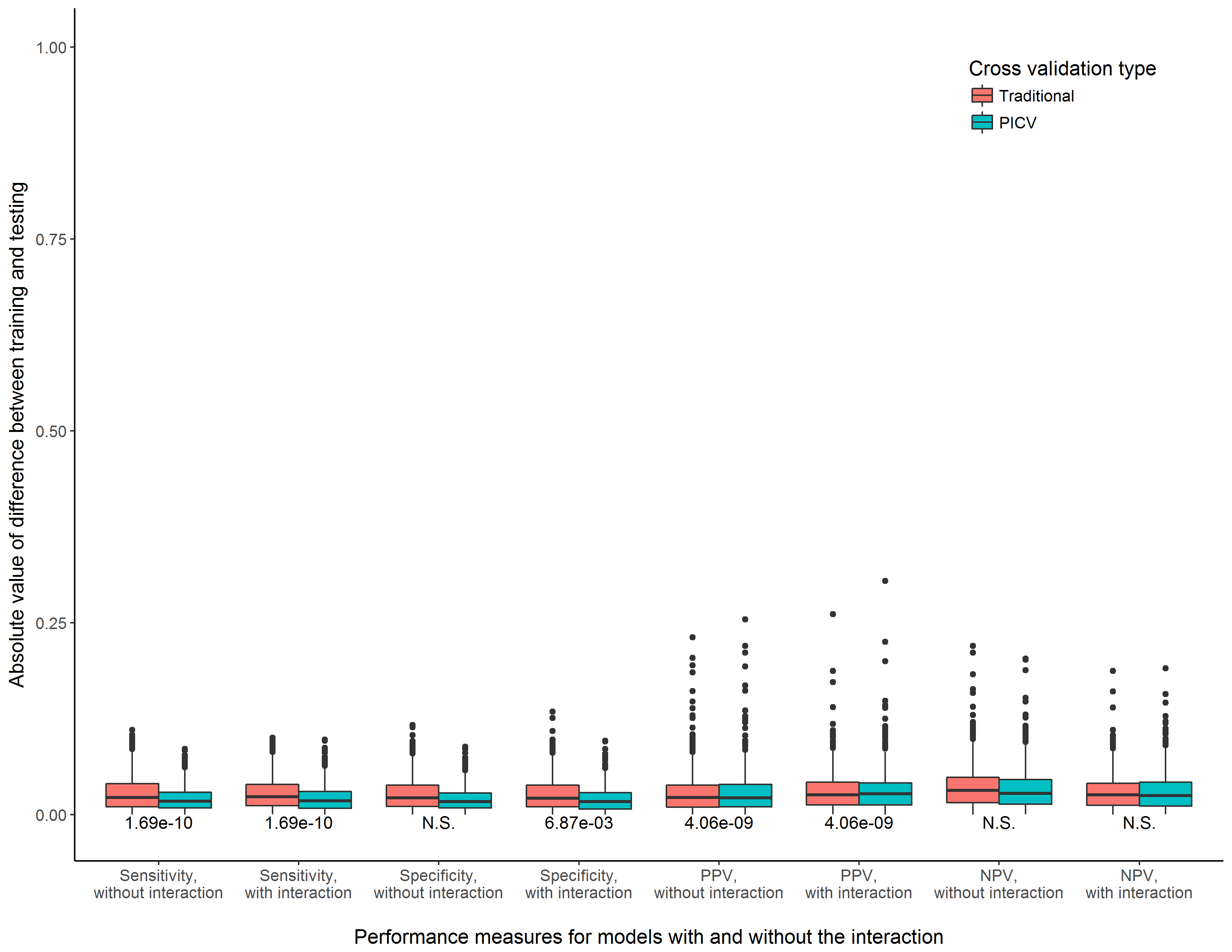


**Figure S3.** Consistency of training and testing performance measures for models with and without the interaction term, comparing a traditional cross validation procedure to PICV. Experimental scenario 3, prevalence = 0.5, n = 2000

**
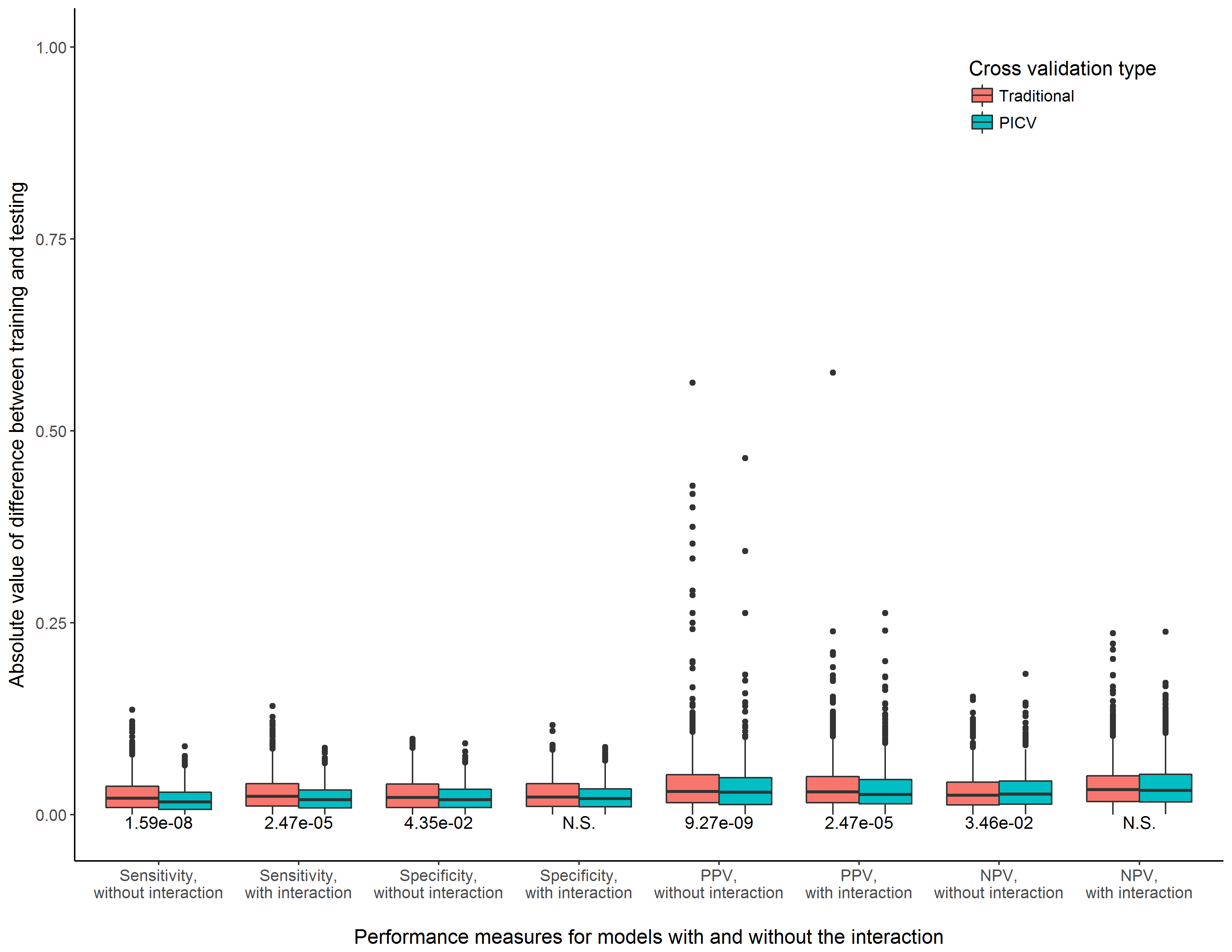
**

**Figure S4.** Consistency of training and testing performance measures for models with and without the interaction term, comparing a traditional cross validation procedure to PICV. Experimental scenario 4, prevalence = 0.5, n = 2000


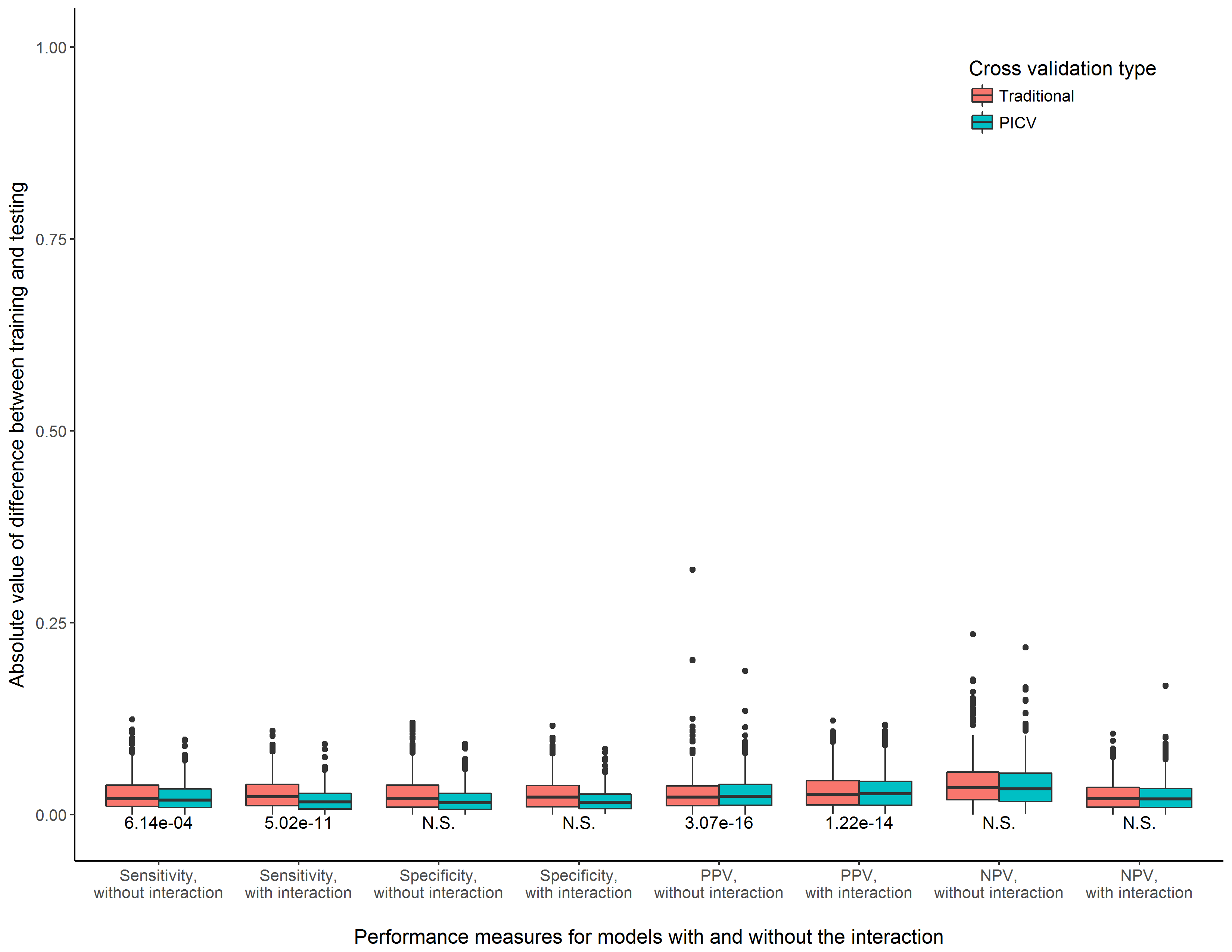


**Figure S5.** Consistency of training and testing performance measures for models with and without the interaction term, comparing a traditional cross validation procedure to PICV. Experimental scenario 5, prevalence = 0.5, n = 2000


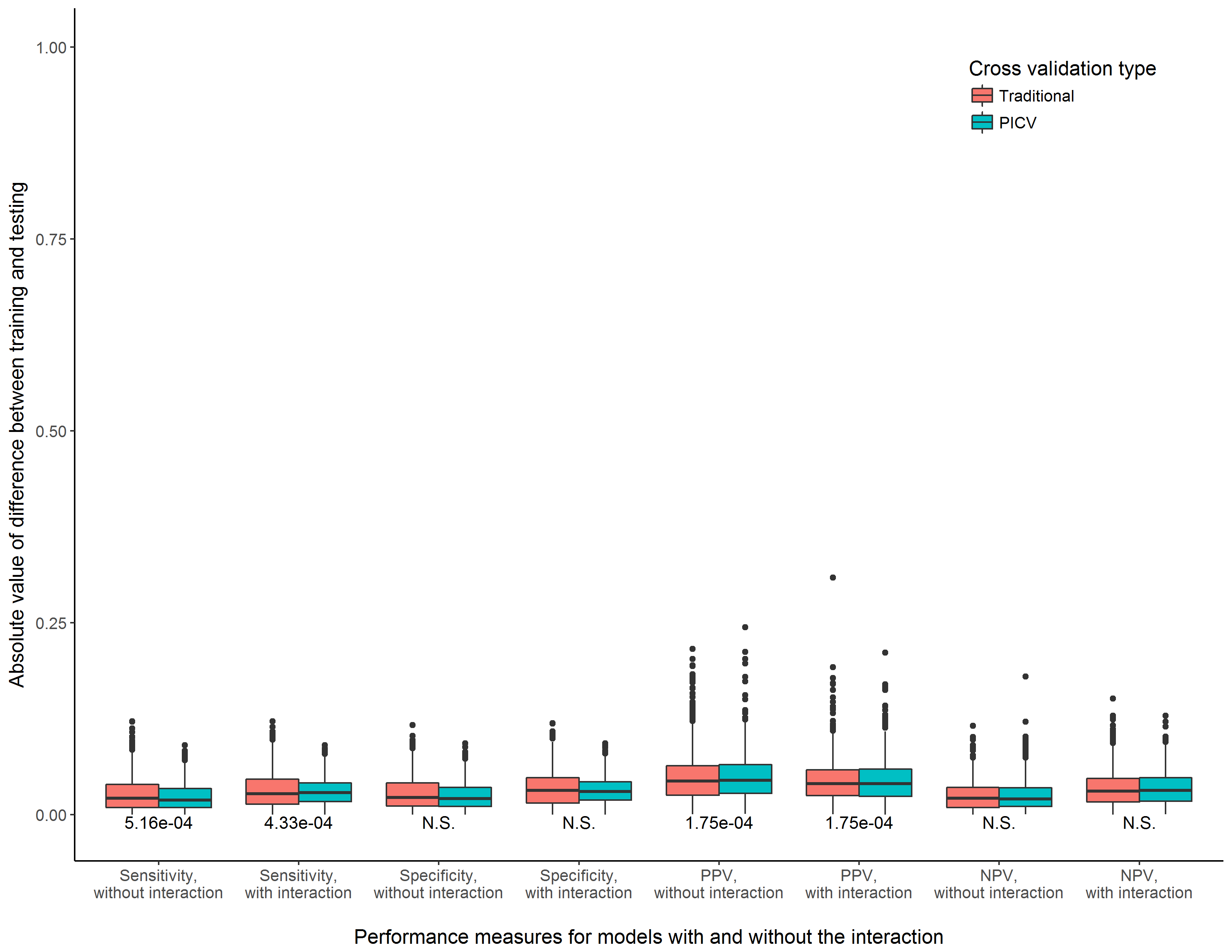


**Figure S6.** Consistency of training and testing performance measures for models with and without the interaction term, comparing a traditional cross validation procedure to PICV. Experimental scenario 6, prevalence = 0.5, n = 2000


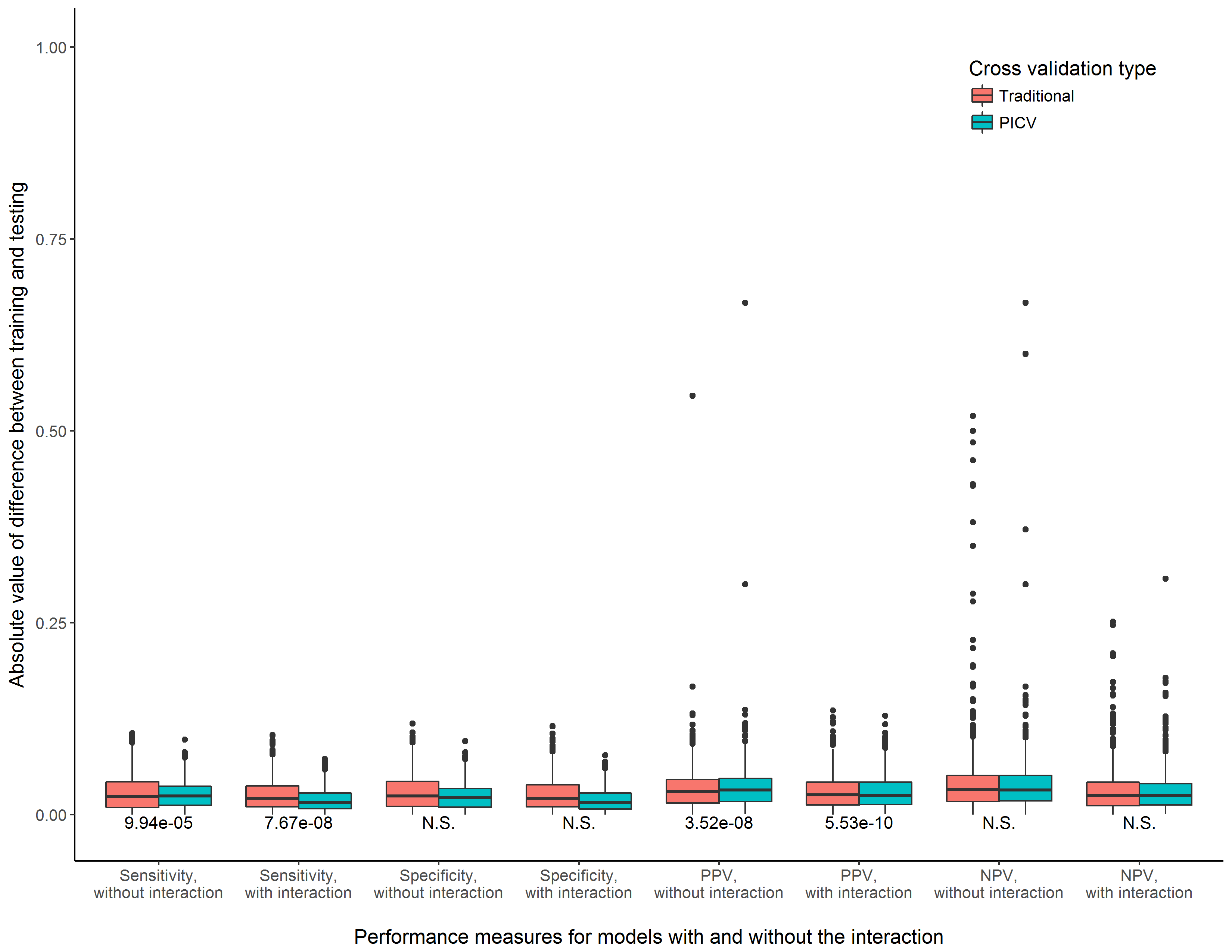


**Figure S7.** Consistency of training and testing performance measures for models with and without the interaction term, comparing a traditional cross validation procedure to PICV. Experimental scenario 7, prevalence = 0.5, n = 2000


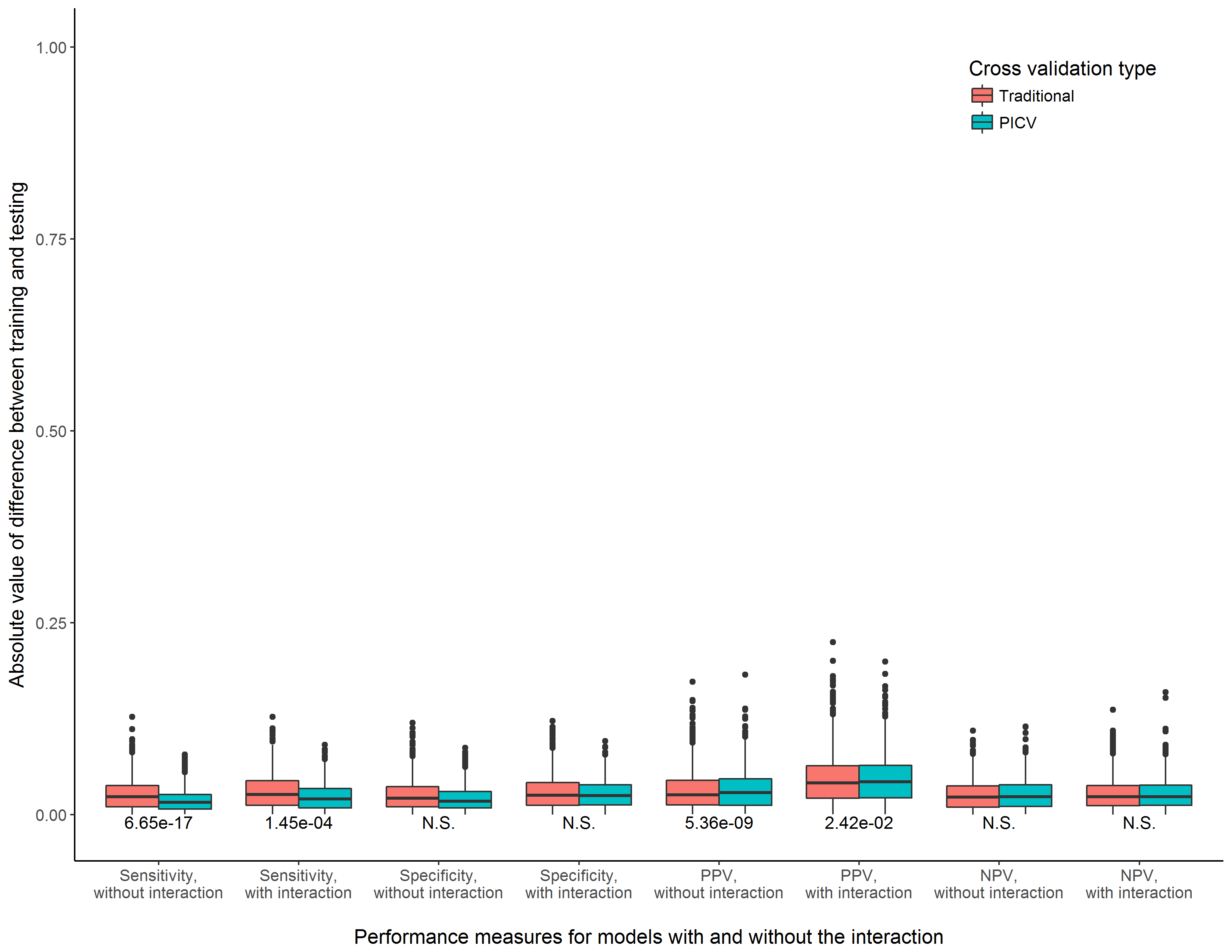


**Figure S8.** Consistency of training and testing performance measures for models with and without the interaction term, comparing a traditional cross validation procedure to PICV. Experimental scenario 8, prevalence = 0.5, n = 2000


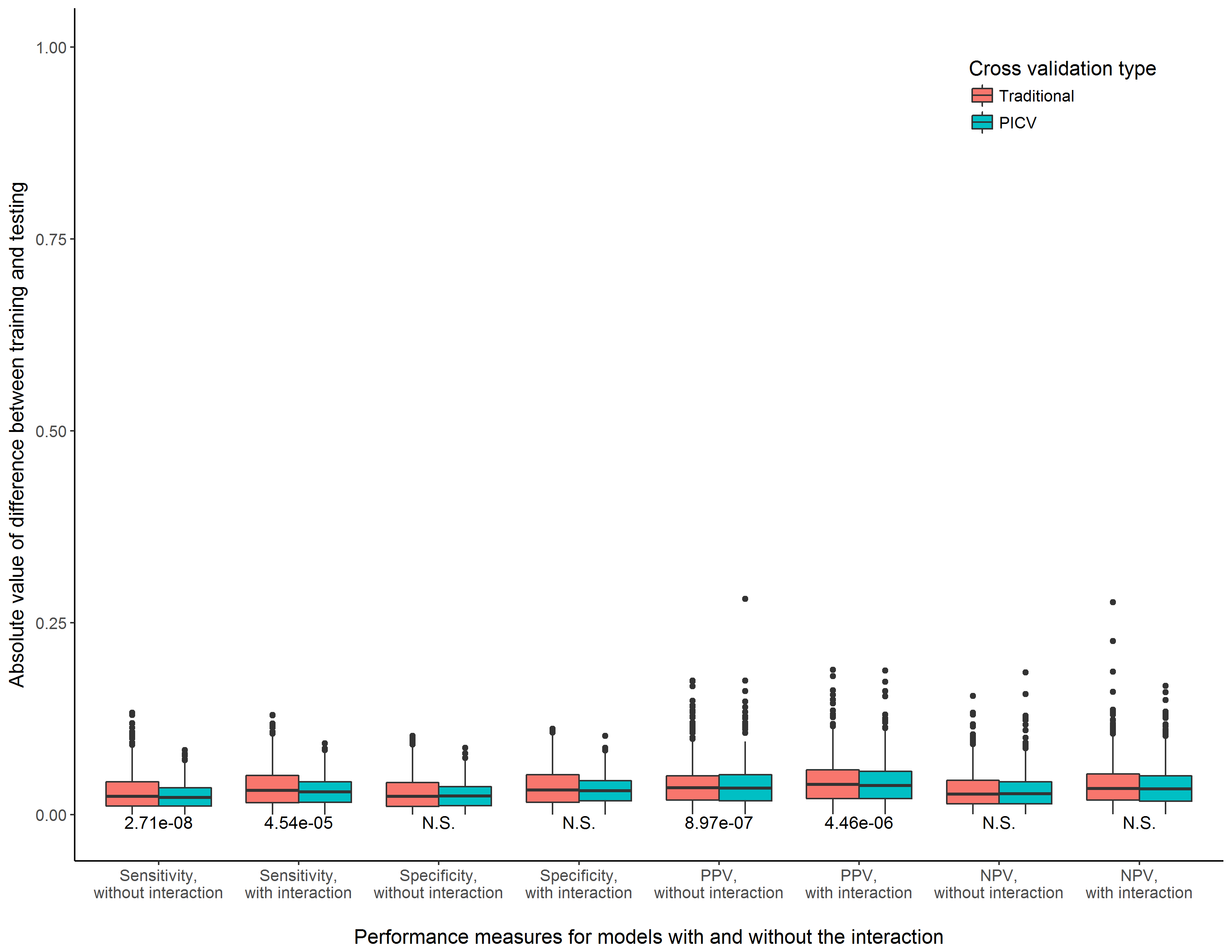


**Figure S9.** Consistency of training and testing performance measures for models with and without the interaction term, comparing a traditional cross validation procedure to PICV. Experimental scenario 9, prevalence = 0.5, n = 2000


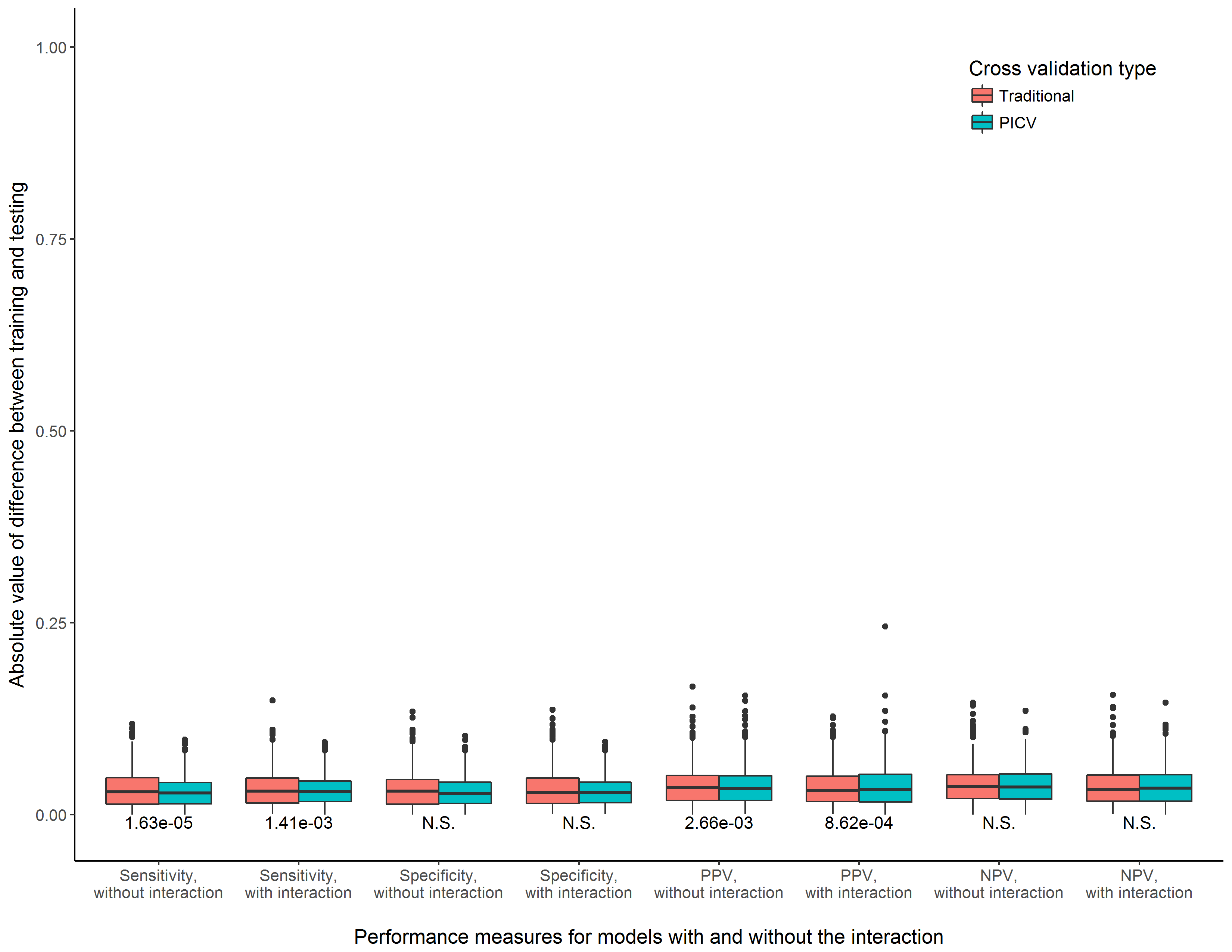


**Figure S10.** Consistency of training and testing performance measures for models with and without the interaction term, comparing a traditional cross validation procedure to PICV. Experimental scenario 10, prevalence = 0.5, n = 2000


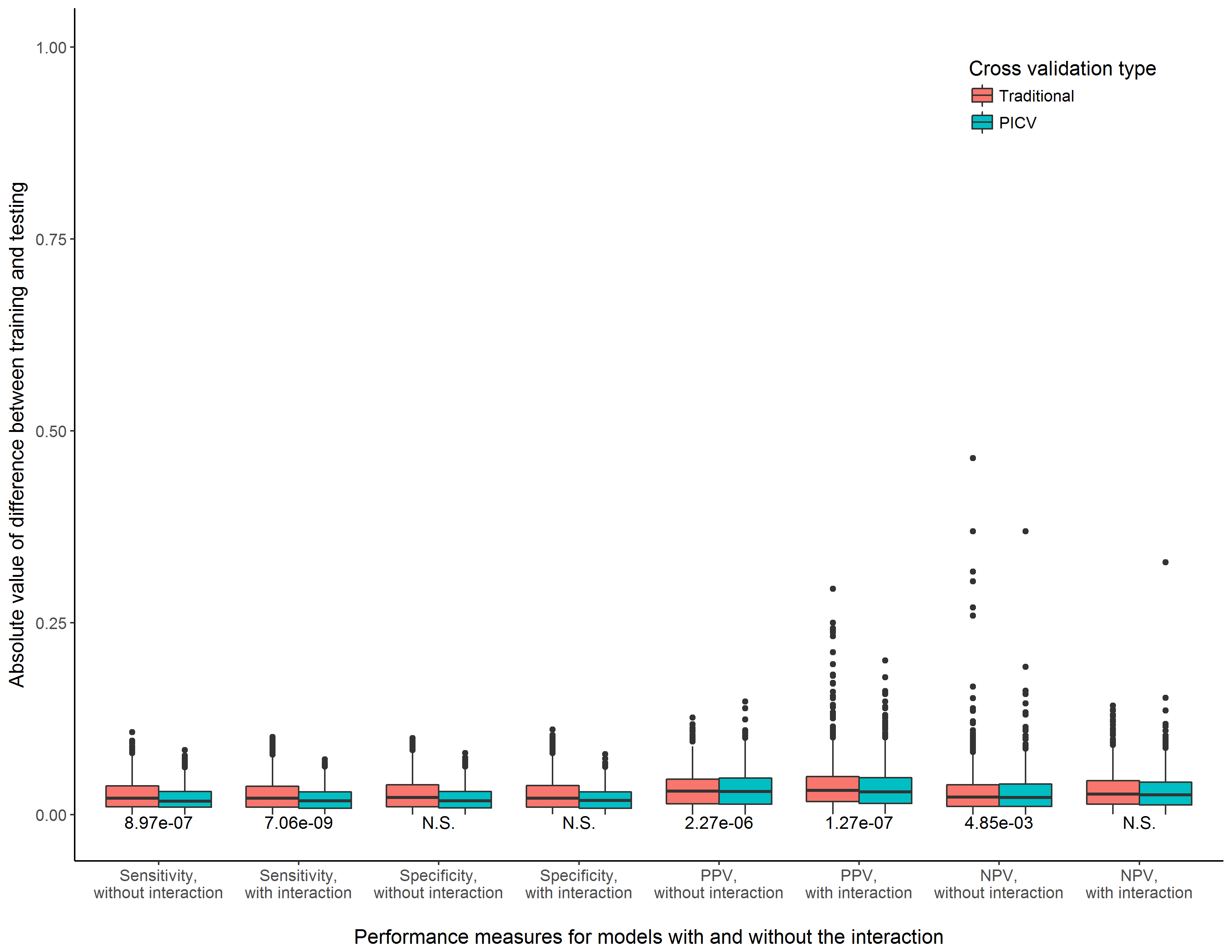


**Figure S11.** Consistency of training and testing performance measures for models with and without the interaction term, comparing a traditional cross validation procedure to PICV. Experimental scenario 11, prevalence = 0.5, n = 2000


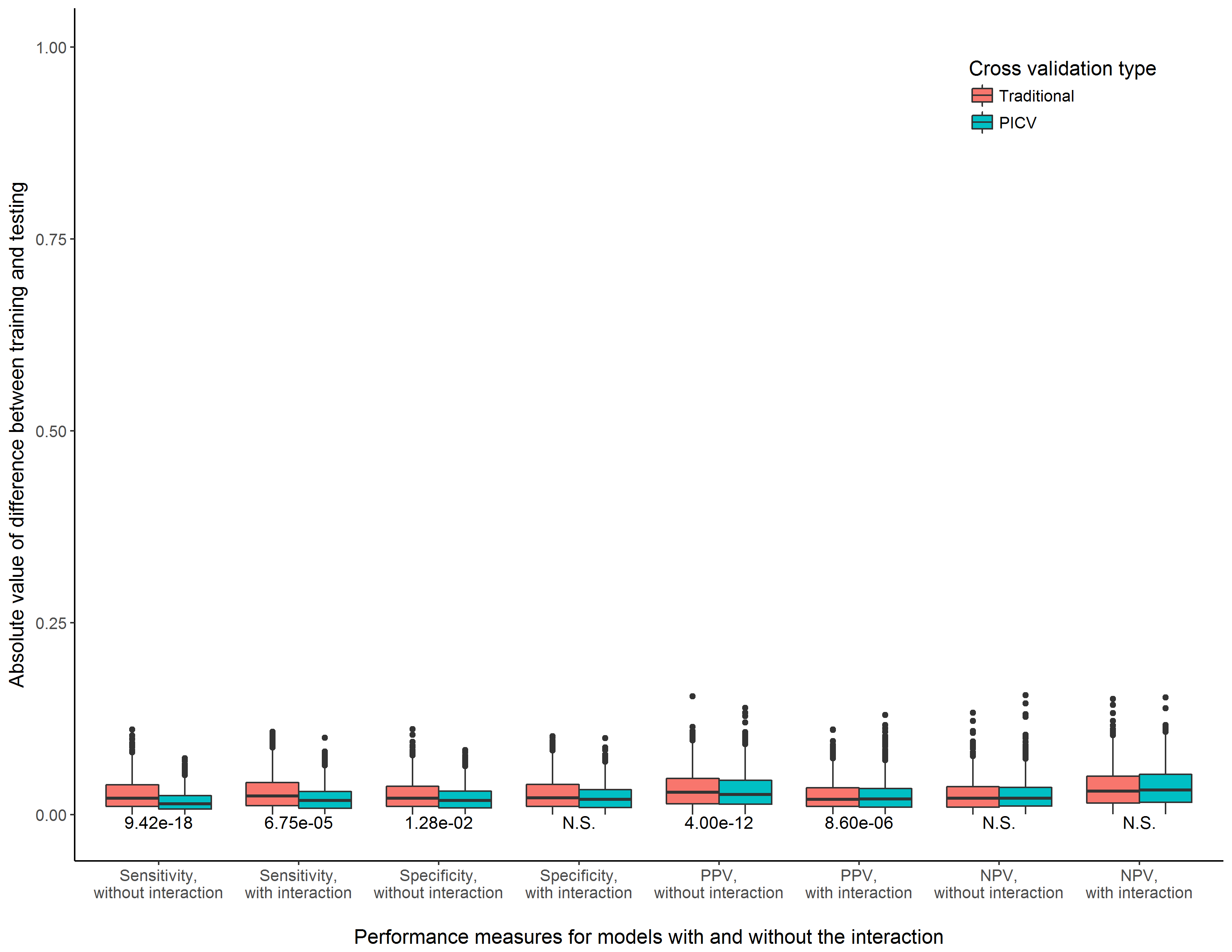


**Figure S12.** Consistency of training and testing performance measures for models with and without the interaction term, comparing a traditional cross validation procedure to PICV. Experimental scenario 12, prevalence = 0.5, n = 2000


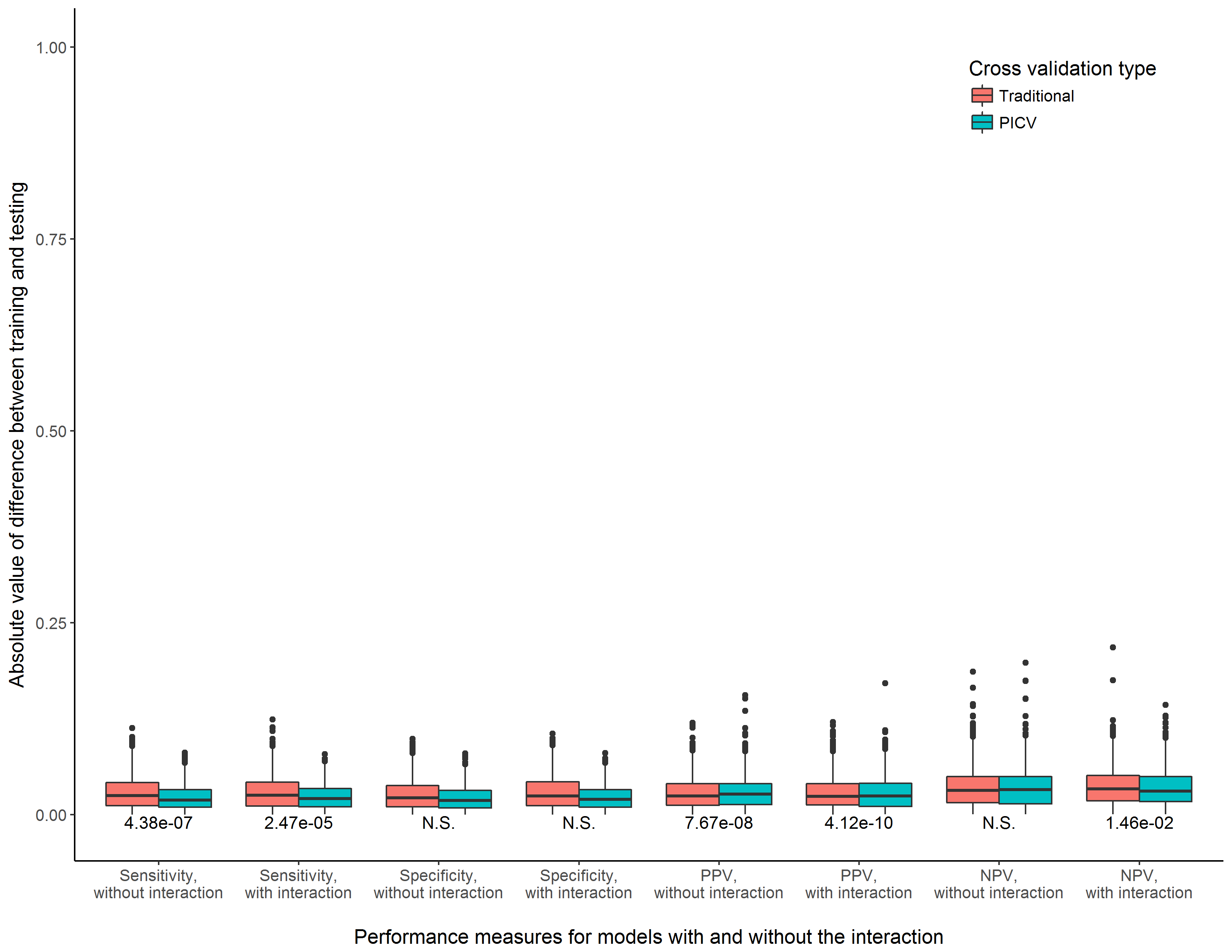


**Figure S13.** Consistency of training and testing performance measures for models with and without the interaction term, comparing a traditional cross validation procedure to PICV. Experimental scenario 13, prevalence = 0.5, n = 2000


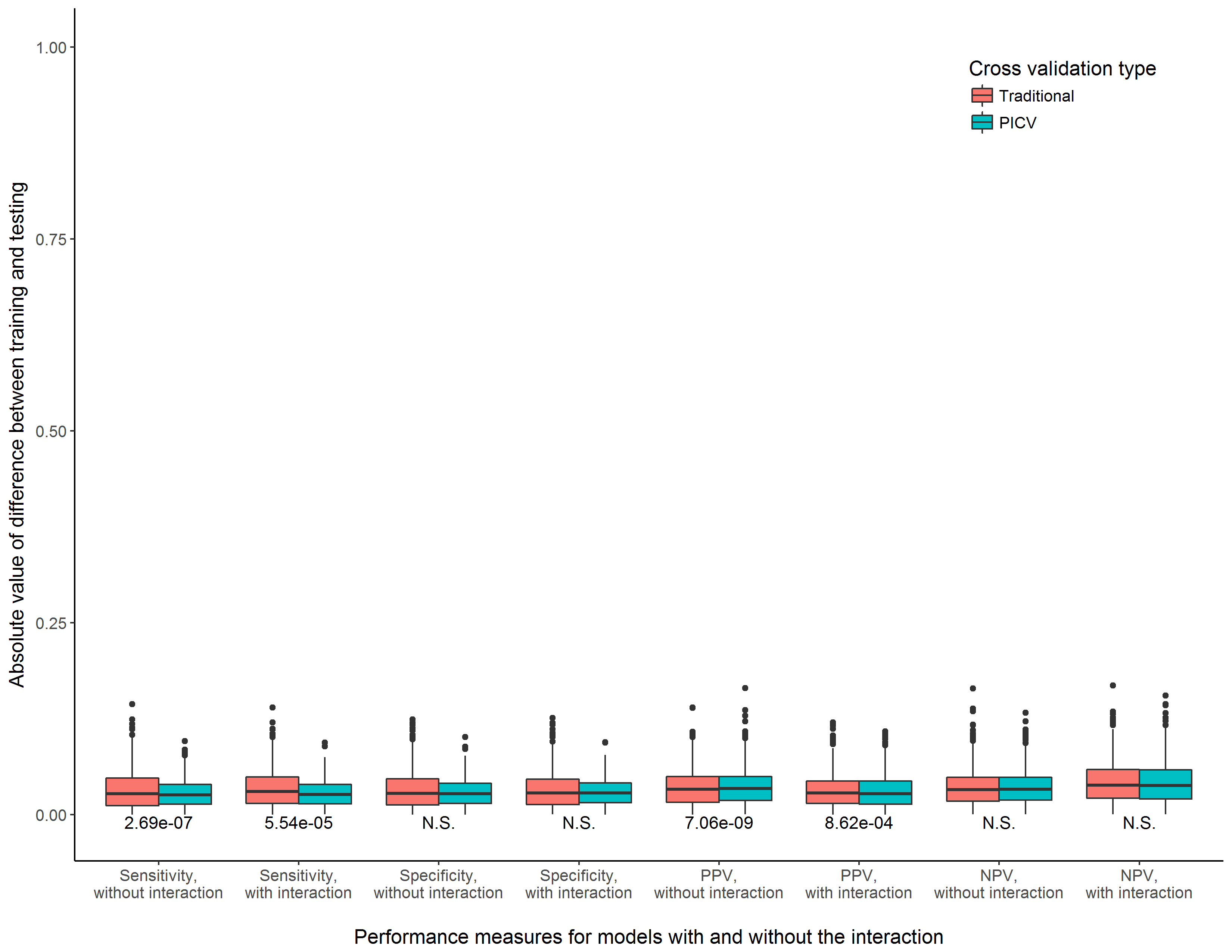


**Figure S14.** Consistency of training and testing performance measures for models with and without the interaction term, comparing a traditional cross validation procedure to PICV. Experimental scenario 14, prevalence = 0.5, n = 2000


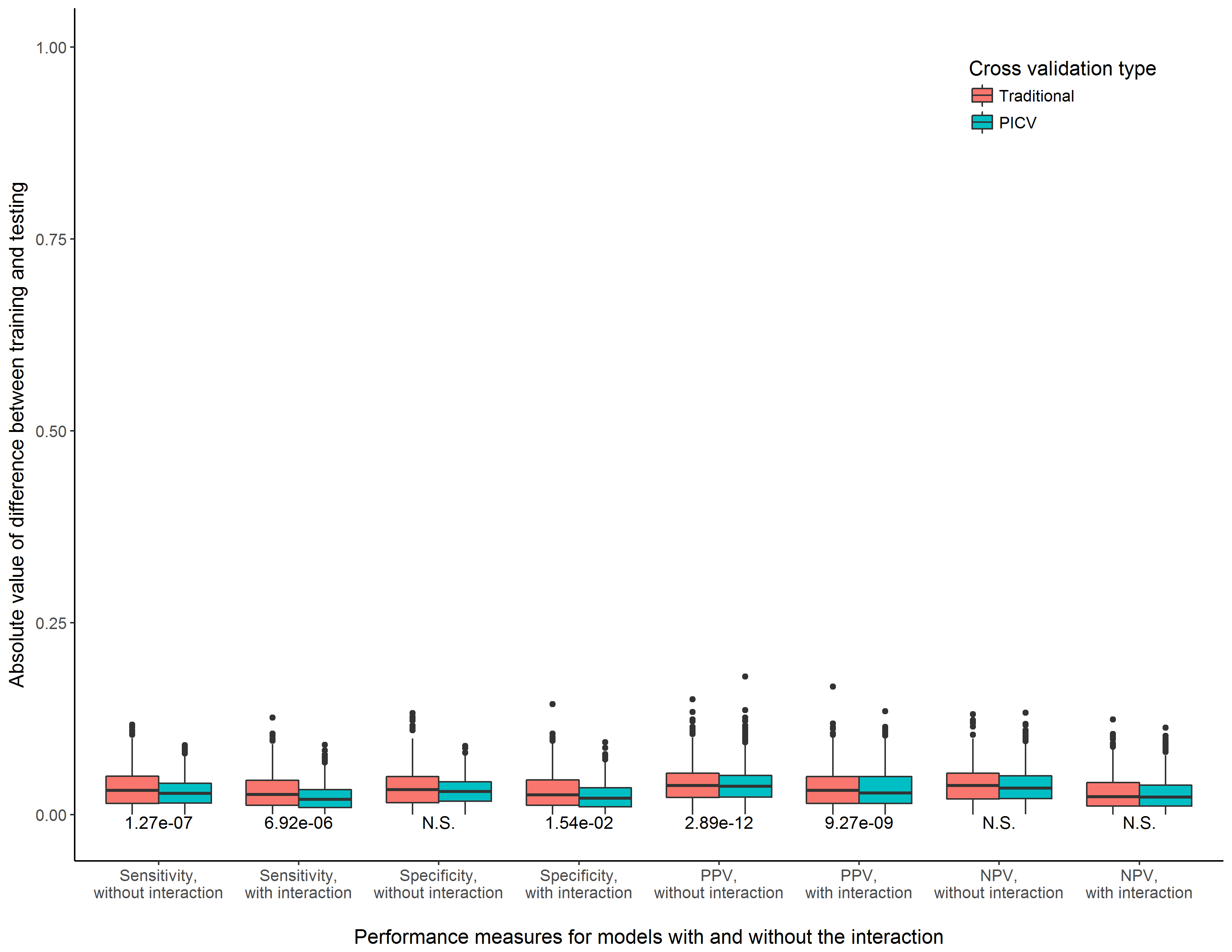


**Figure S15.** Consistency of training and testing performance measures for models with and without the interaction term, comparing a traditional cross validation procedure to PICV. Experimental scenario 15, prevalence = 0.5, n = 2000


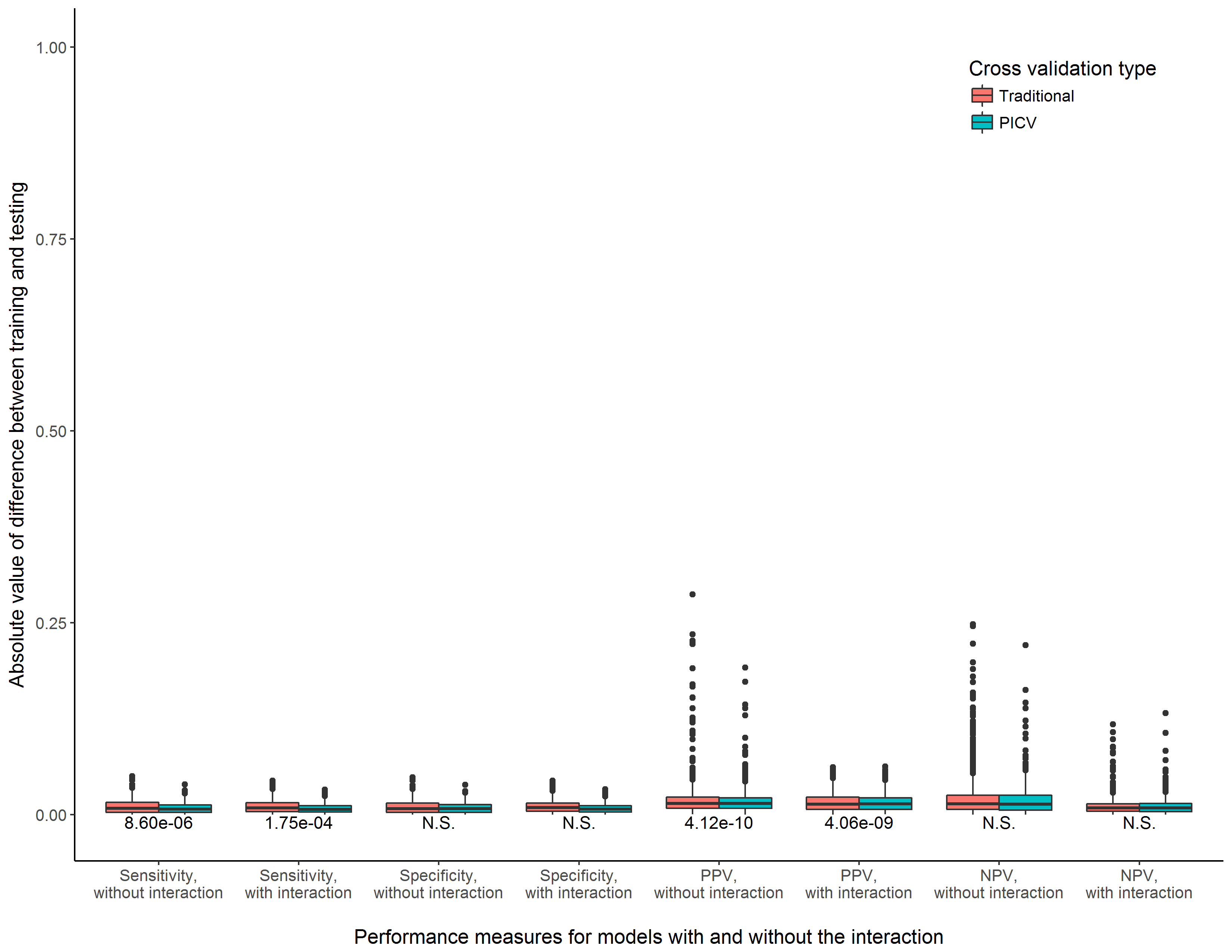


**Figure S16.** Consistency of training and testing performance measures for models with and without the interaction term, comparing a traditional cross validation procedure to PICV. Experimental scenario 1, prevalence = 0.5, n = 10000


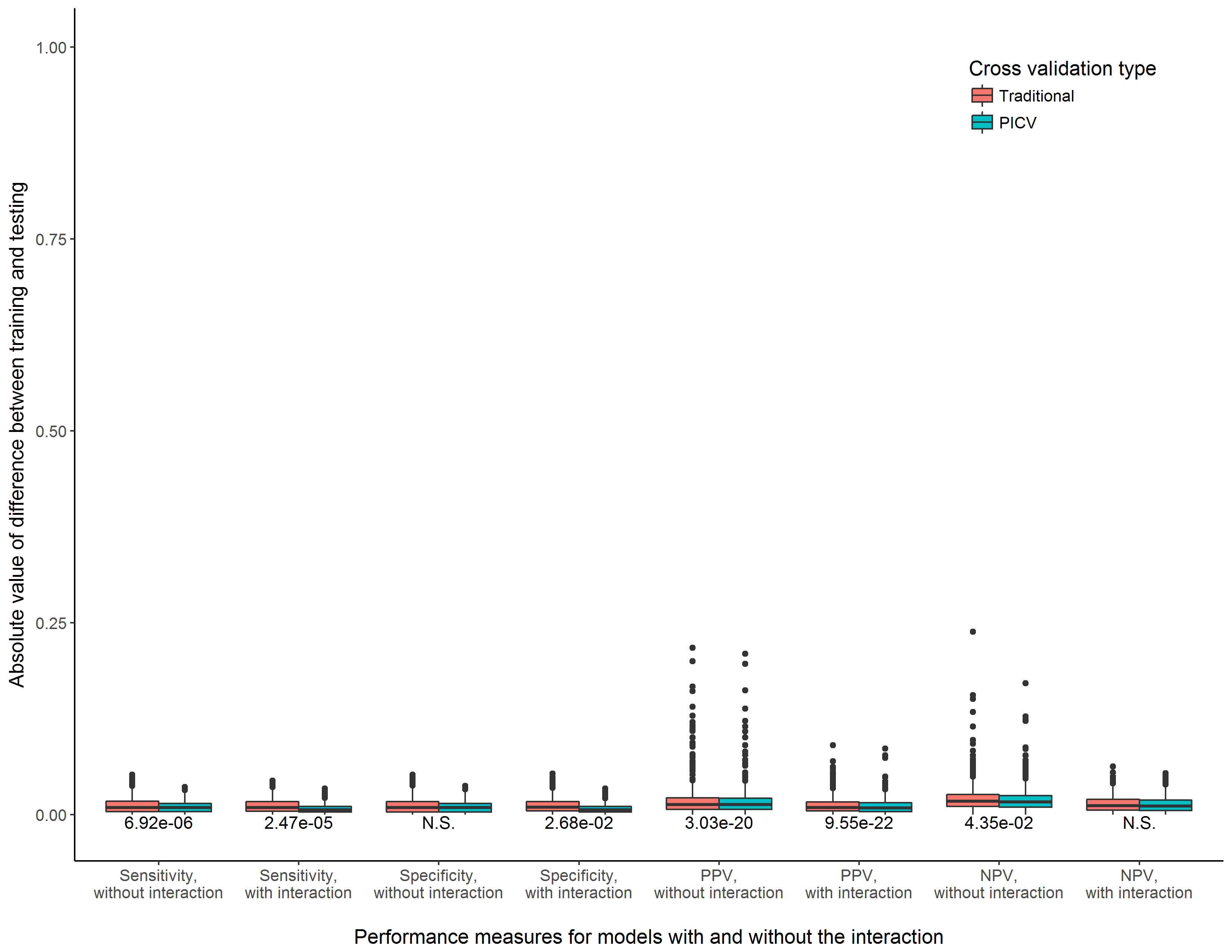


**Figure S17.** Consistency of training and testing performance measures for models with and without the interaction term, comparing a traditional cross validation procedure to PICV. Experimental scenario 2, prevalence = 0.5, n = 10000


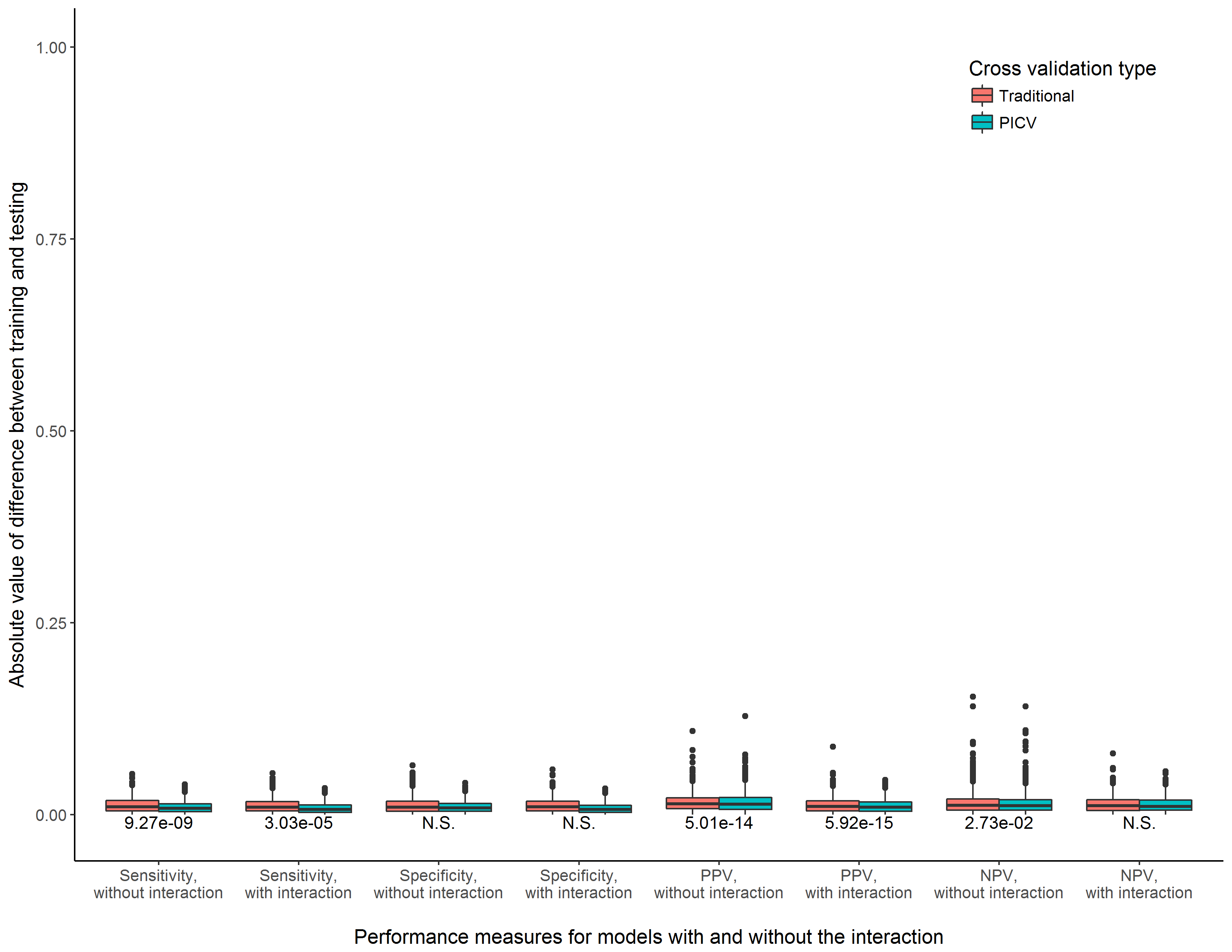


**Figure S18.** Consistency of training and testing performance measures for models with and without the interaction term, comparing a traditional cross validation procedure to PICV. Experimental scenario 3, prevalence = 0.5, n = 10000


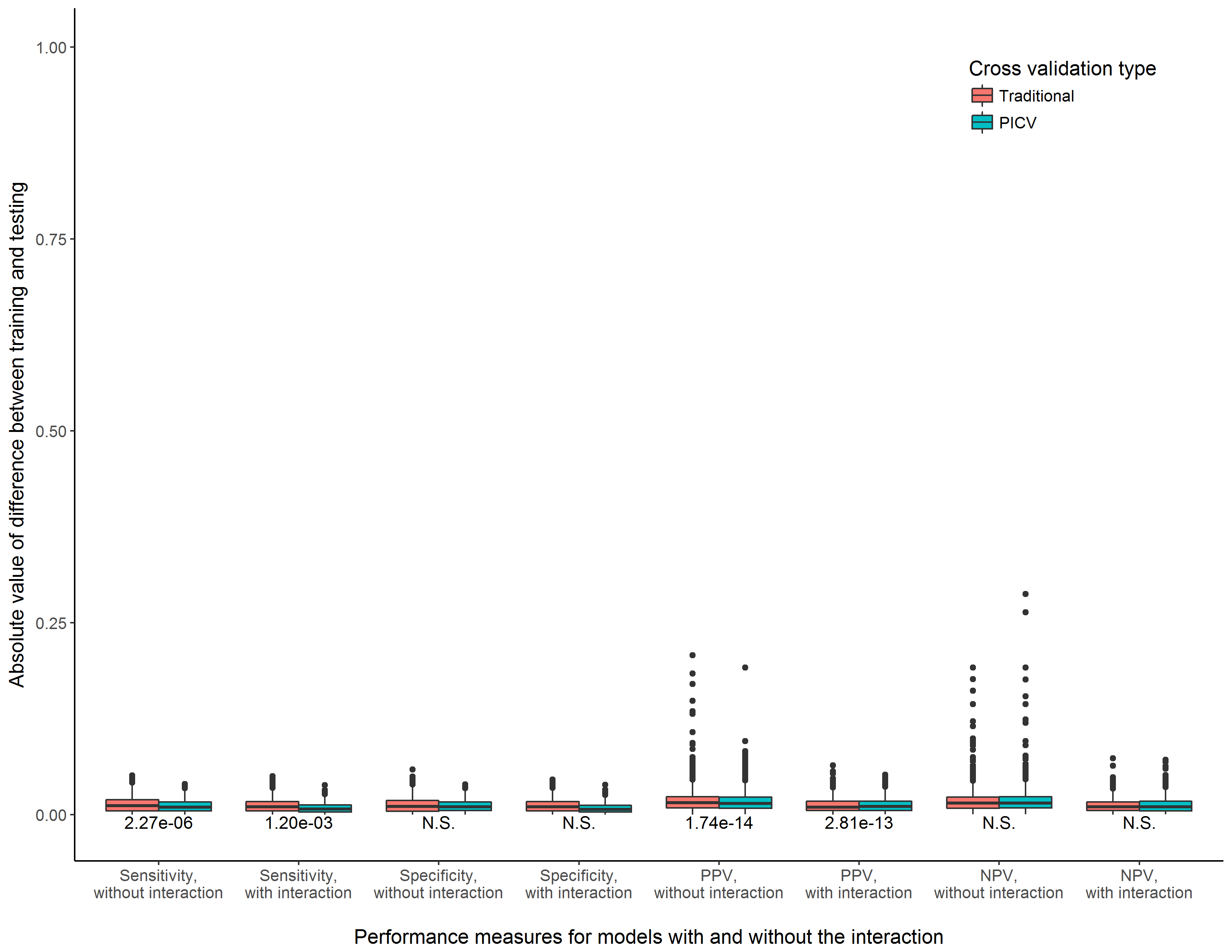


**Figure S19.** Consistency of training and testing performance measures for models with and without the interaction term, comparing a traditional cross validation procedure to PICV. Experimental scenario 4, prevalence = 0.5, n = 10000


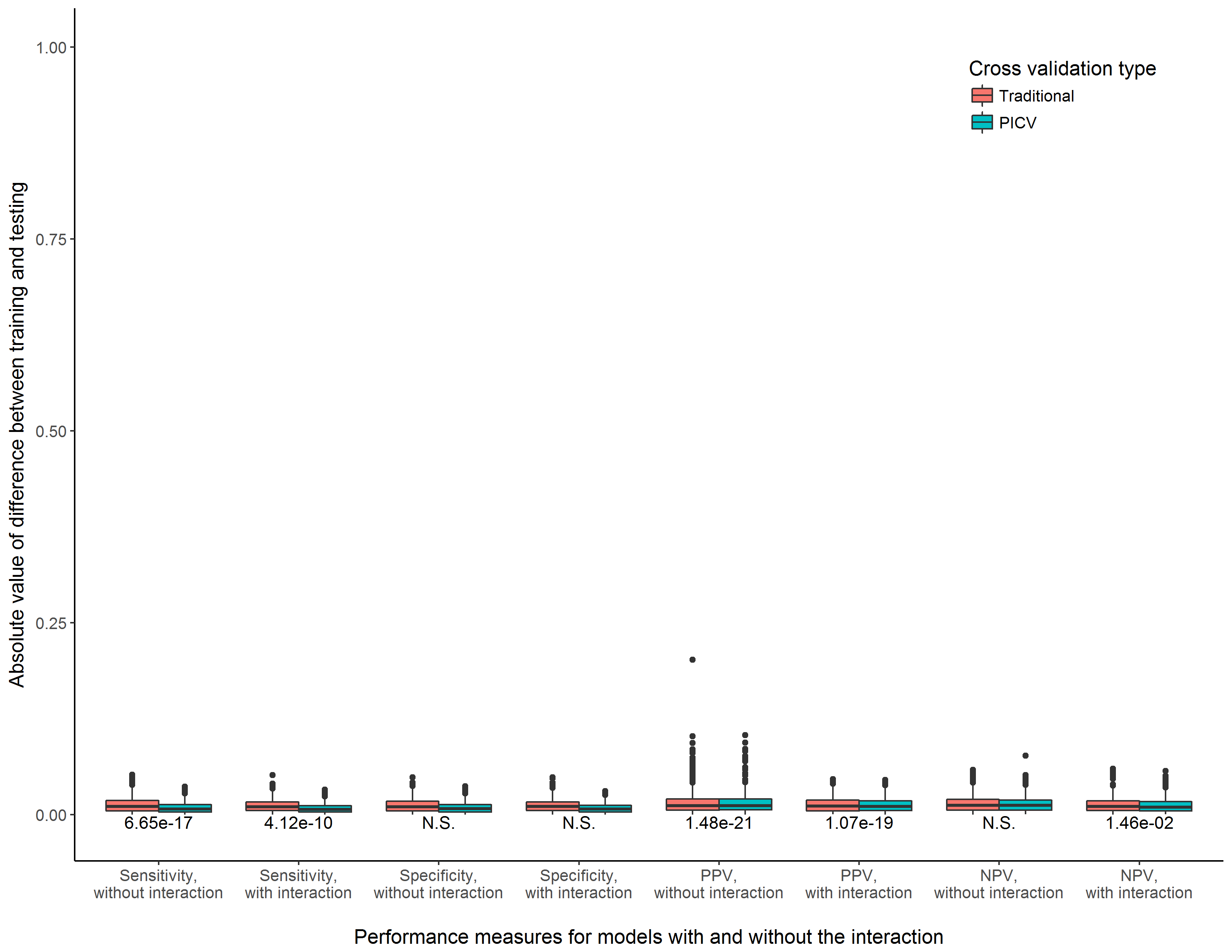


**Figure S20.** Consistency of training and testing performance measures for models with and without the interaction term, comparing a traditional cross validation procedure to PICV. Experimental scenario 5, prevalence = 0.5, n = 10000


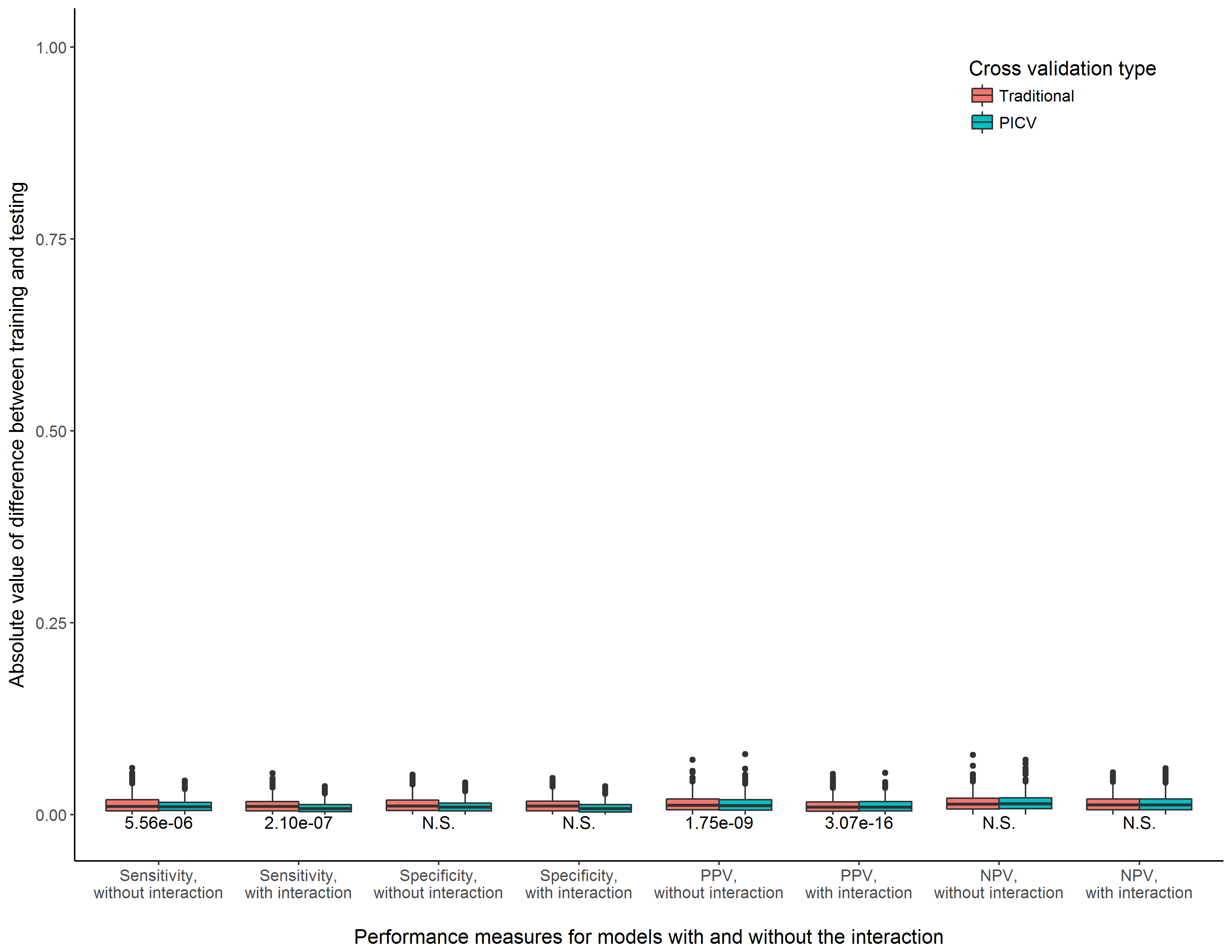


**Figure S21.** Consistency of training and testing performance measures for models with and without the interaction term, comparing a traditional cross validation procedure to PICV. Experimental scenario 6, prevalence = 0.5, n = 10000


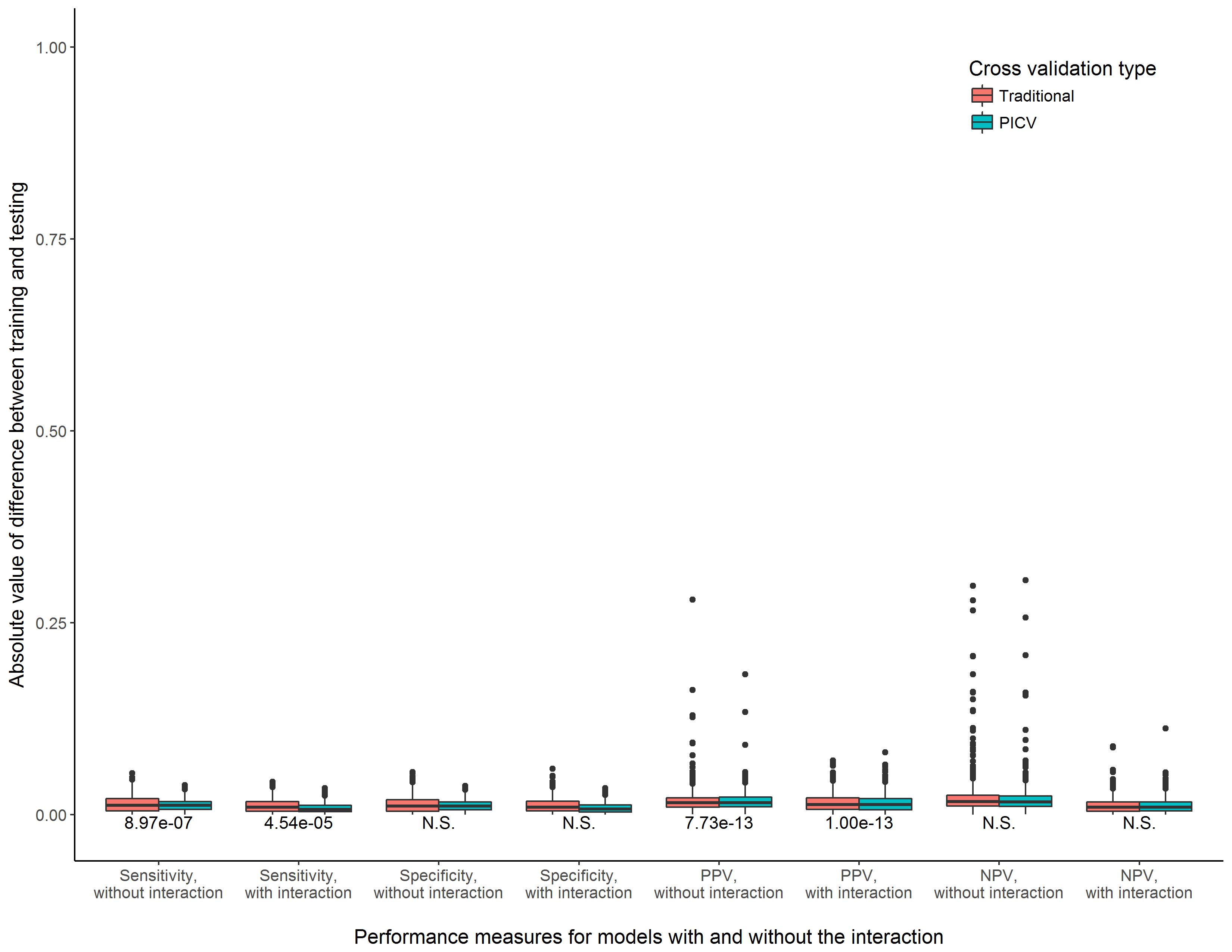


**Figure S22.** Consistency of training and testing performance measures for models with and without the interaction term, comparing a traditional cross validation procedure to PICV. Experimental scenario 7, prevalence = 0.5, n = 10000


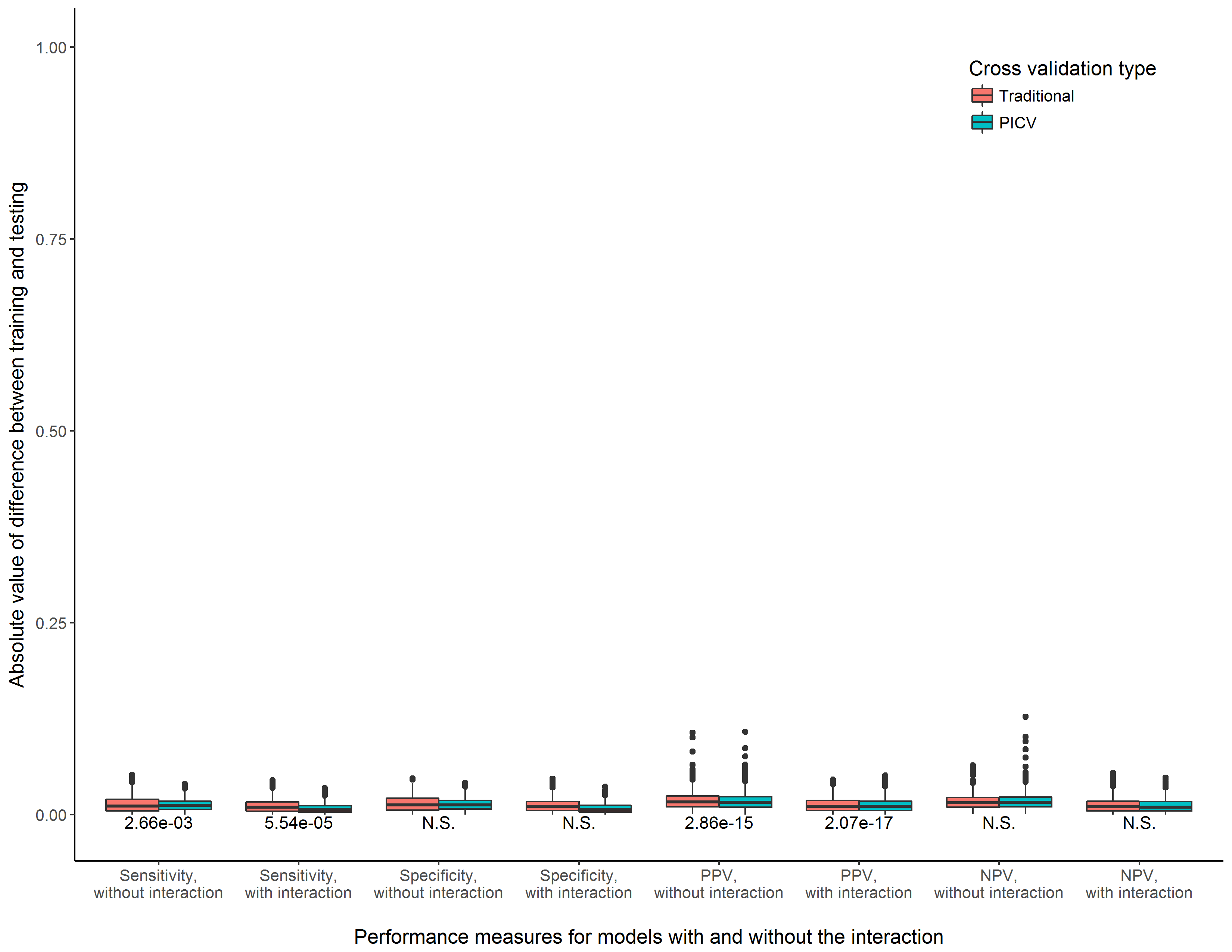


**Figure S23.** Consistency of training and testing performance measures for models with and without the interaction term, comparing a traditional cross validation procedure to PICV. Experimental scenario 8, prevalence = 0.5, n = 10000


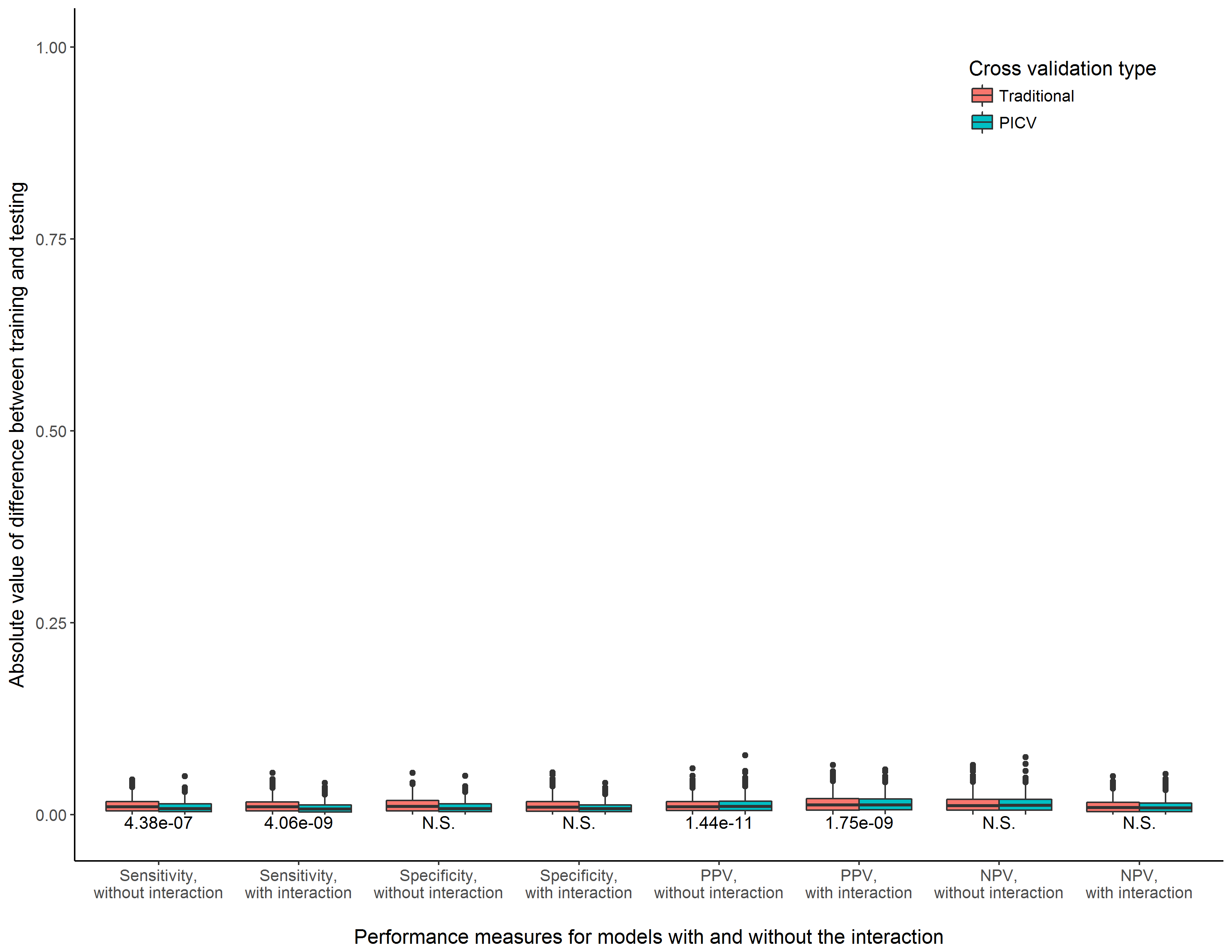


**Figure S24.** Consistency of training and testing performance measures for models with and without the interaction term, comparing a traditional cross validation procedure to PICV. Experimental scenario 9, prevalence = 0.5, n = 10000


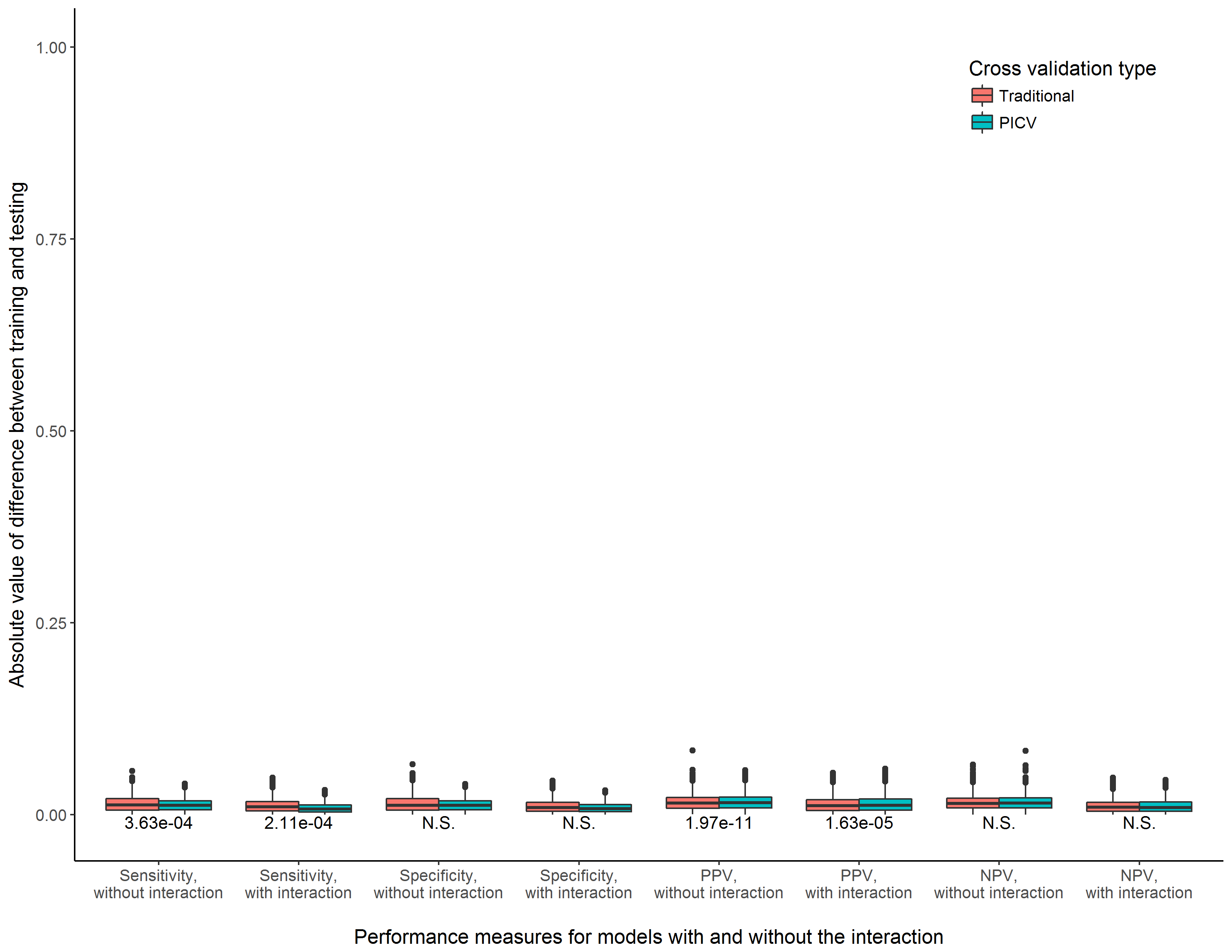


**Figure S25.** Consistency of training and testing performance measures for models with and without the interaction term, comparing a traditional cross validation procedure to PICV. Experimental scenario 10, prevalence = 0.5, n = 10000


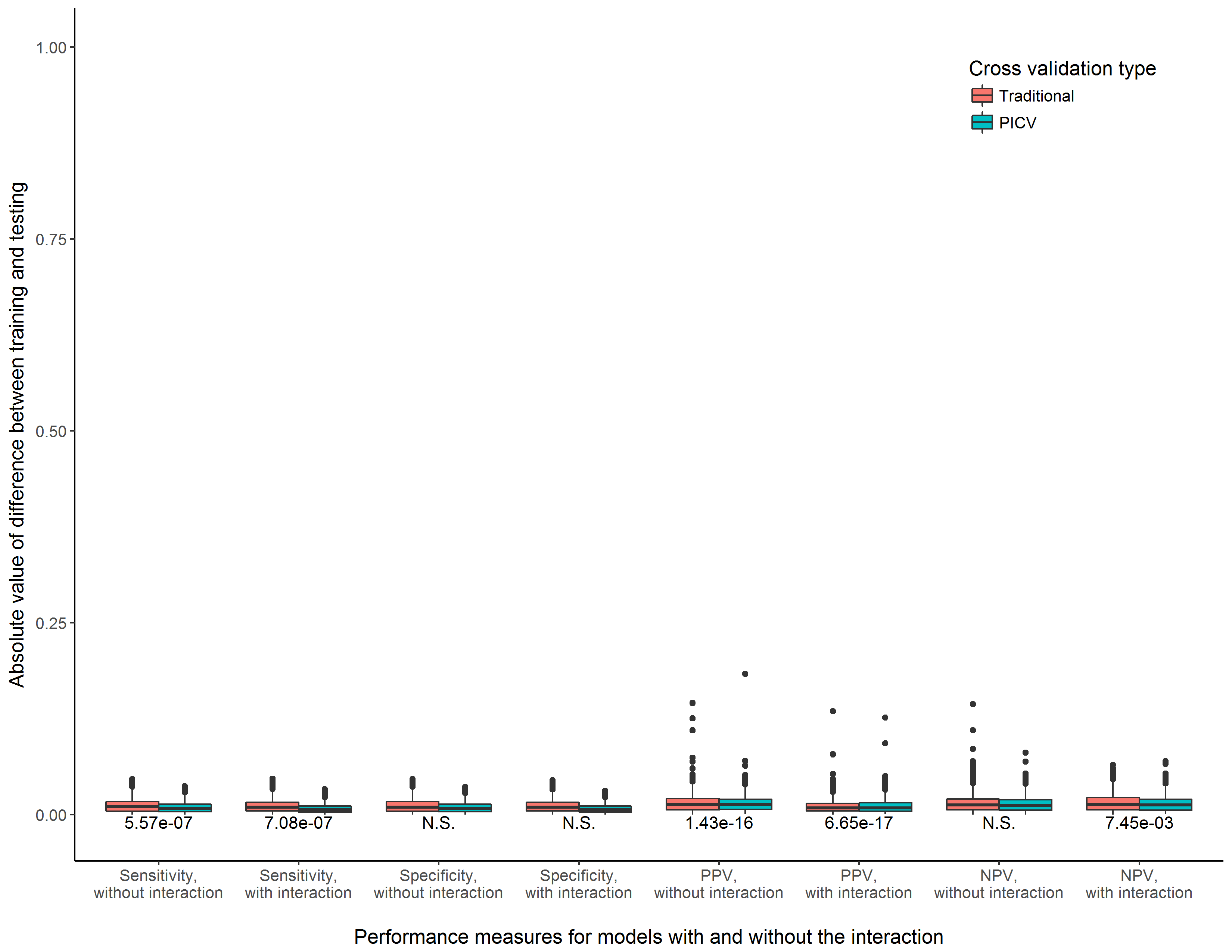


**Figure S26.** Consistency of training and testing performance measures for models with and without the interaction term, comparing a traditional cross validation procedure to PICV. Experimental scenario 11, prevalence = 0.5, n = 10000


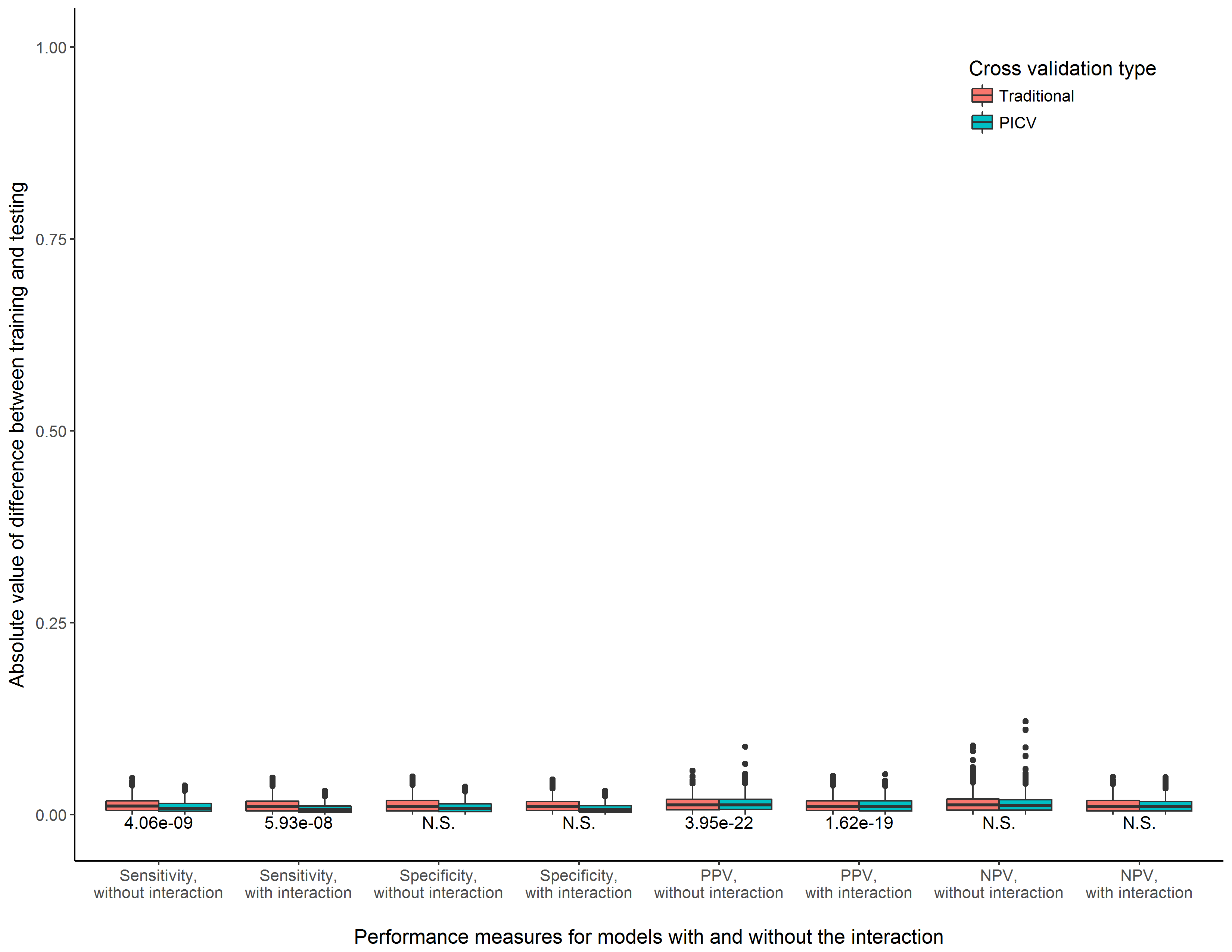


**Figure S27.** Consistency of training and testing performance measures for models with and without the interaction term, comparing a traditional cross validation procedure to PICV. Experimental scenario 12, prevalence = 0.5, n = 10000

**
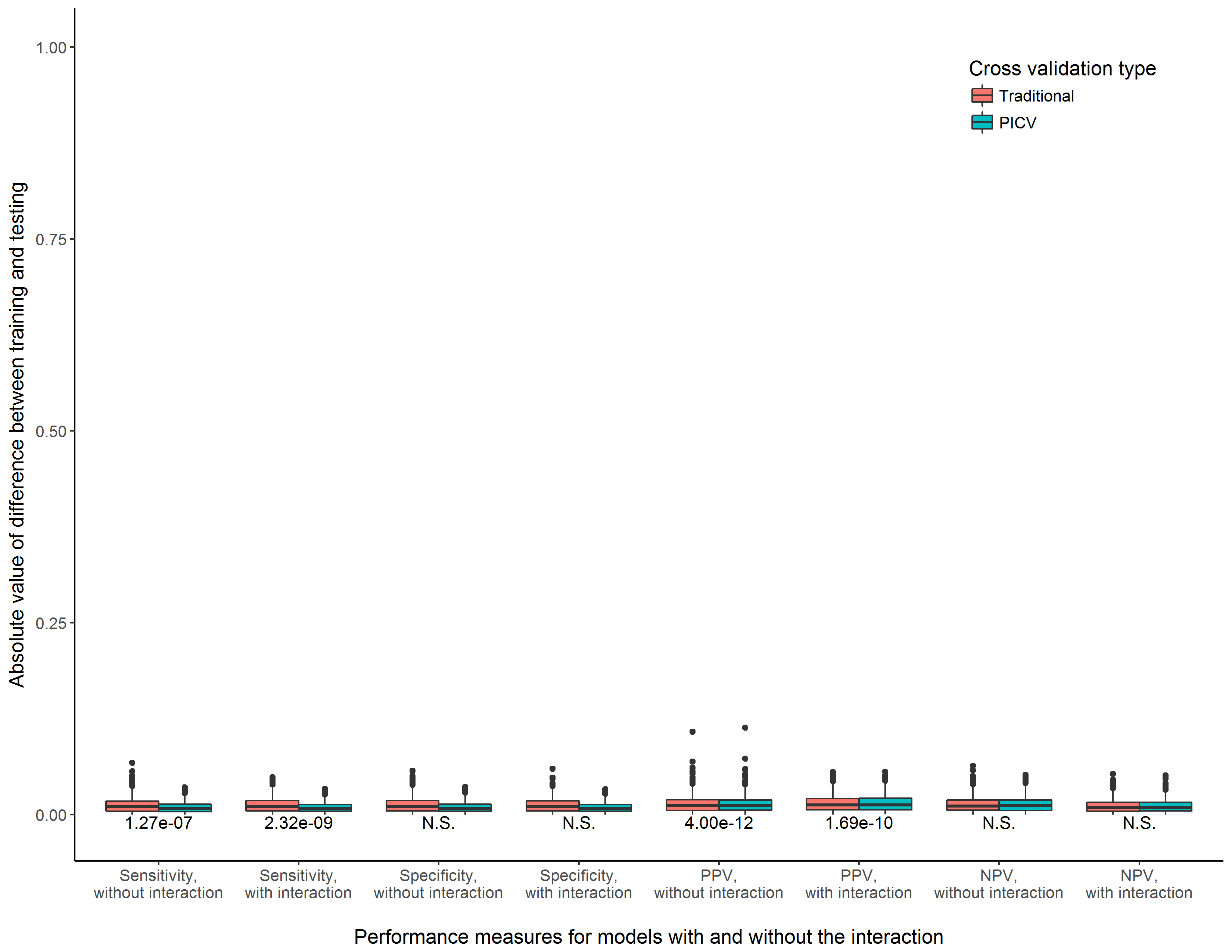
**

**Figure S28.** Consistency of training and testing performance measures for models with and without the interaction term, comparing a traditional cross validation procedure to PICV. Experimental scenario 13, prevalence = 0.5, n = 10000


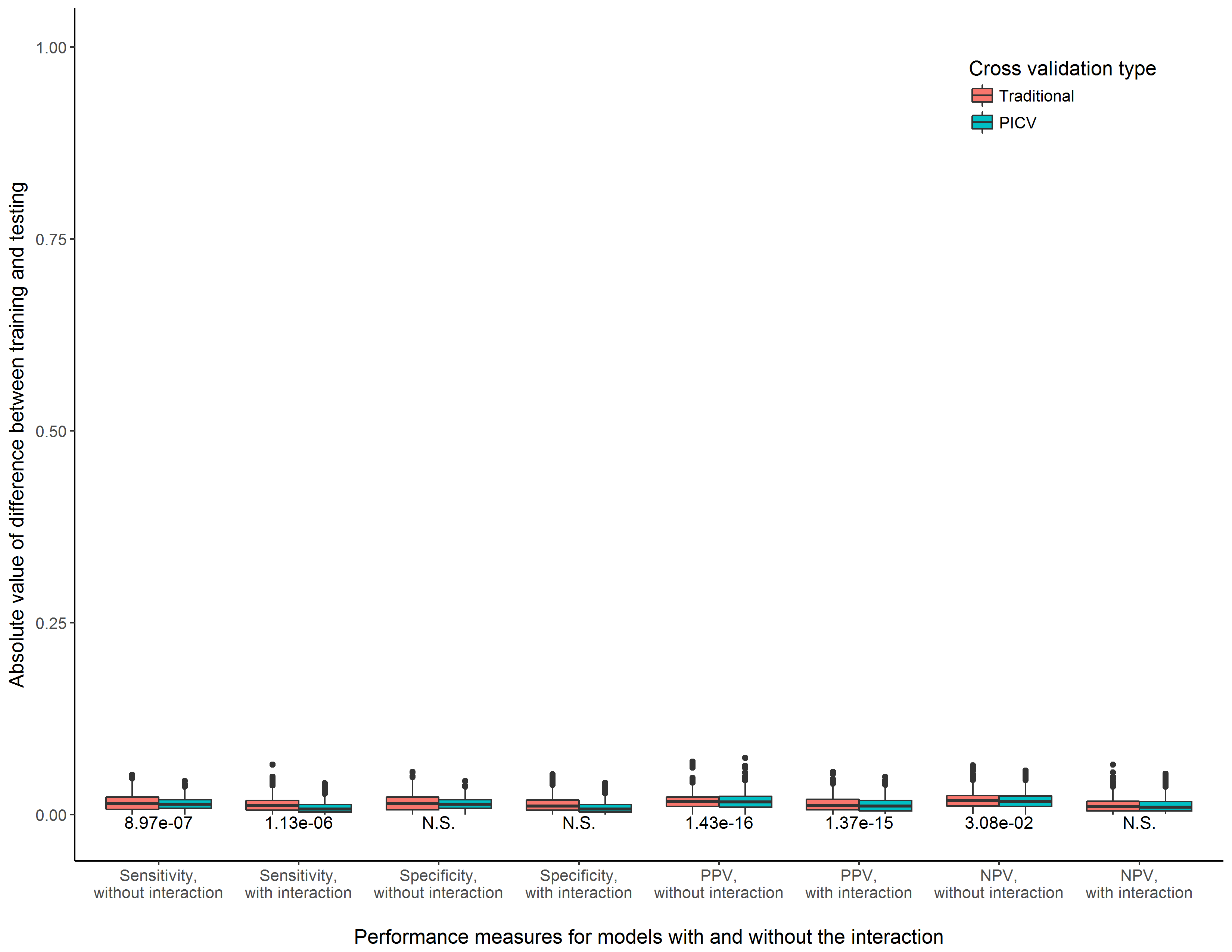


**Figure S29.** Consistency of training and testing performance measures for models with and without the interaction term, comparing a traditional cross validation procedure to PICV. Experimental scenario 14, prevalence = 0.5, n = 10000


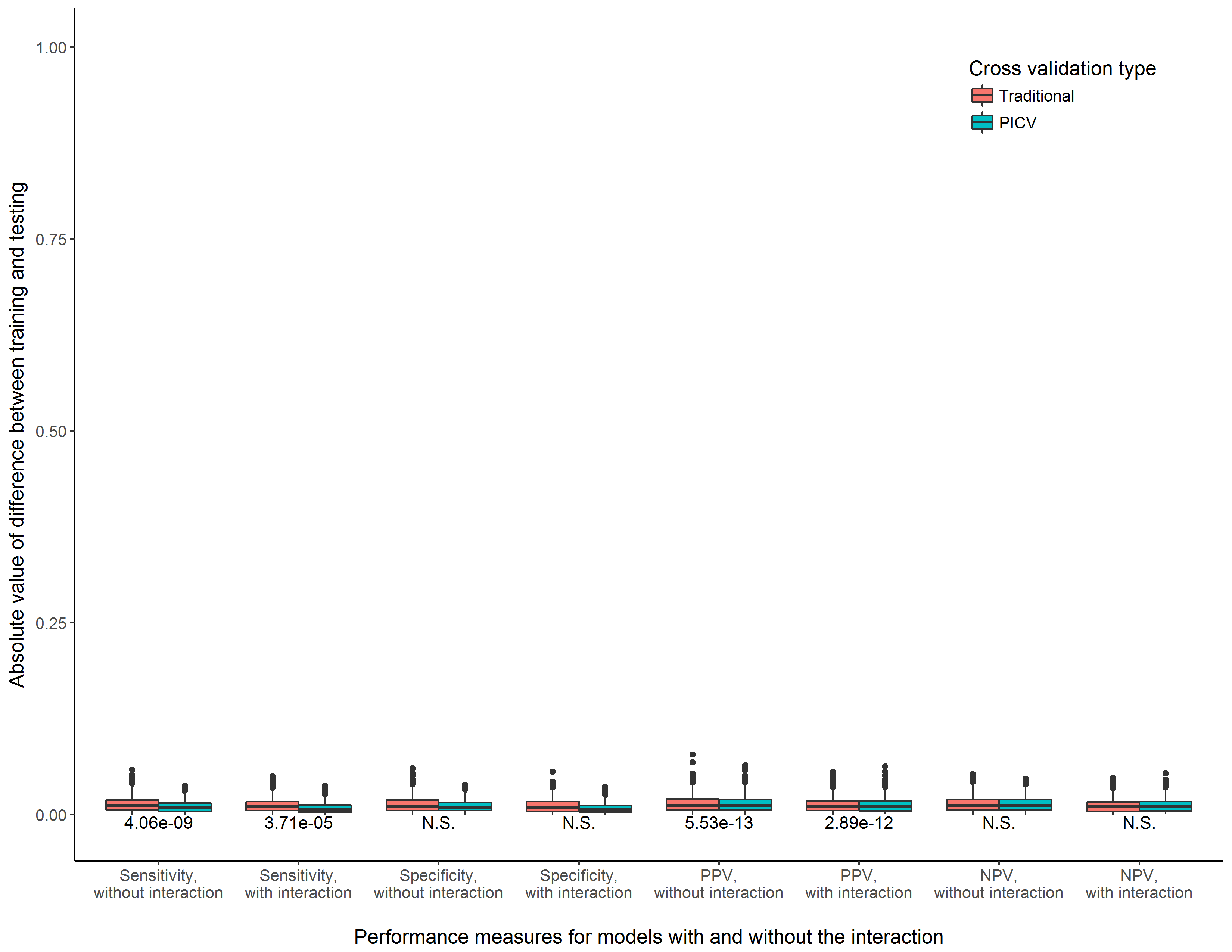


**Figure S30.** Consistency of training and testing performance measures for models with and without the interaction term, comparing a traditional cross validation procedure to PICV. Experimental scenario 15, prevalence = 0.5, n = 10000


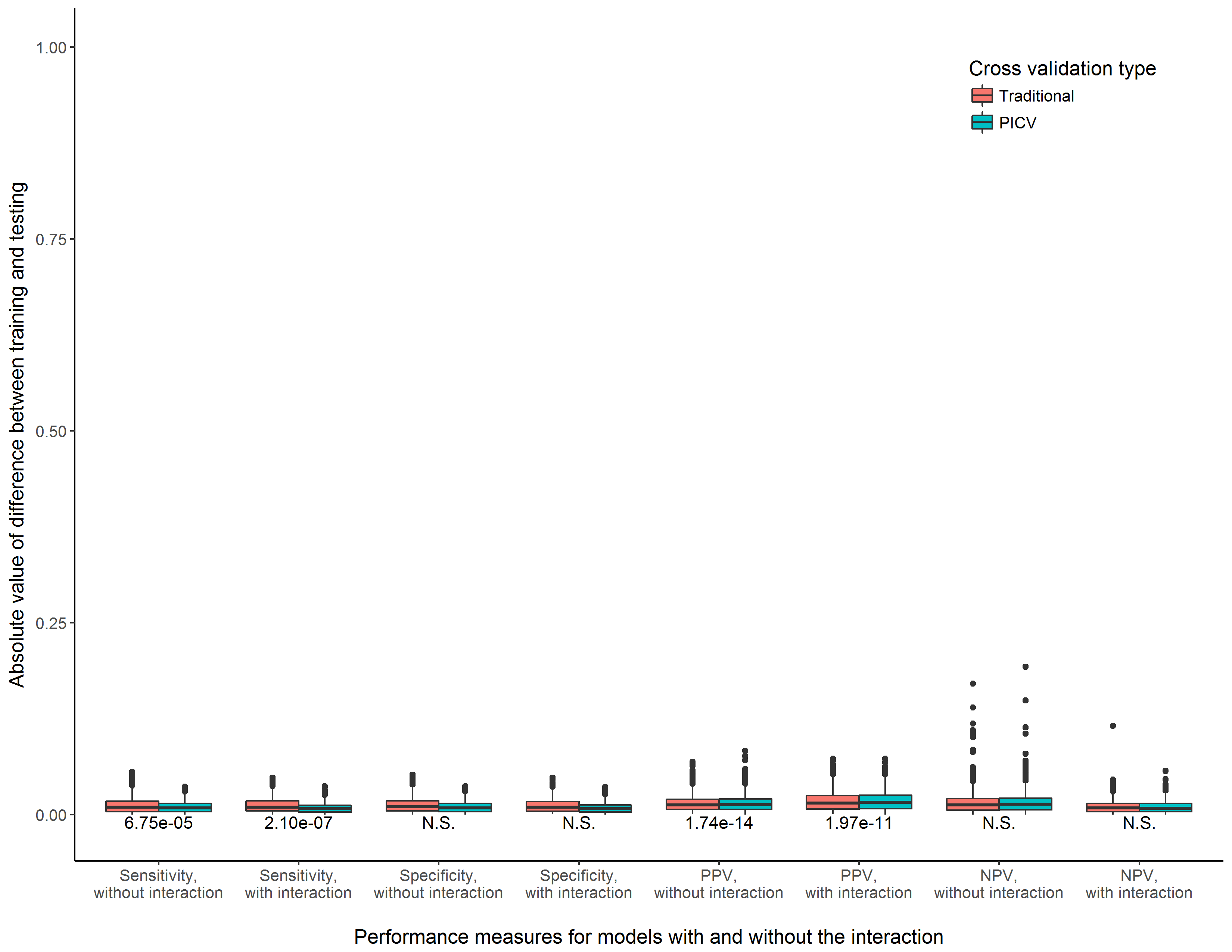


**Figure S31.** Consistency of training and testing performance measures for models with and without the interaction term, comparing a traditional cross validation procedure to PICV. Experimental scenario 1, prevalence = 0.1, n = 10000


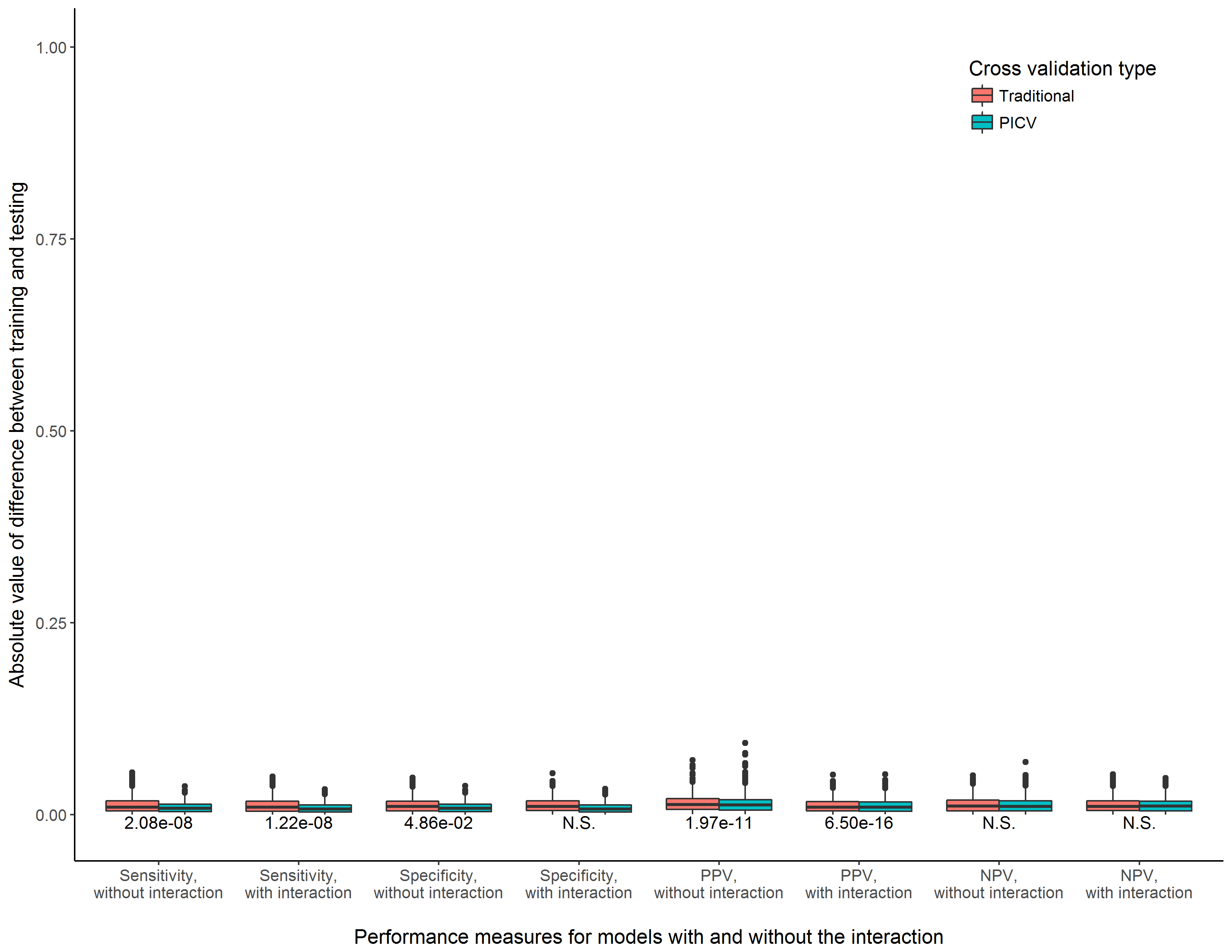


**Figure S32.** Consistency of training and testing performance measures for models with and without the interaction term, comparing a traditional cross validation procedure to PICV. Experimental scenario 2, prevalence = 0.1, n = 10000


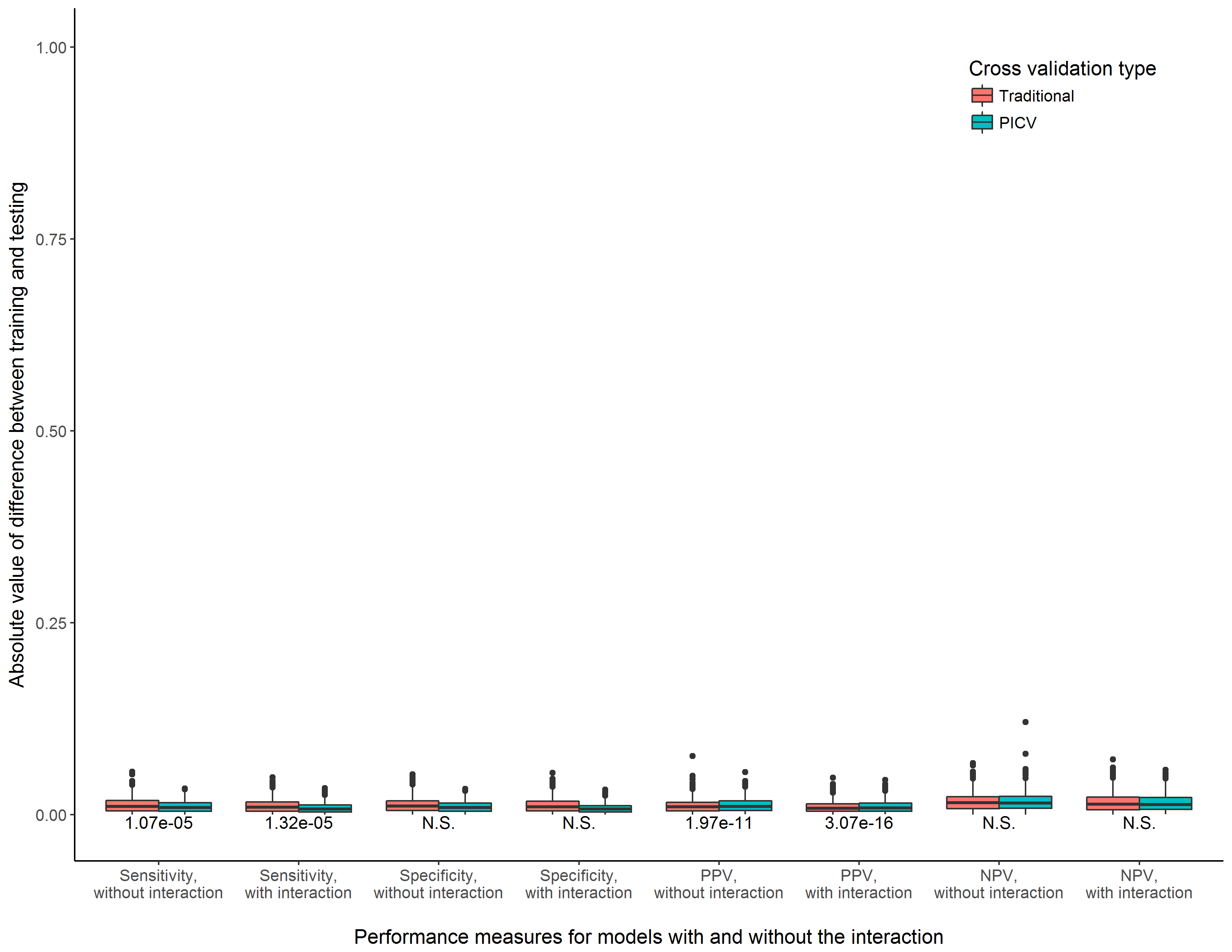


**Figure S33.** Consistency of training and testing performance measures for models with and without the interaction term, comparing a traditional cross validation procedure to PICV. Experimental scenario 3, prevalence = 0.1, n = 10000

**
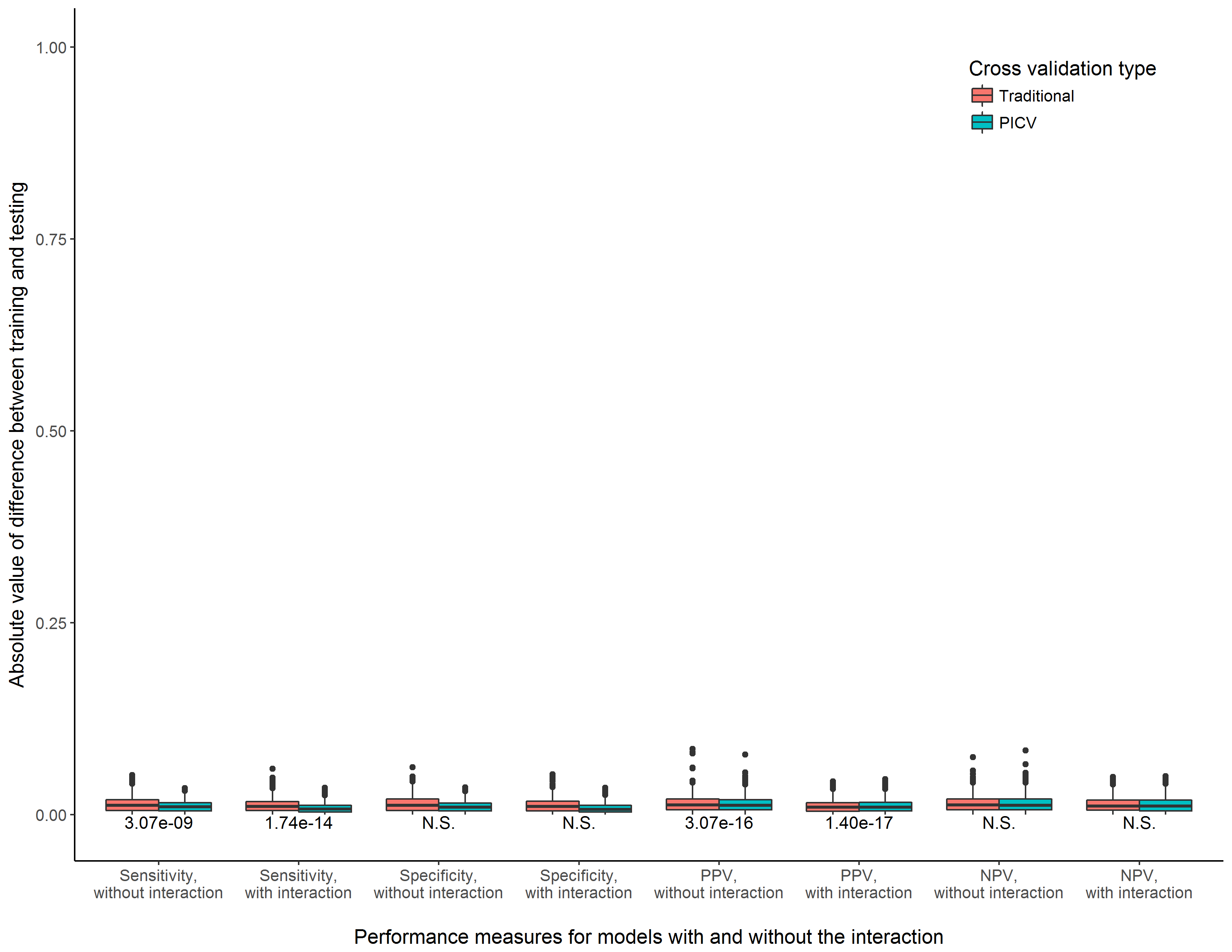
**

**Figure S34.** Consistency of training and testing performance measures for models with and without the interaction term, comparing a traditional cross validation procedure to PICV. Experimental scenario 4, prevalence = 0.1, n = 10000


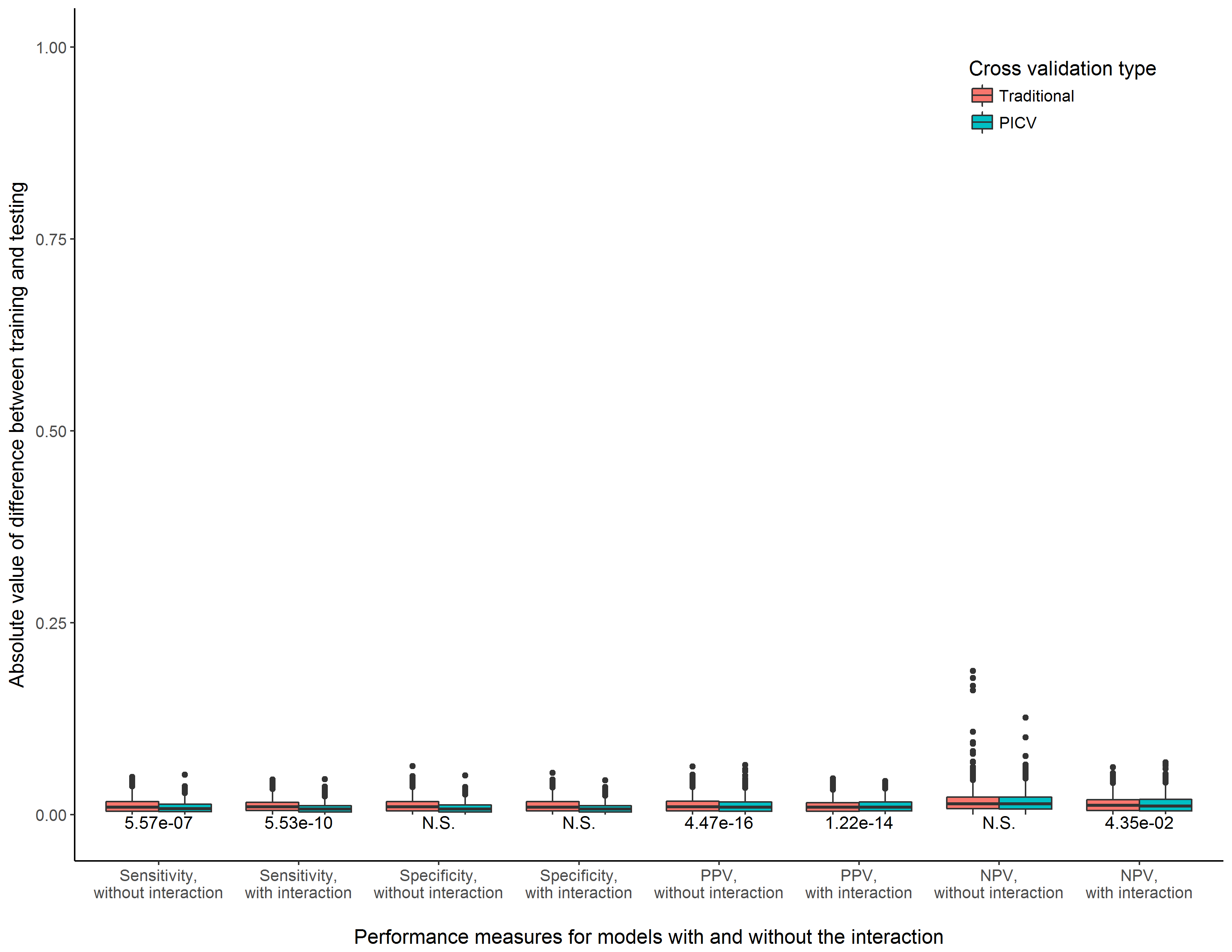


**Figure S35.** Consistency of training and testing performance measures for models with and without the interaction term, comparing a traditional cross validation procedure to PICV. Experimental scenario 5, prevalence = 0.1, n = 10000


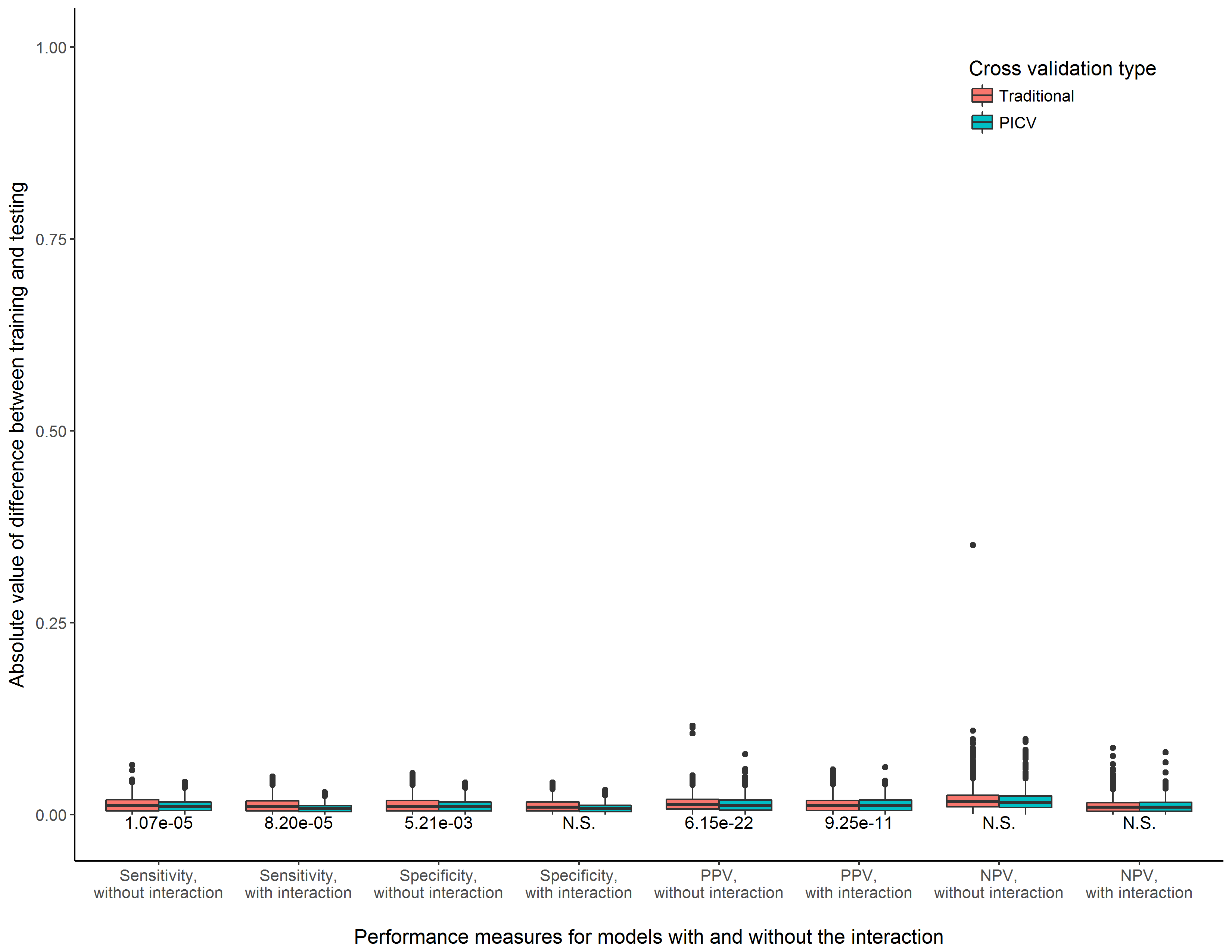


**Figure S36.** Consistency of training and testing performance measures for models with and without the interaction term, comparing a traditional cross validation procedure to PICV. Experimental scenario 6, prevalence = 0.1, n = 10000


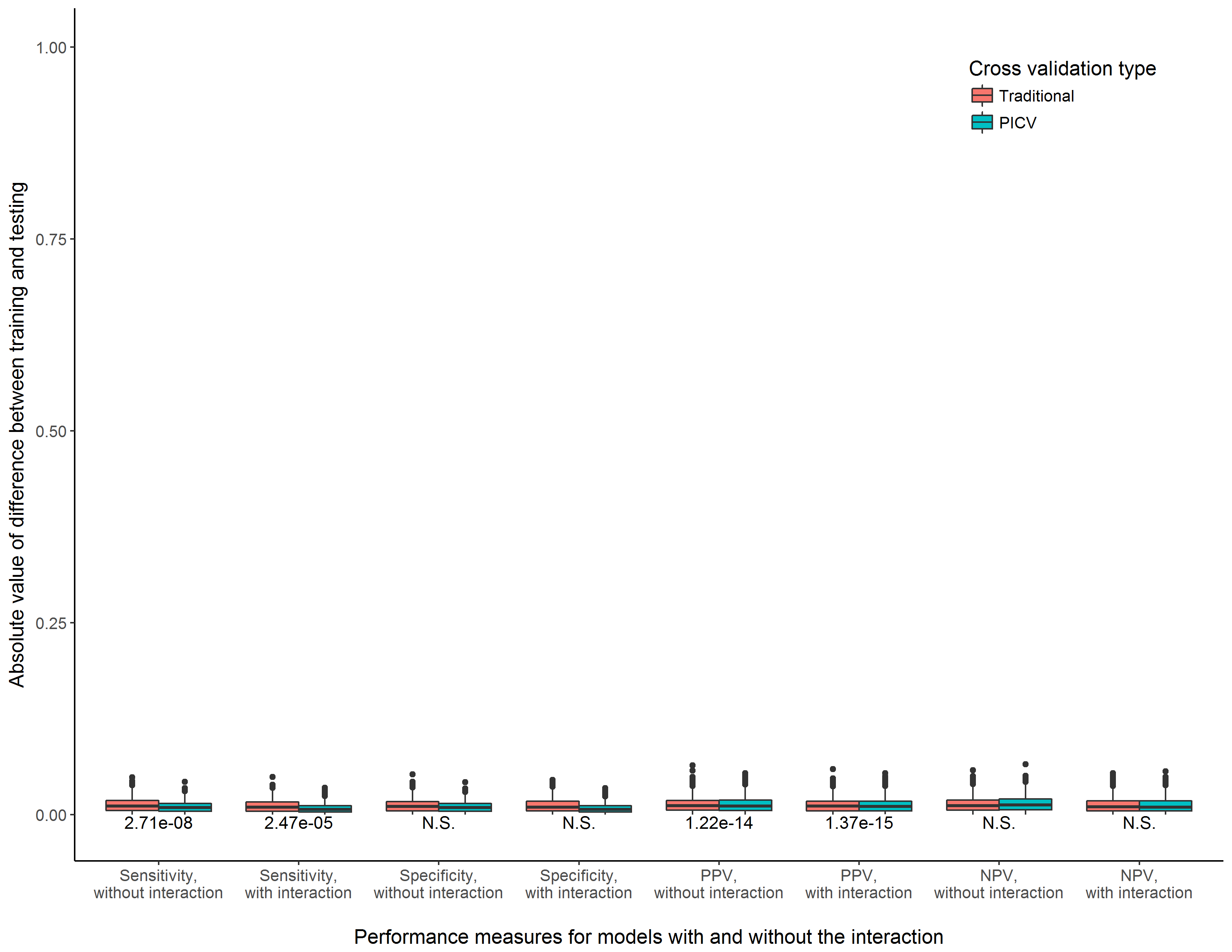


**Figure S37.** Consistency of training and testing performance measures for models with and without the interaction term, comparing a traditional cross validation procedure to PICV. Experimental scenario 7, prevalence = 0.1, n = 10000

**
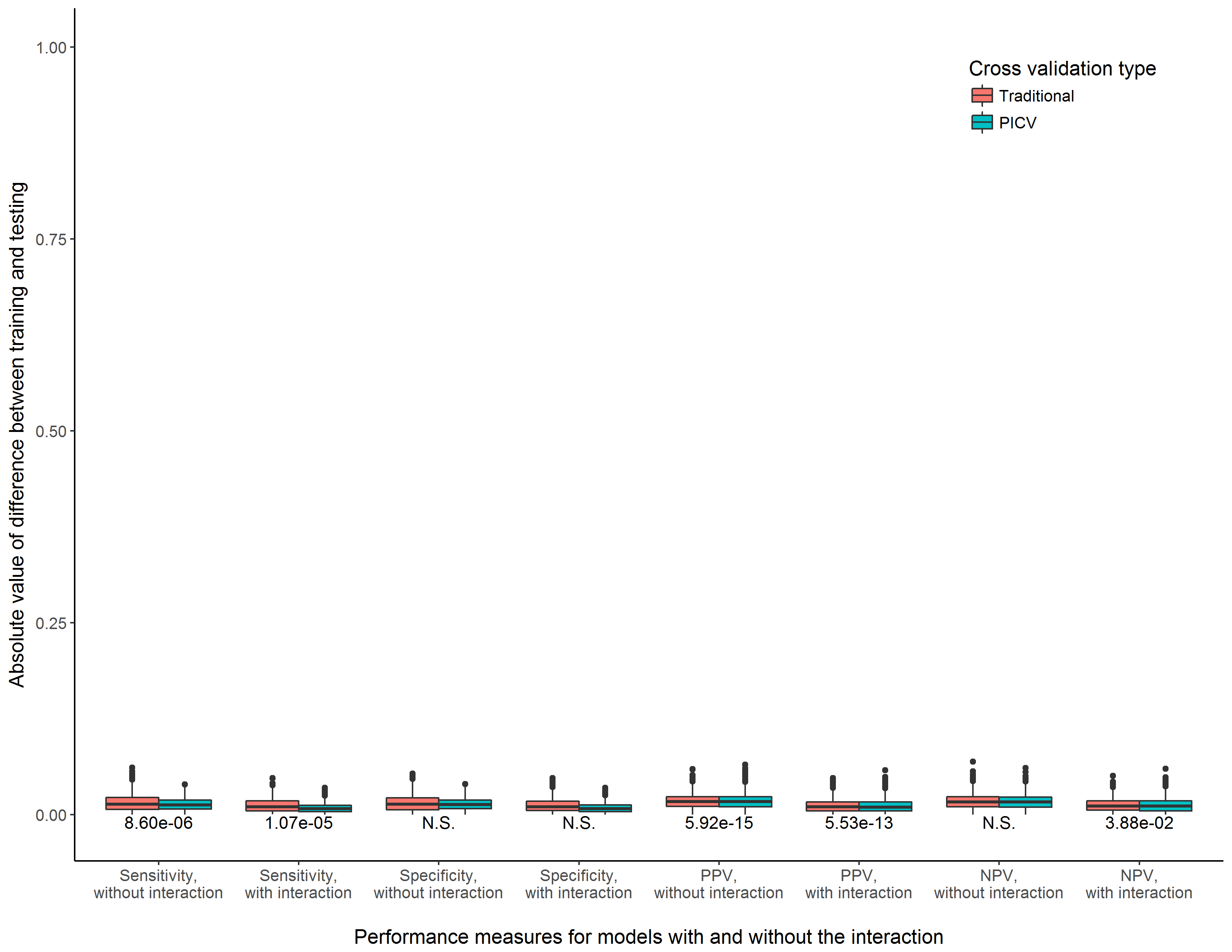
**

**Figure S38.** Consistency of training and testing performance measures for models with and without the interaction term, comparing a traditional cross validation procedure to PICV. Experimental scenario 8, prevalence = 0.1, n = 10000


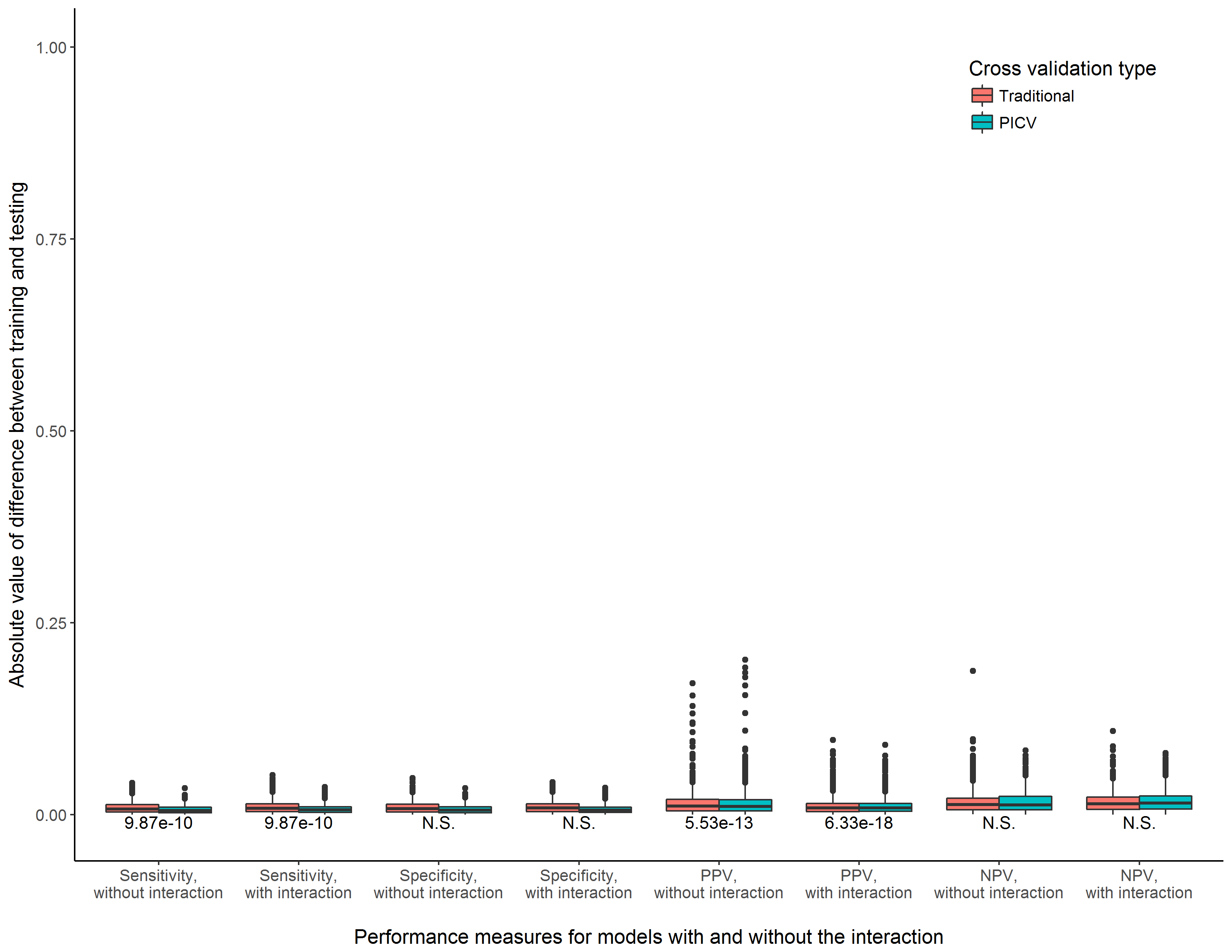


**Figure S39.** Consistency of training and testing performance measures for models with and without the interaction term, comparing a traditional cross validation procedure to PICV. Experimental scenario 9, prevalence = 0.1, n = 10000


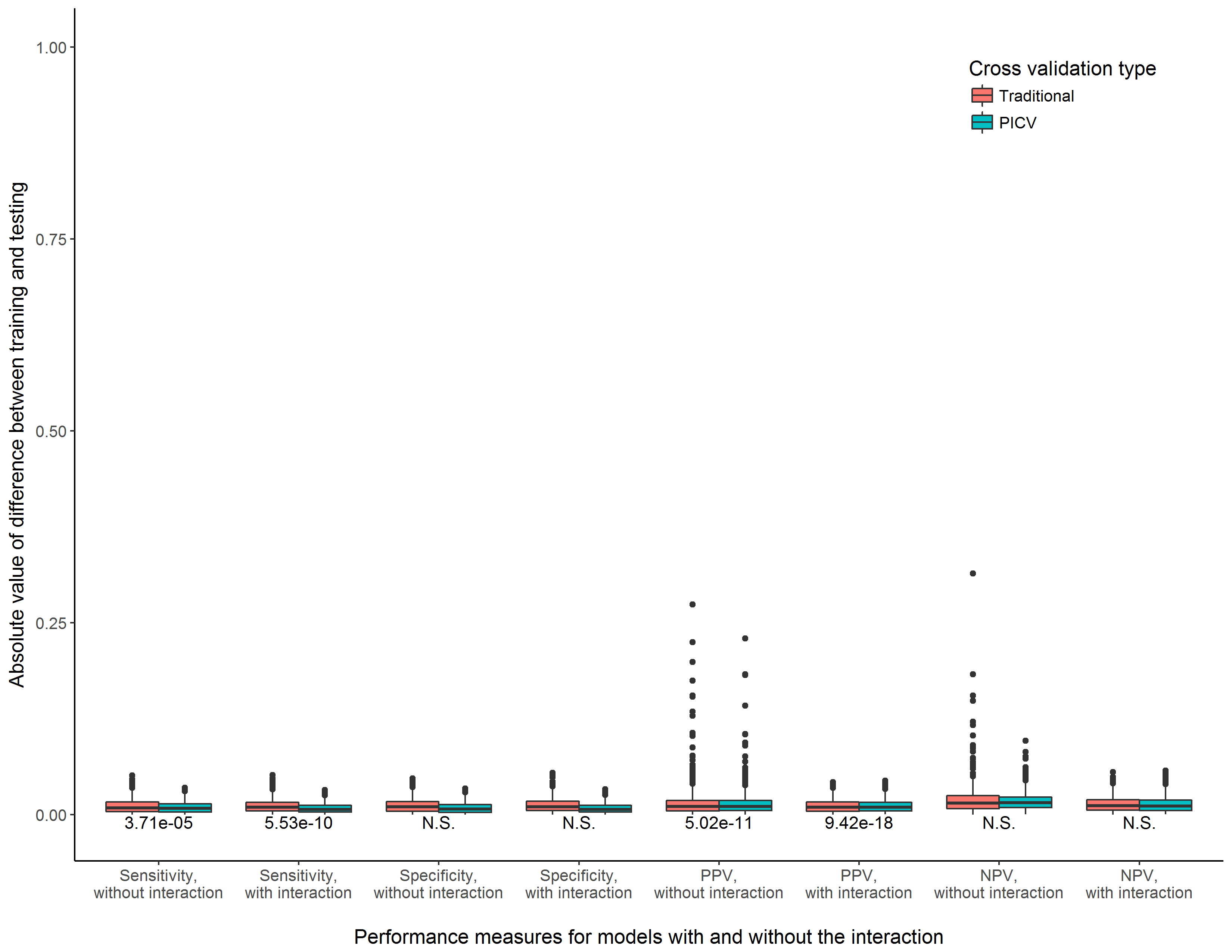


**Figure S40.** Consistency of training and testing performance measures for models with and without the interaction term, comparing a traditional cross validation procedure to PICV. Experimental scenario 10, prevalence = 0.1, n = 10000


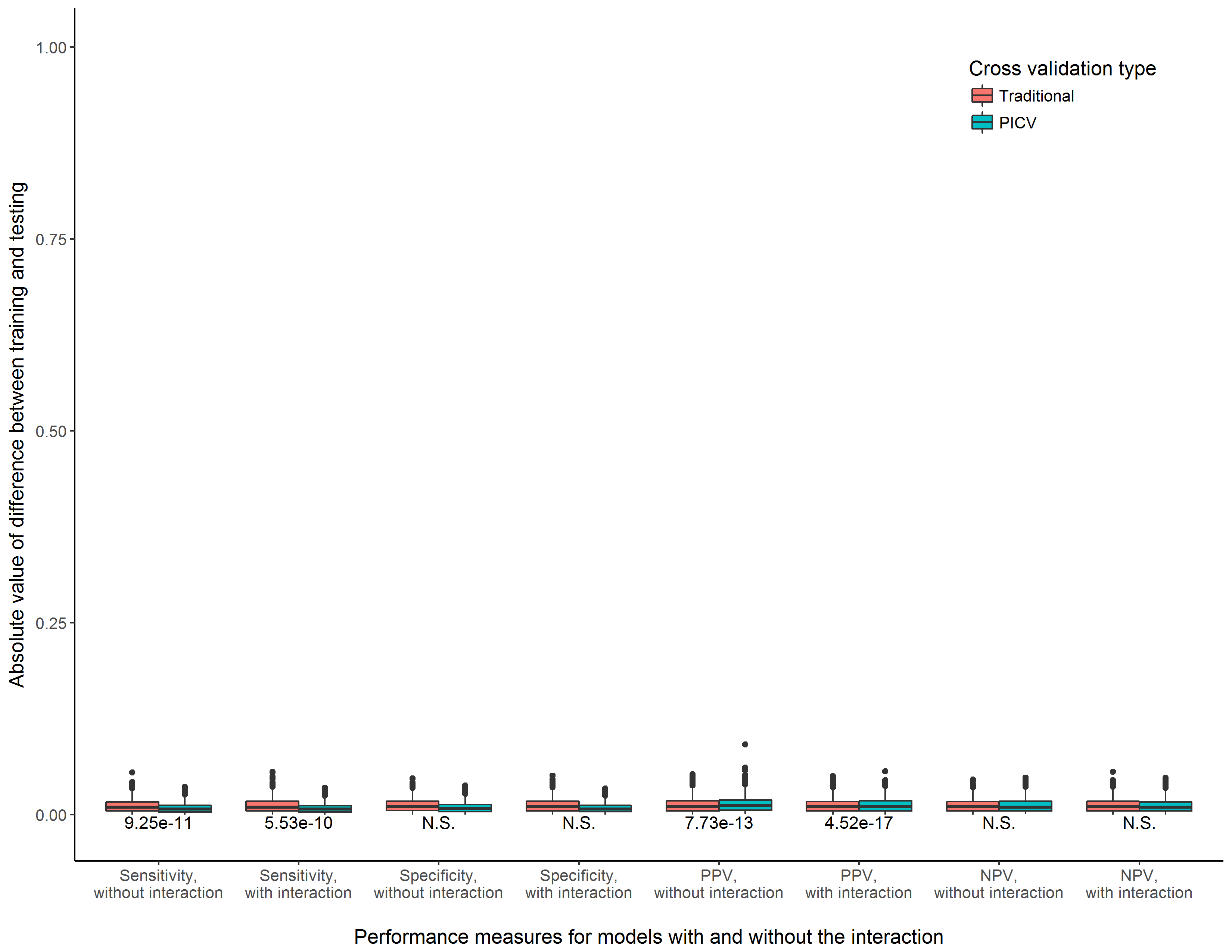


**Figure S41.** Consistency of training and testing performance measures for models with and without the interaction term, comparing a traditional cross validation procedure to PICV. Experimental scenario 11, prevalence = 0.1, n = 10000

**
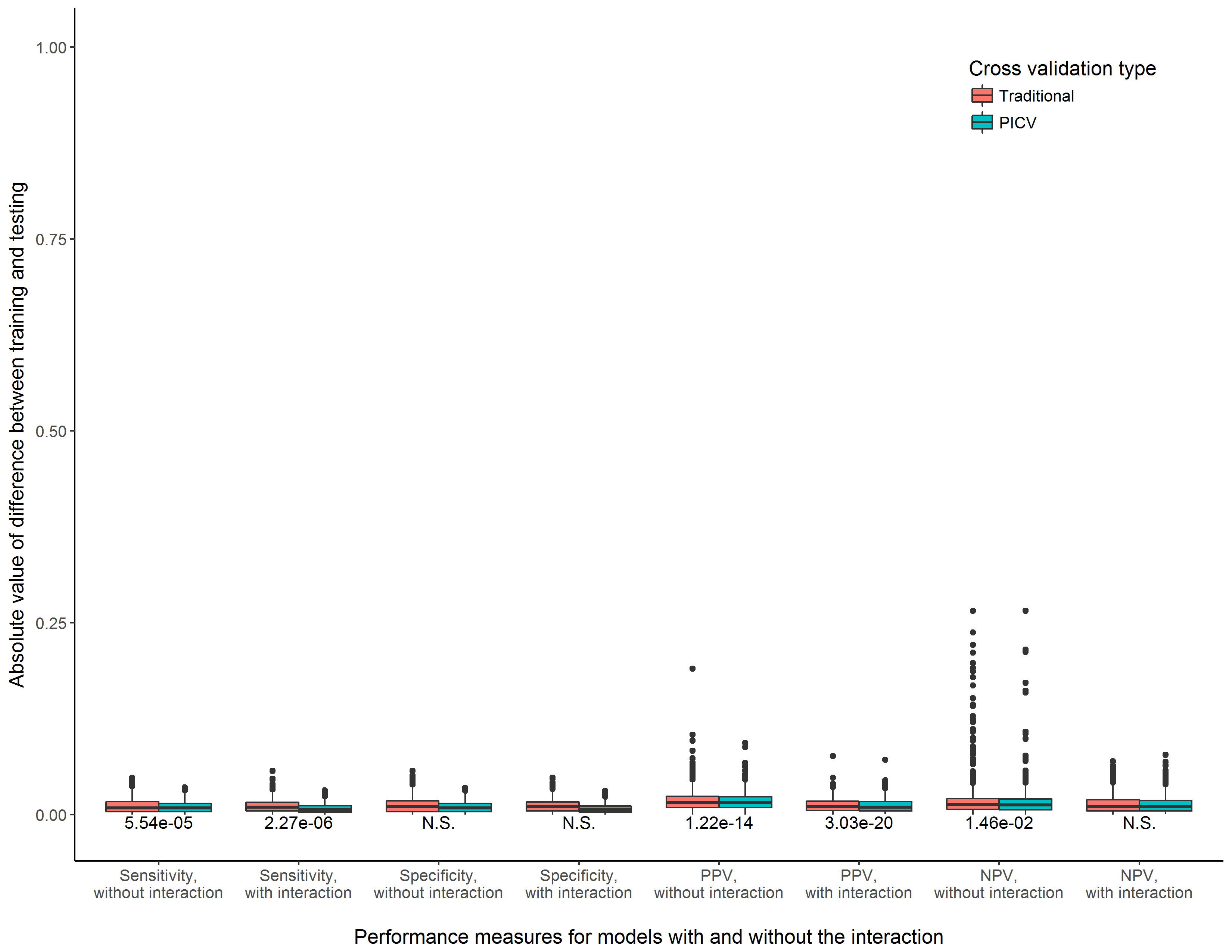
**

**Figure S42.** Consistency of training and testing performance measures for models with and without the interaction term, comparing a traditional cross validation procedure to PICV. Experimental scenario 12, prevalence = 0.1, n = 10000

**
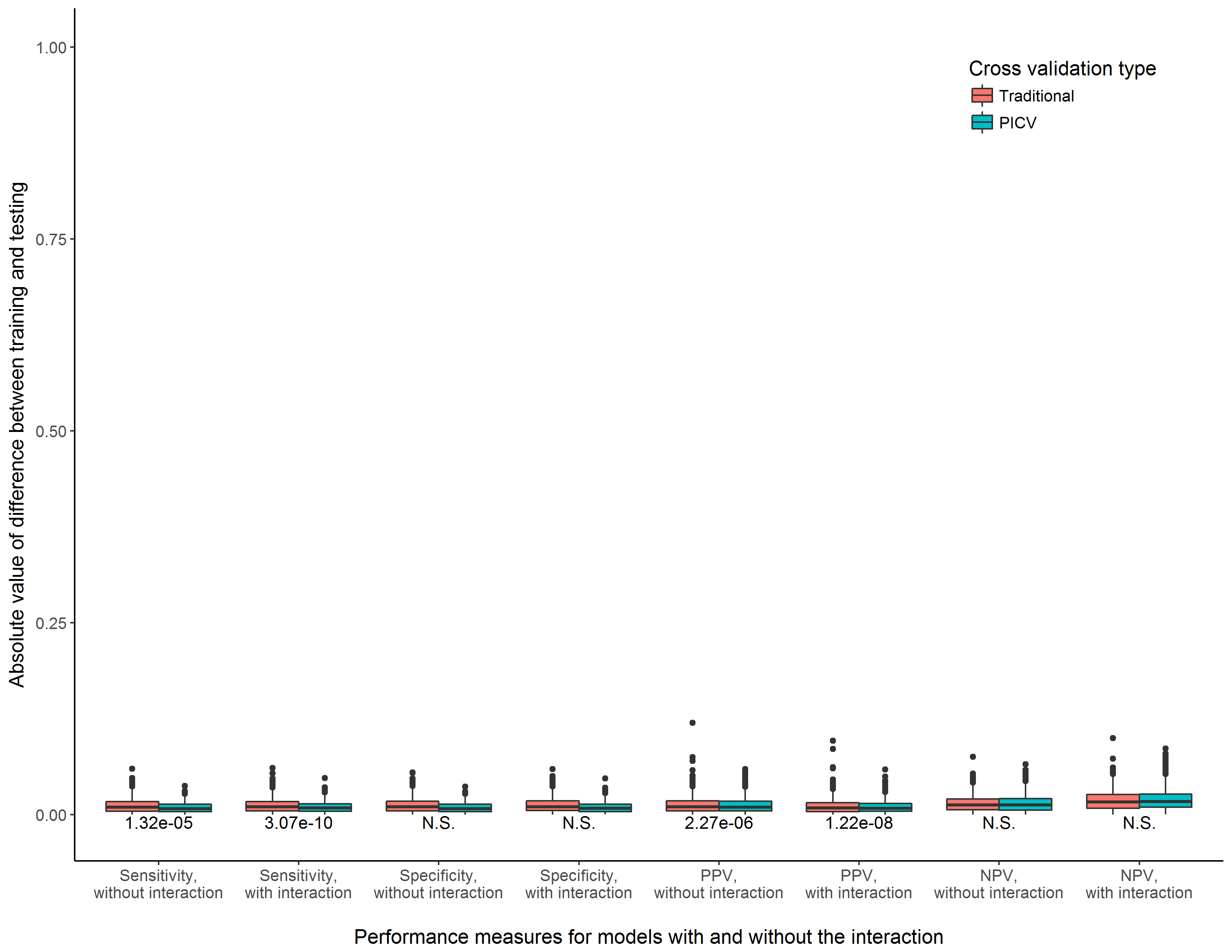
**

**Figure S43.** Consistency of training and testing performance measures for models with and without the interaction term, comparing a traditional cross validation procedure to PICV. Experimental scenario 13, prevalence = 0.1, n = 10000

**
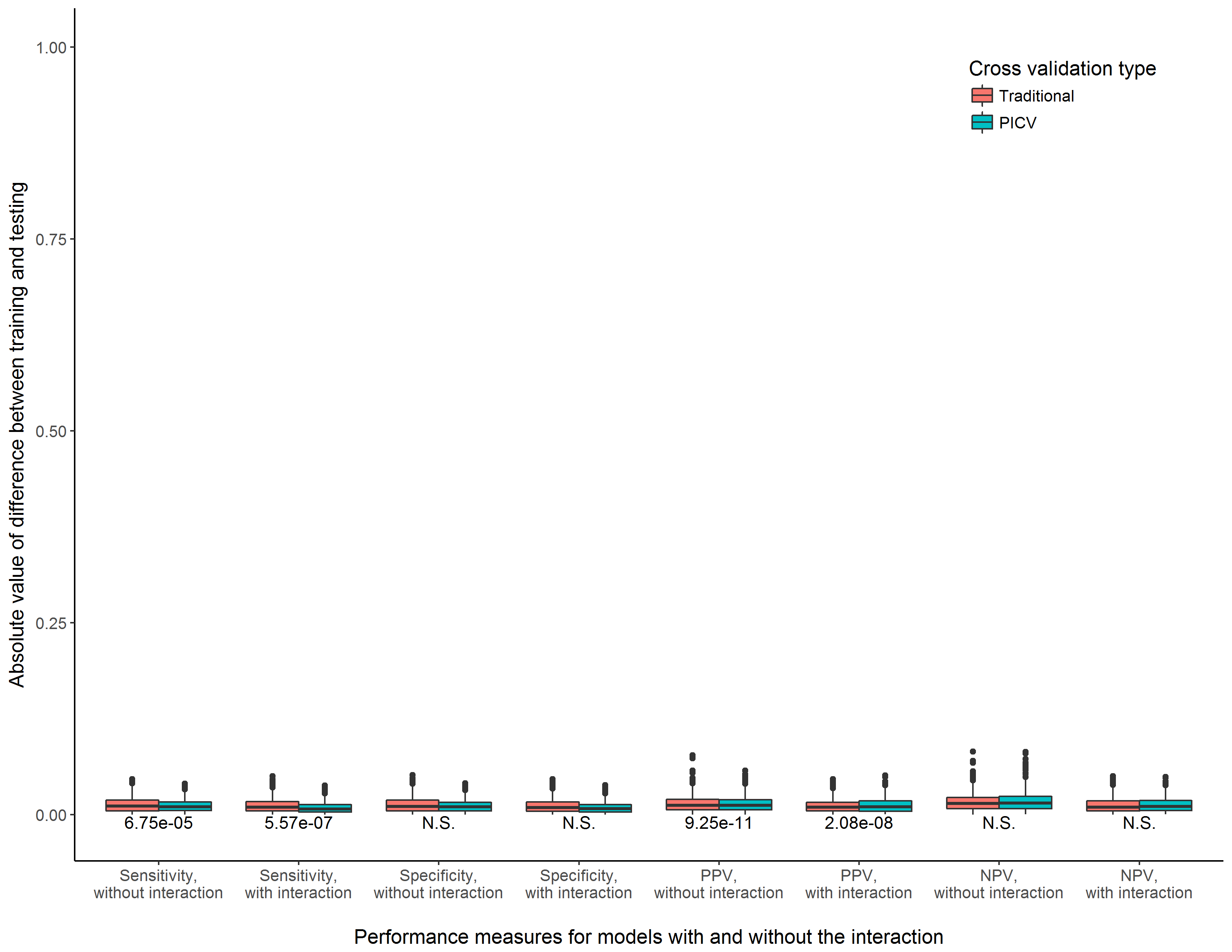
**

**Figure S44.** Consistency of training and testing performance measures for models with and without the interaction term, comparing a traditional cross validation procedure to PICV. Experimental scenario 14, prevalence = 0.1, n = 10000

**
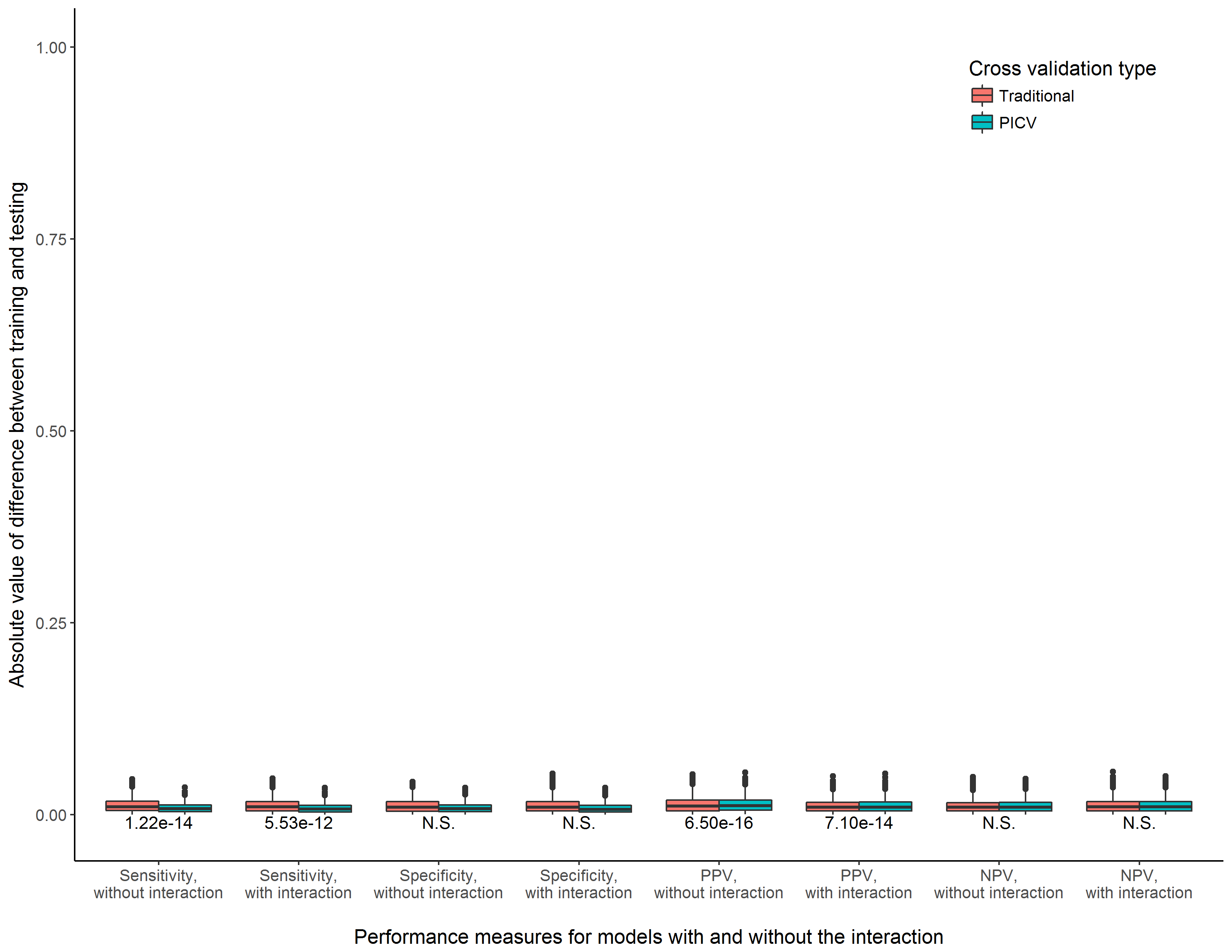
**

**Figure S45.** Consistency of training and testing performance measures for models with and without the interaction term, comparing a traditional cross validation procedure to PICV. Experimental scenario 15, prevalence = 0.1, n = 10000


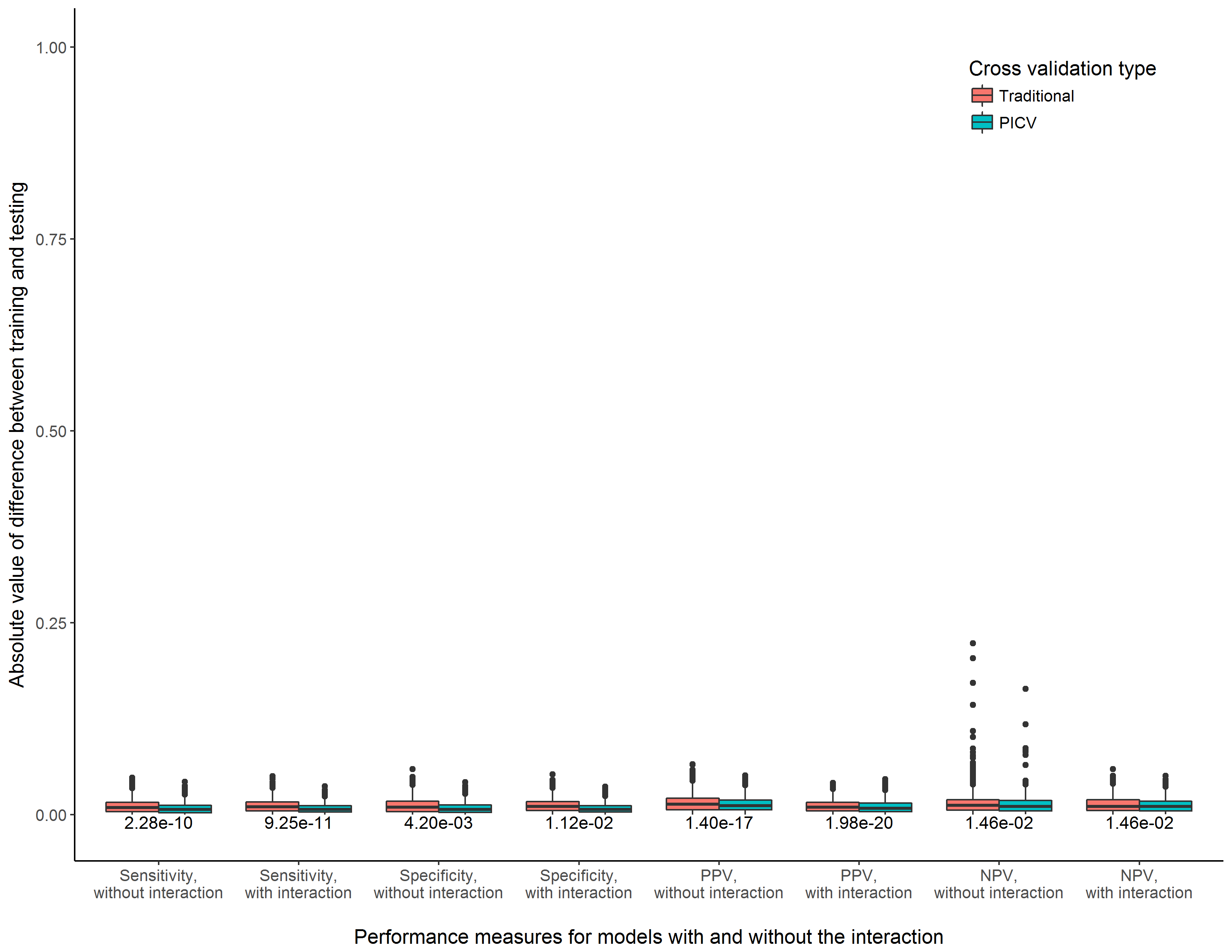


**Figure S46.** Consistency of training and testing performance measures for models with and without the interaction term, comparing a traditional cross validation procedure to PICV. Experimental scenario 1, prevalence = 0.02, n = 10000


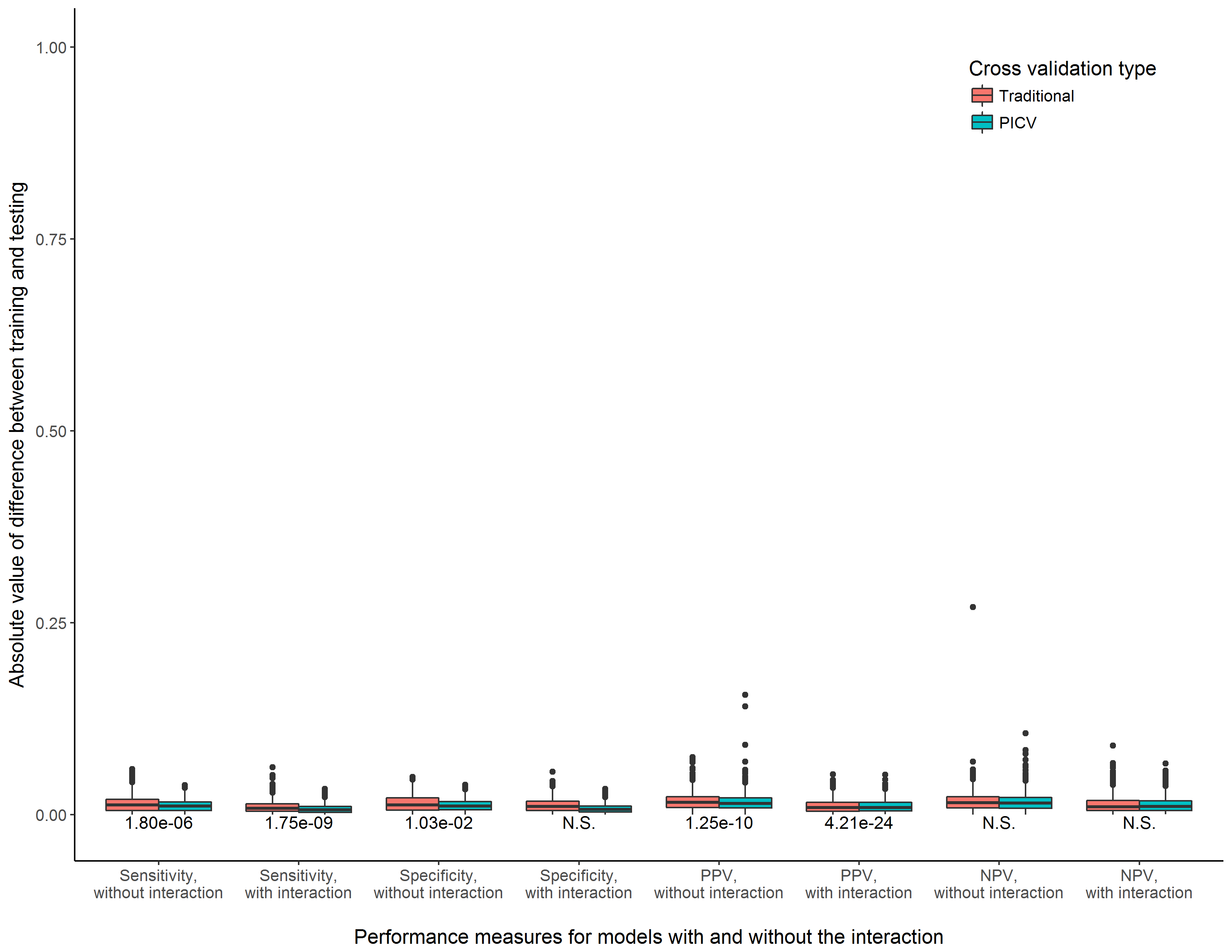


**Figure S47.** Consistency of training and testing performance measures for models with and without the interaction term, comparing a traditional cross validation procedure to PICV. Experimental scenario 2, prevalence = 0.02, n = 10000


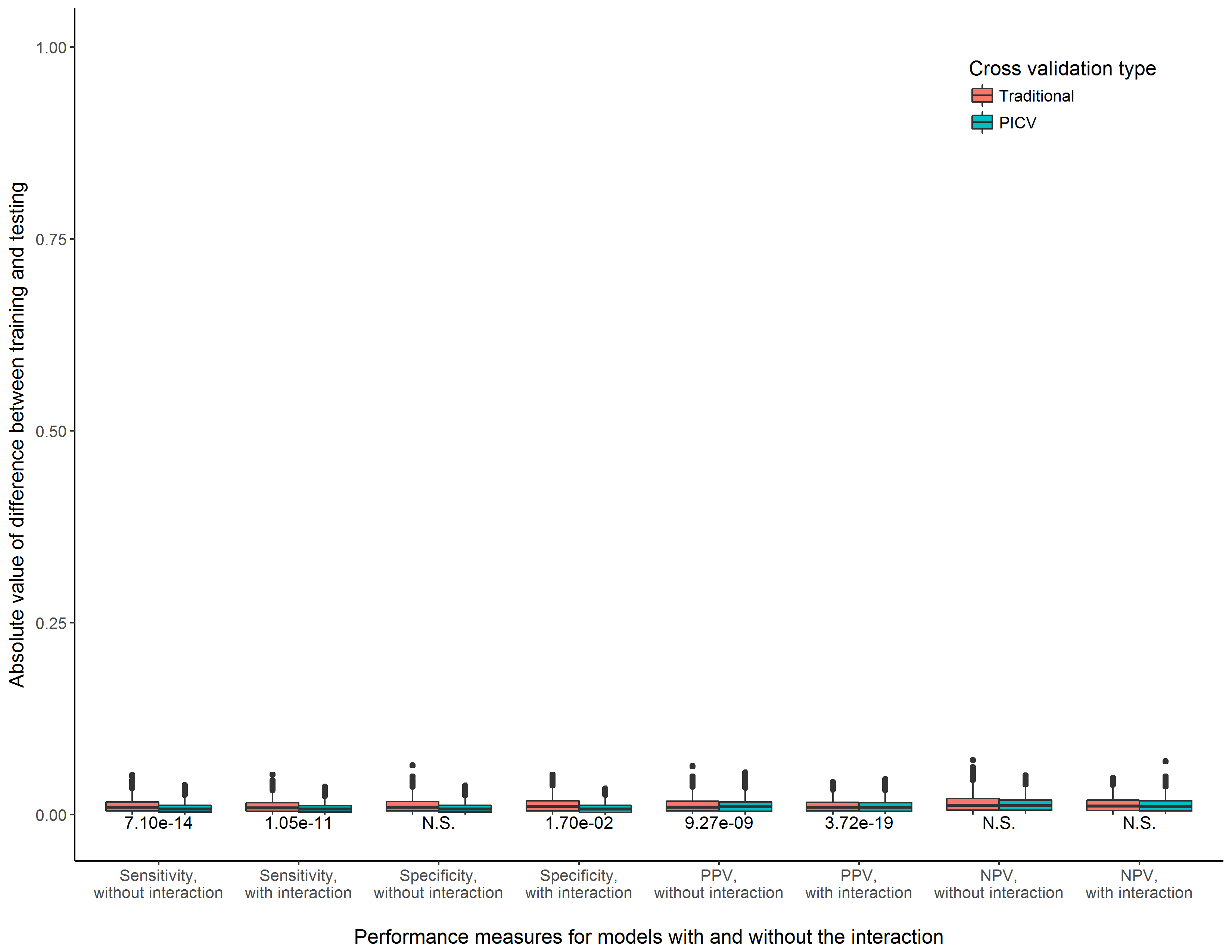


**Figure S48.** Consistency of training and testing performance measures for models with and without the interaction term, comparing a traditional cross validation procedure to PICV. Experimental scenario 3, prevalence = 0.02, n = 10000


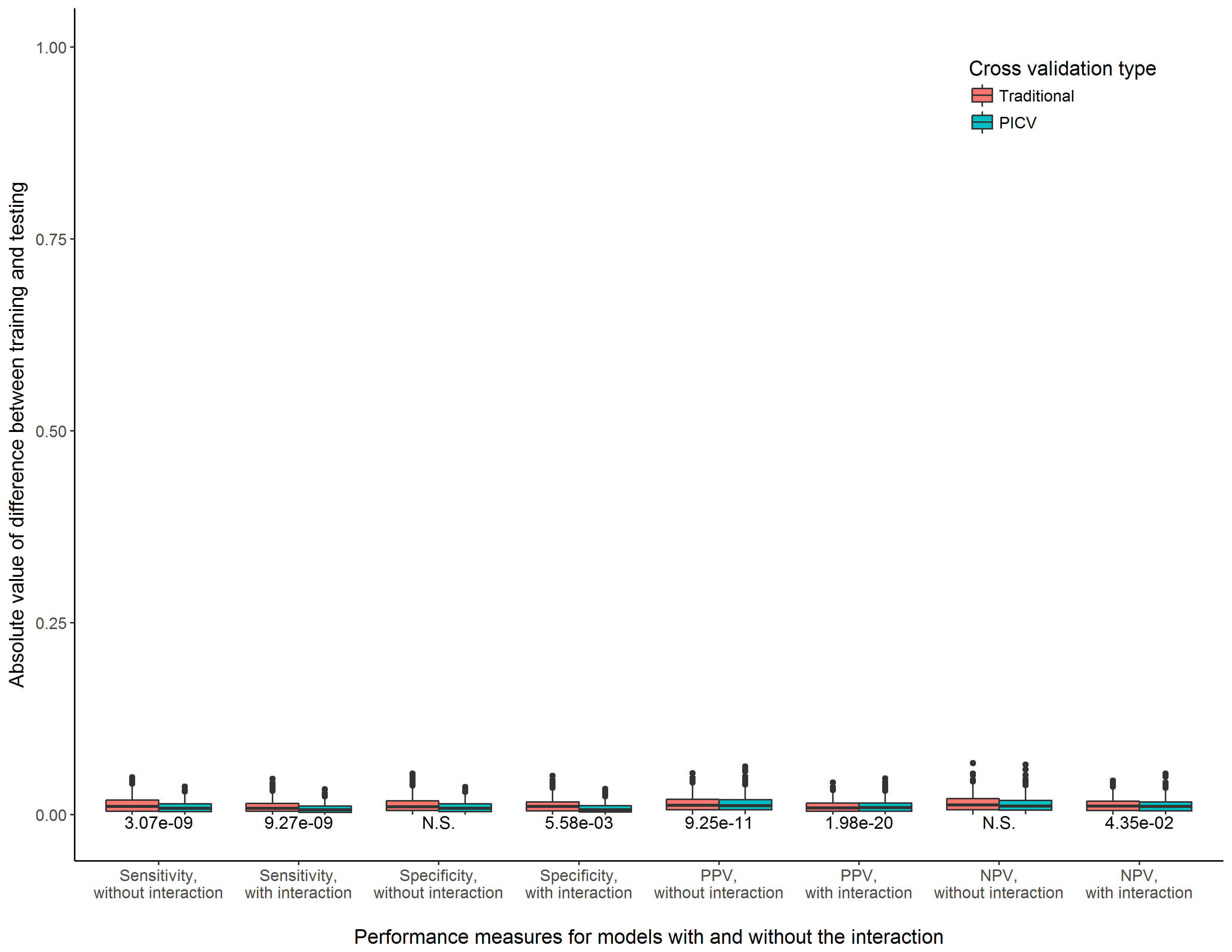


**Figure S49.** Consistency of training and testing performance measures for models with and without the interaction term, comparing a traditional cross validation procedure to PICV. Experimental scenario 4, prevalence = 0.02, n = 10000

**
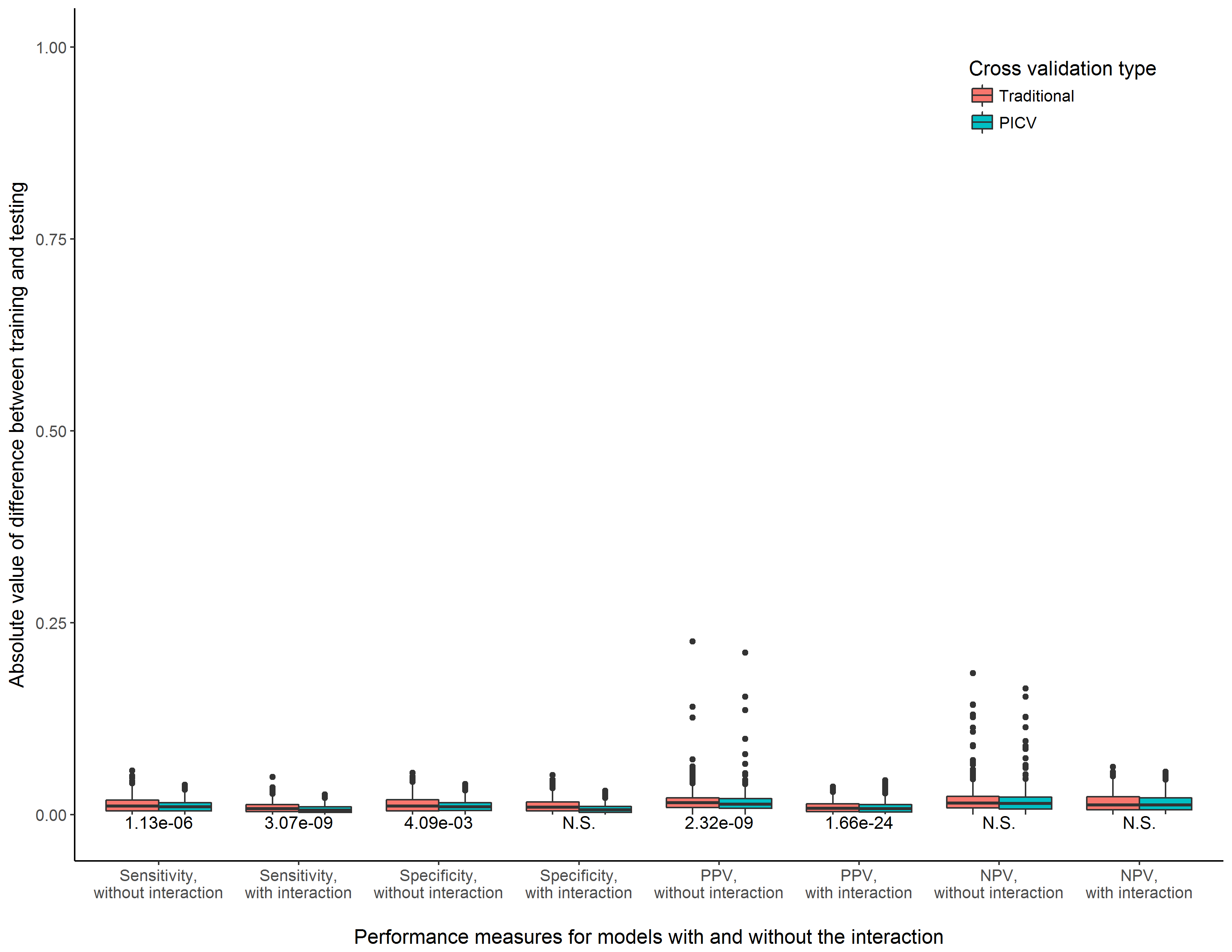
**

**Figure S50.** Consistency of training and testing performance measures for models with and without the interaction term, comparing a traditional cross validation procedure to PICV. Experimental scenario 5, prevalence = 0.02, n = 10000


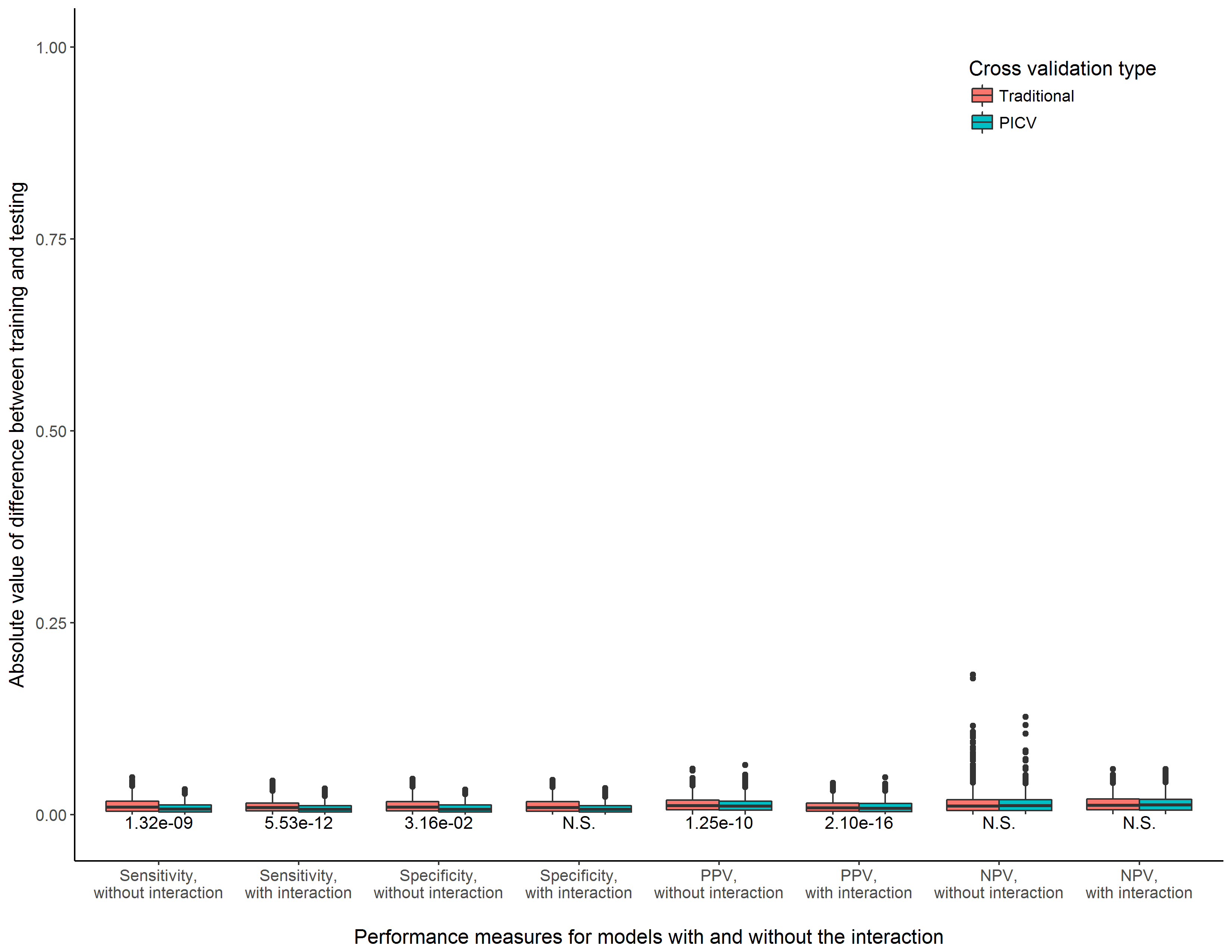


**Figure S51.** Consistency of training and testing performance measures for models with and without the interaction term, comparing a traditional cross validation procedure to PICV. Experimental scenario 6, prevalence = 0.02, n = 10000

**
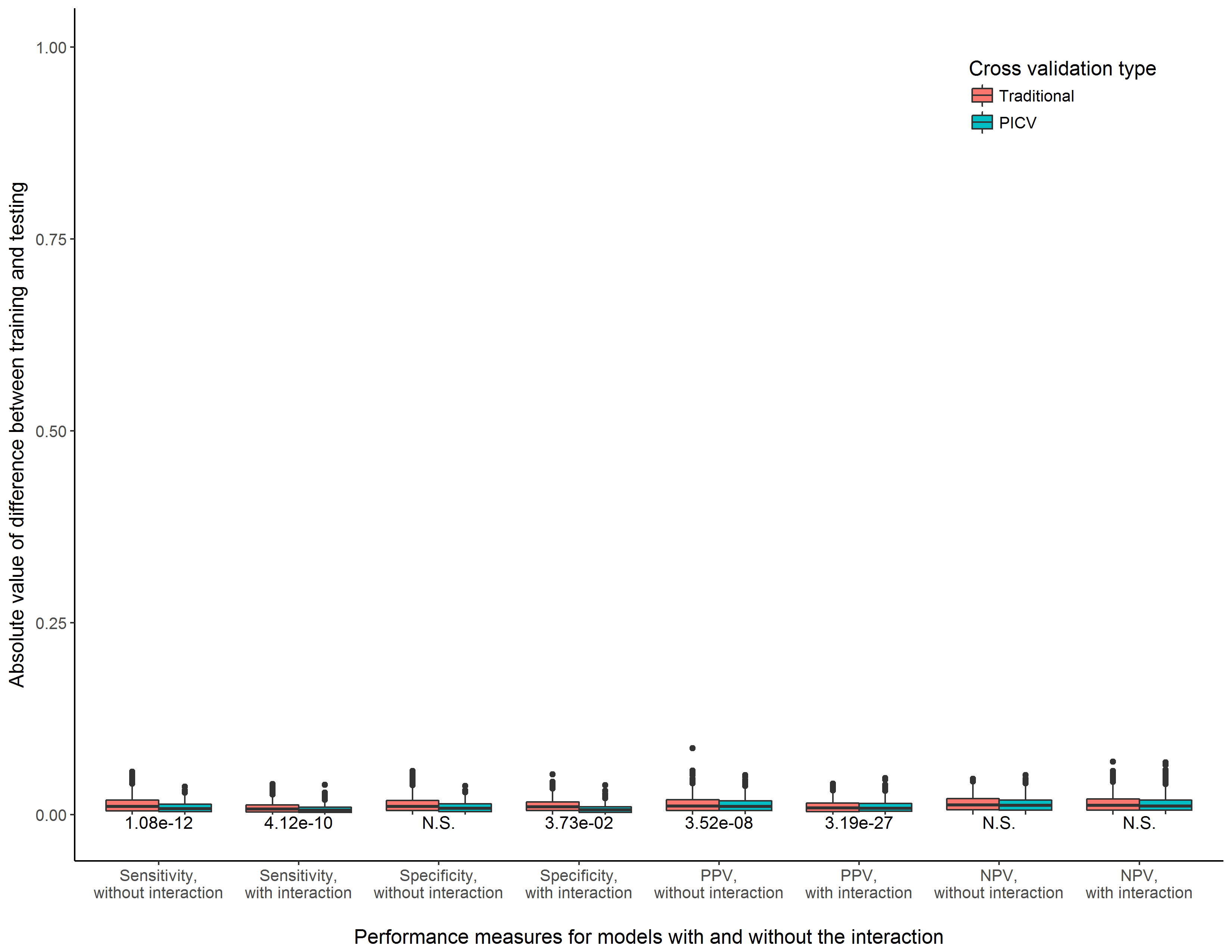
**

**Figure S52.** Consistency of training and testing performance measures for models with and without the interaction term, comparing a traditional cross validation procedure to PICV. Experimental scenario 7, prevalence = 0.02, n = 10000


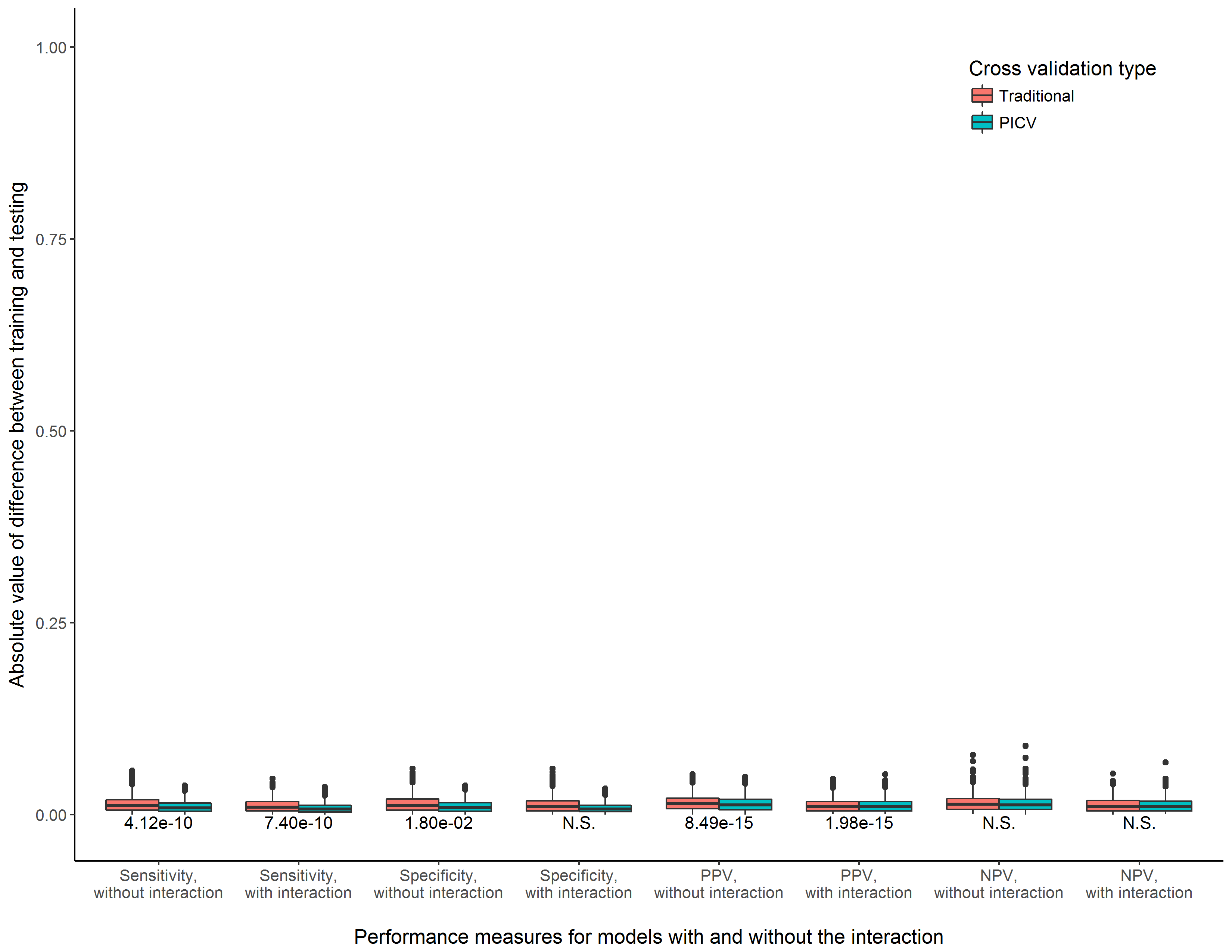


**Figure S53.** Consistency of training and testing performance measures for models with and without the interaction term, comparing a traditional cross validation procedure to PICV. Experimental scenario 8, prevalence = 0.02, n = 10000


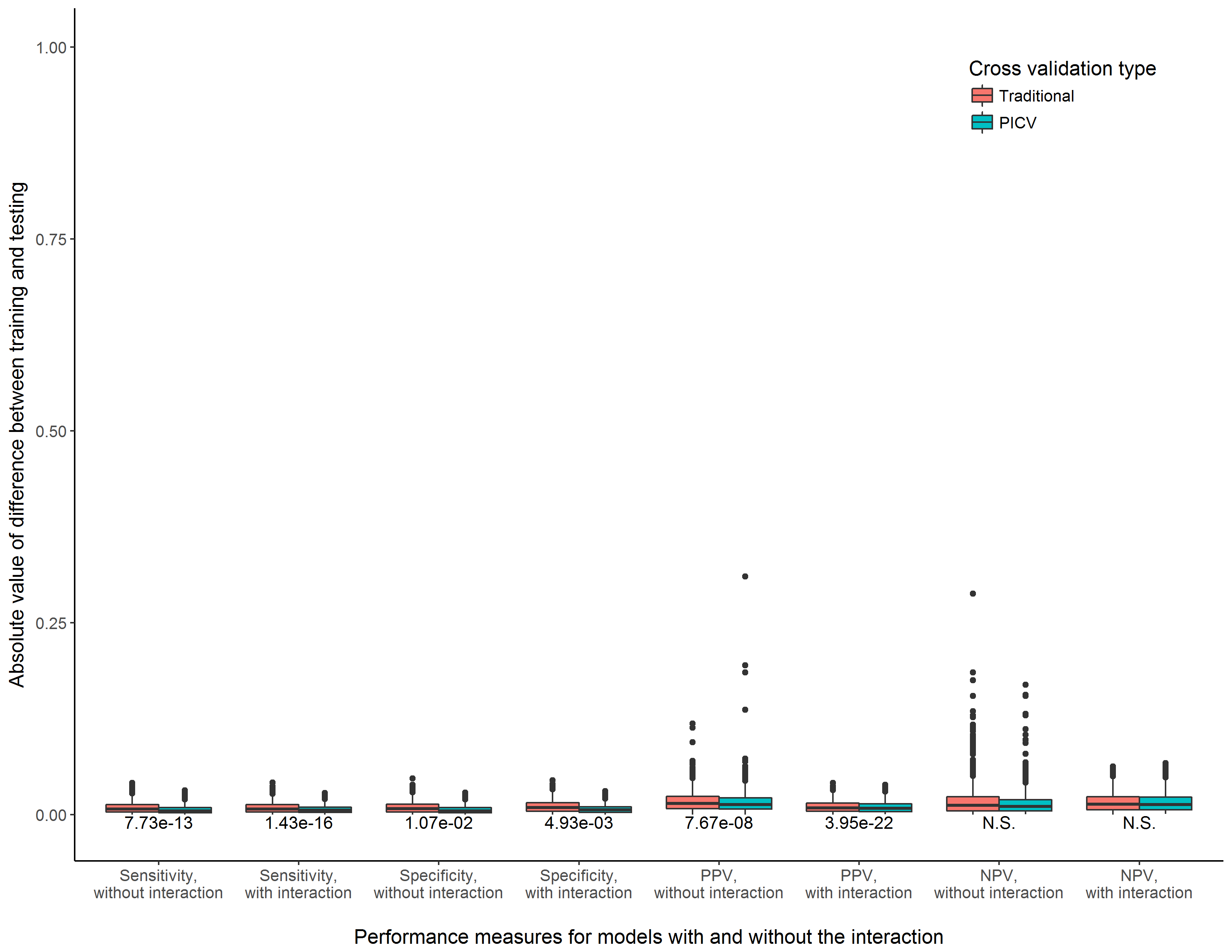


**Figure S54.** Consistency of training and testing performance measures for models with and without the interaction term, comparing a traditional cross validation procedure to PICV. Experimental scenario 9, prevalence = 0.02, n = 10000


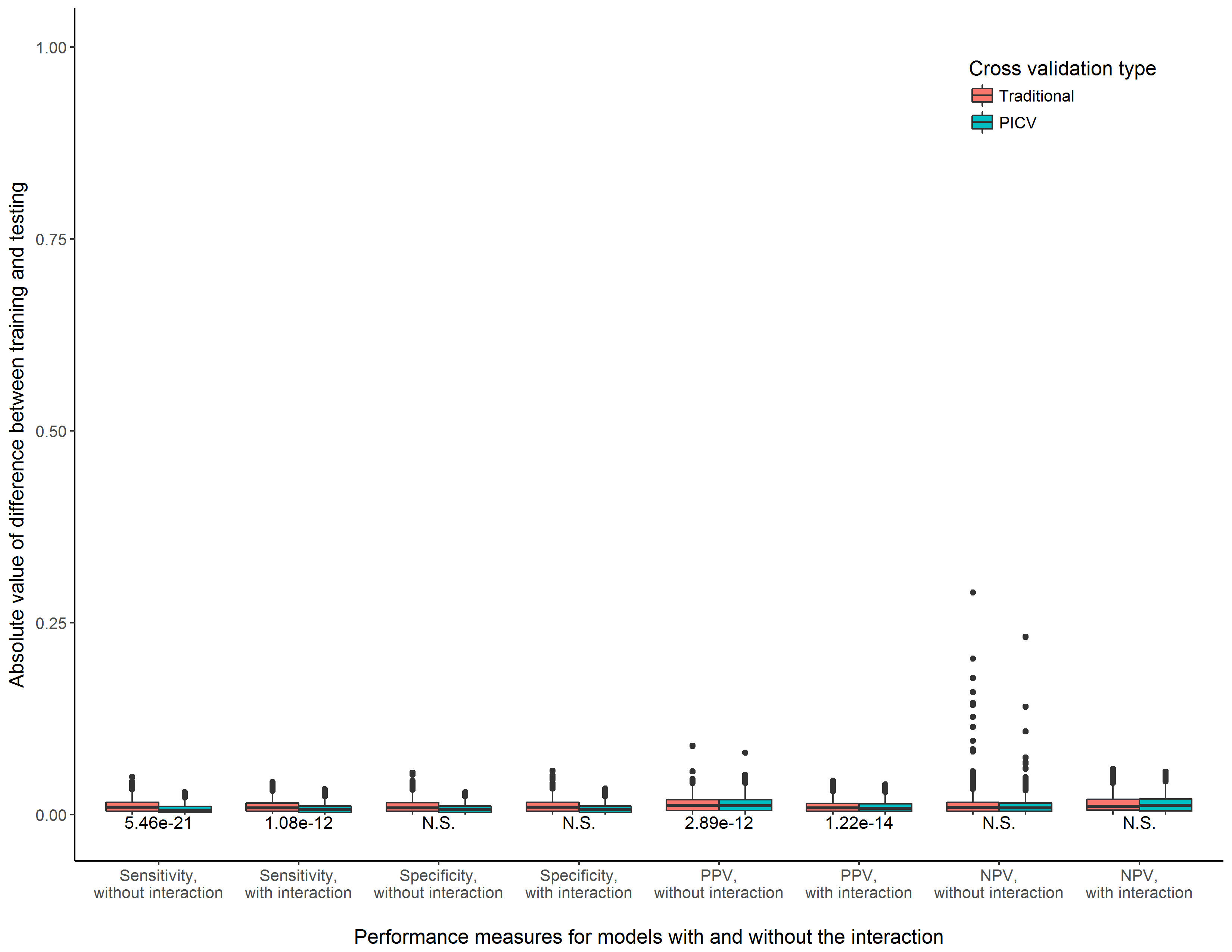
 **Figure S55.** Consistency of training and testing performance measures for models with and without the interaction term, comparing a traditional cross validation procedure to PICV. Experimental scenario 10, prevalence = 0.02, n = 10000

**
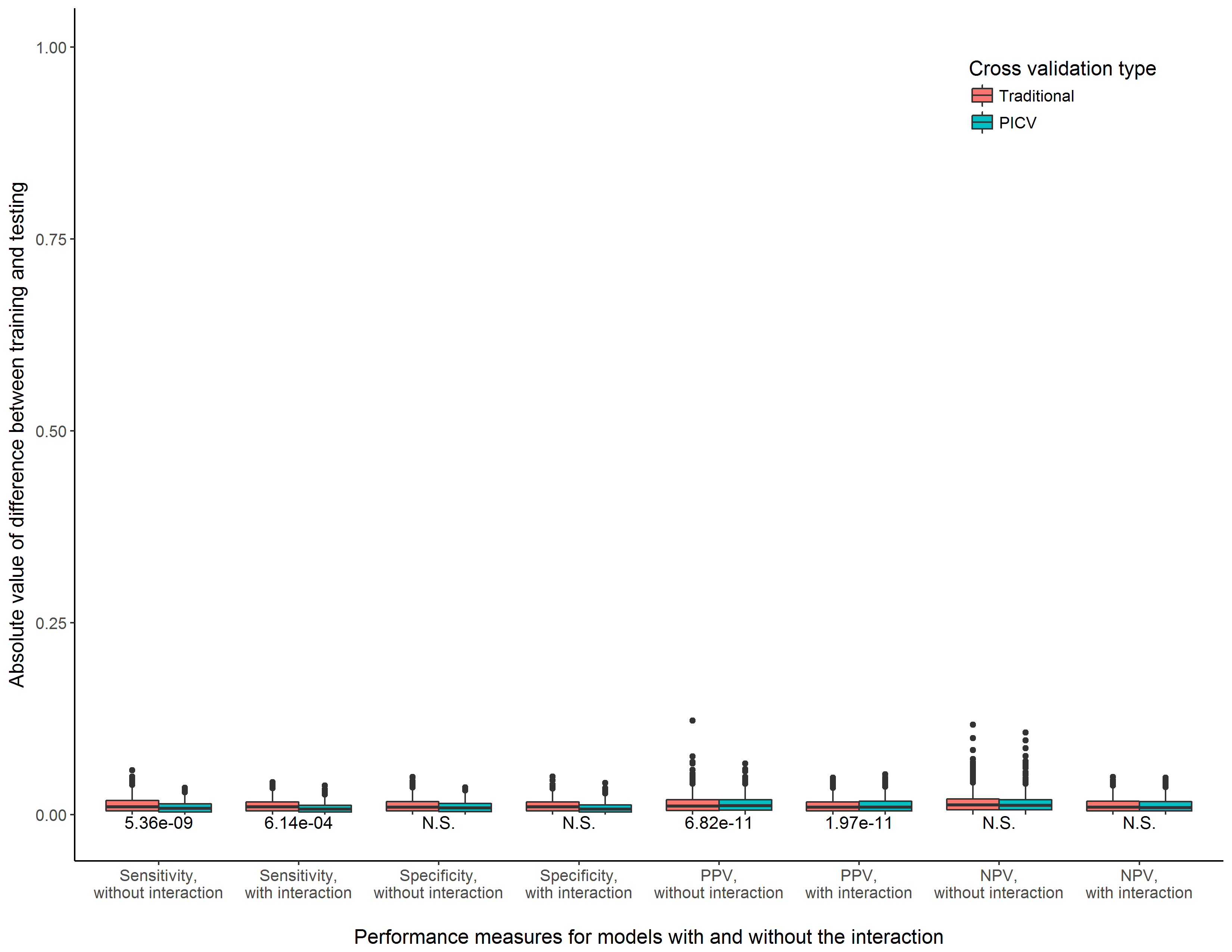
**

**Figure S56.** Consistency of training and testing performance measures for models with and without the interaction term, comparing a traditional cross validation procedure to PICV. Experimental scenario 11, prevalence = 0.02, n = 10000


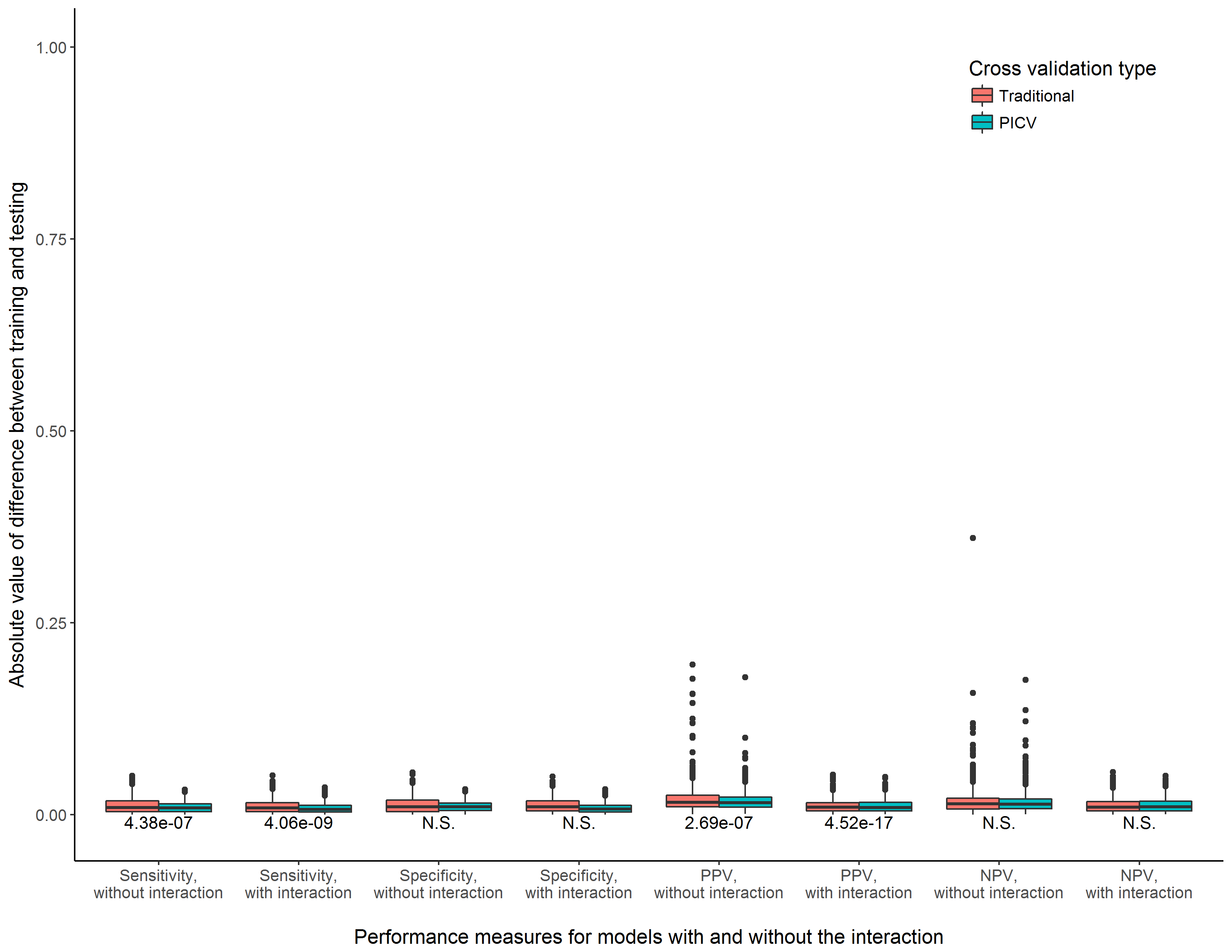


**Figure S57.** Consistency of training and testing performance measures for models with and without the interaction term, comparing a traditional cross validation procedure to PICV. Experimental scenario 12, prevalence = 0.02, n = 10000


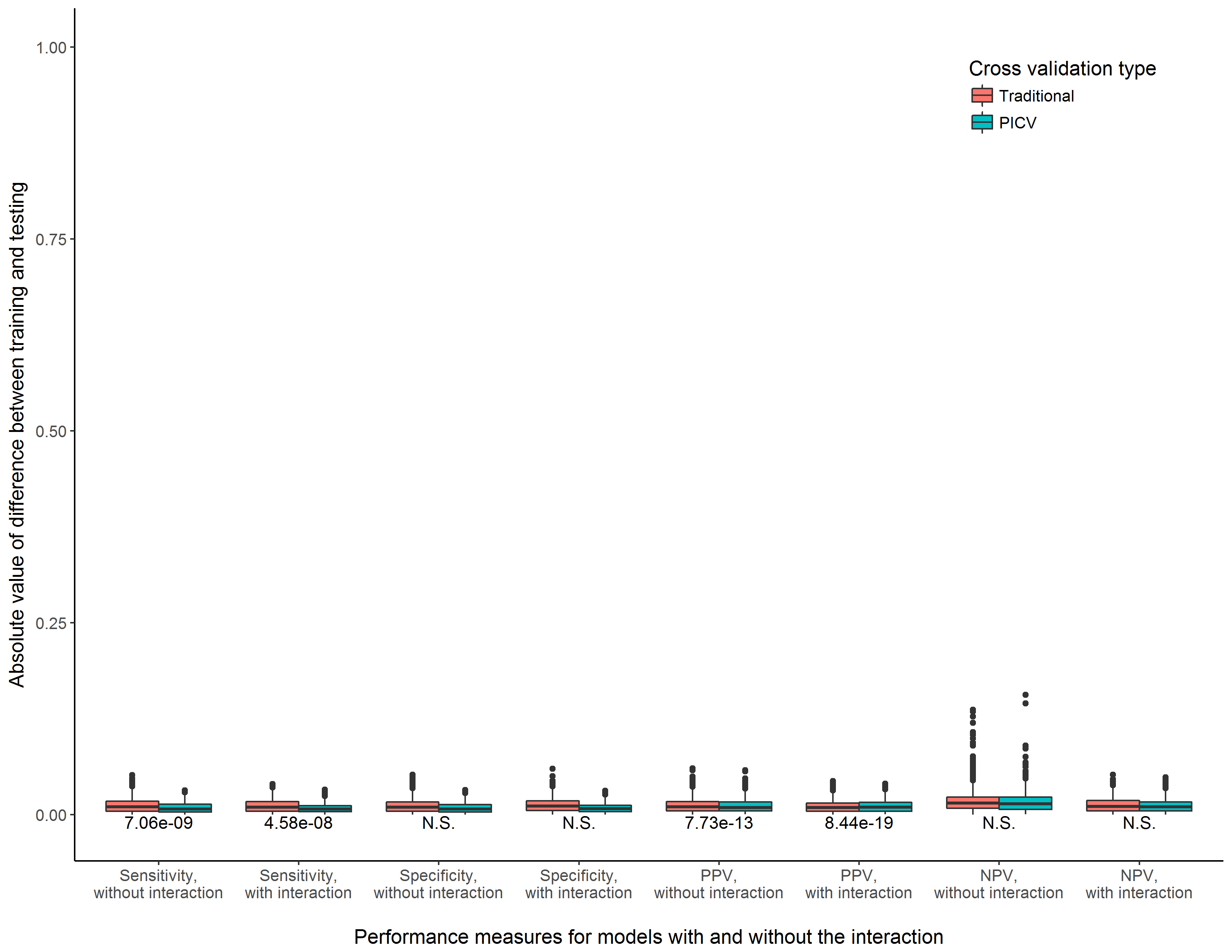


**Figure S58.** Consistency of training and testing performance measures for models with and without the interaction term, comparing a traditional cross validation procedure to PICV. Experimental scenario 13, prevalence = 0.02, n = 10000


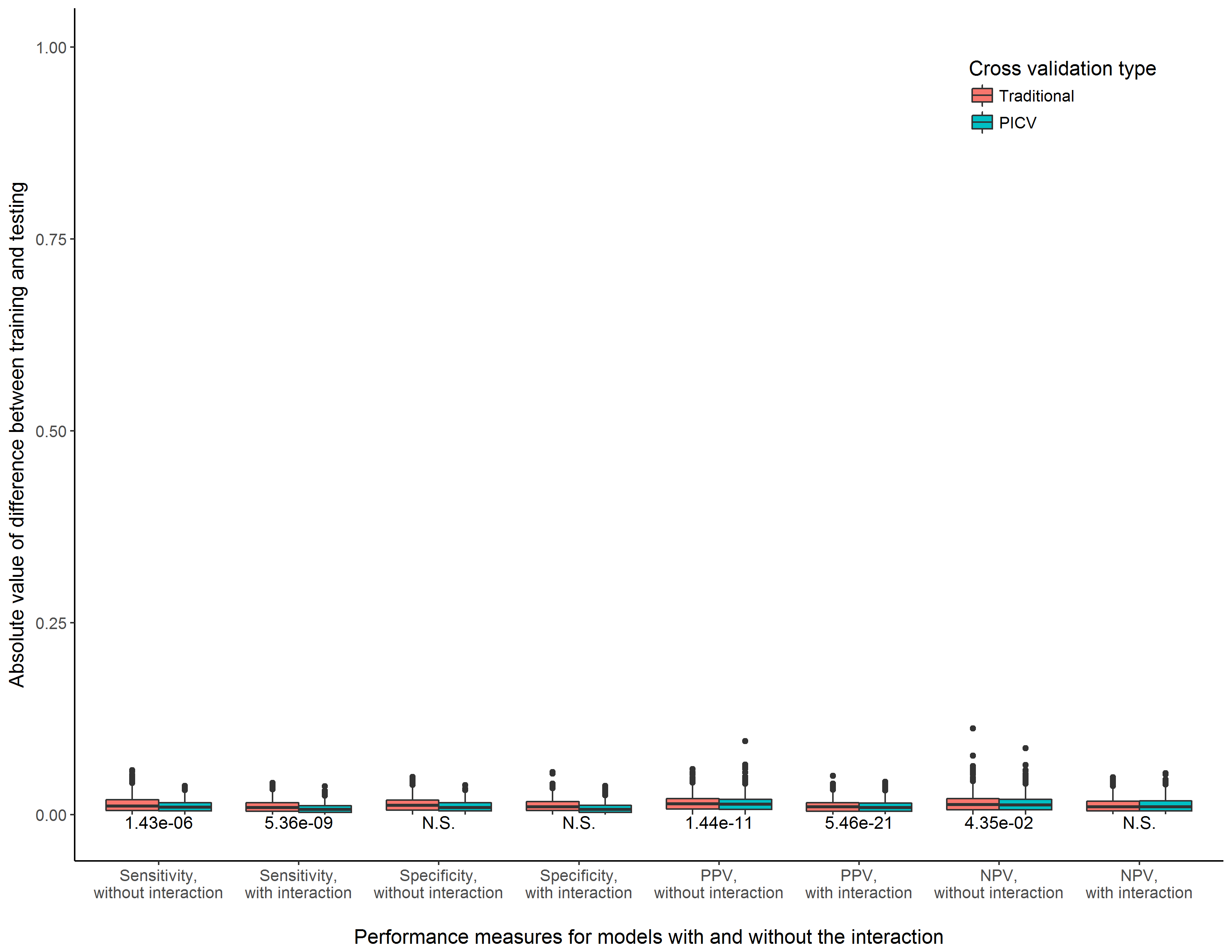


**Figure S59.** Consistency of training and testing performance measures for models with and without the interaction term, comparing a traditional cross validation procedure to PICV. Experimental scenario 14, prevalence = 0.02, n = 10000


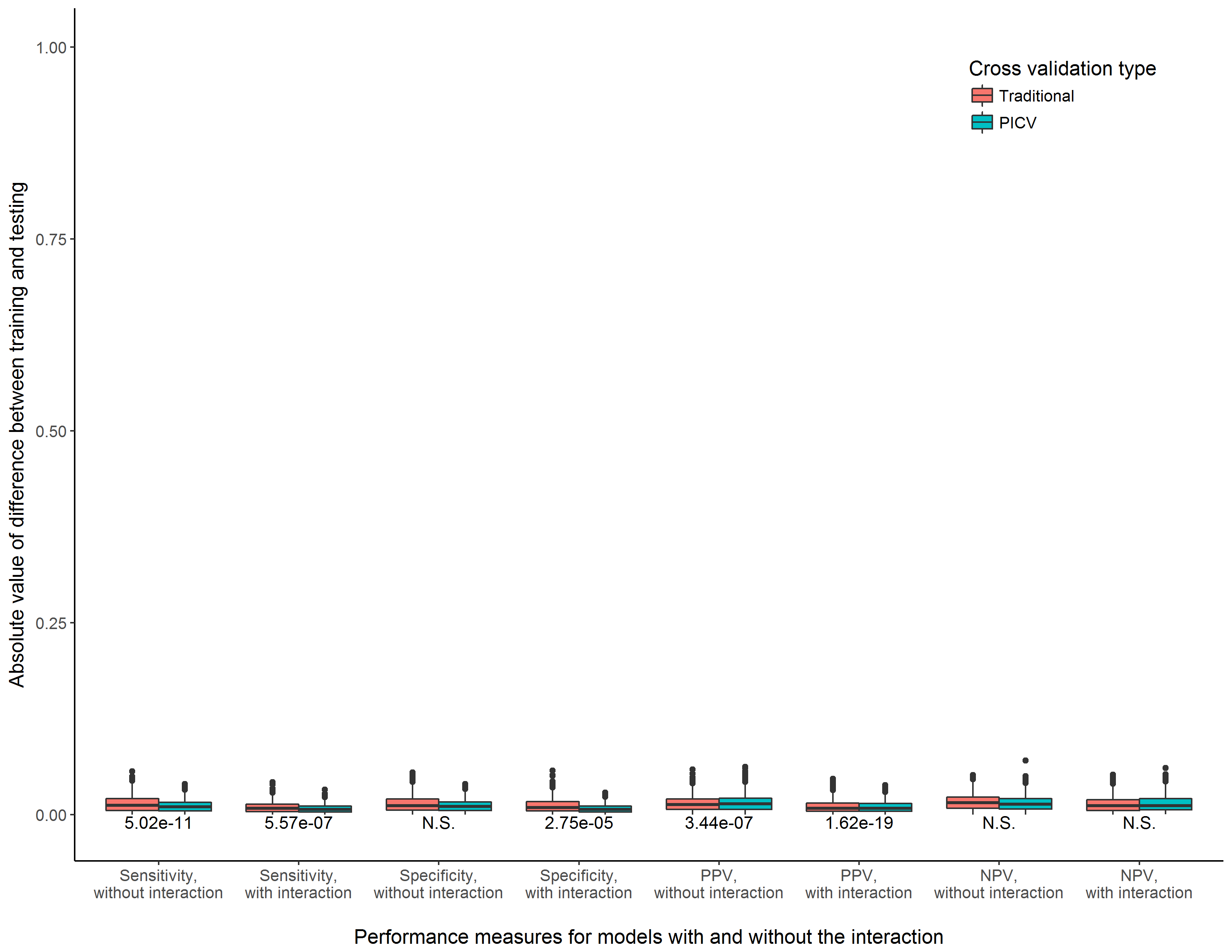
 **Figure S60.** Consistency of training and testing performance measures for models with and without the interaction term, comparing a traditional cross validation procedure to PICV. Experimental scenario 15, prevalence = 0.02, n = 10000
